# Peering Into *Candida albicans* Pir Protein Function and Comparative Genomics of the Pir Family

**DOI:** 10.3389/fcimb.2022.836632

**Published:** 2022-03-18

**Authors:** Jisoo Kim, Soon-Hwan Oh, Rubi Rodriguez-Bobadilla, Vien M. Vuong, Vit Hubka, Xiaomin Zhao, Lois L. Hoyer

**Affiliations:** ^1^ Department of Pathobiology, College of Veterinary Medicine, University of Illinois at Urbana-Champaign, Urbana, IL, United States; ^2^ Department of Biology, Millikin University, Decatur, IL, United States; ^3^ Department of Computer Science, University of Illinois at Urbana-Champaign, Urbana, IL, United States; ^4^ Department of Botany, Faculty of Science, Charles University, Prague, Czechia; ^5^ Laboratory of Fungal Genetics and Metabolism, Institute of Microbiology, Czech Academy of Sciences, Prague, Czechia

**Keywords:** fungal cell wall, *PIR*, *CIS*, *Candida*, *Saccharomyces cerevisiae*, β-1,3-glucan

## Abstract

The fungal cell wall, comprised primarily of protein and polymeric carbohydrate, maintains cell structure, provides protection from the environment, and is an important antifungal drug target. Pir proteins (proteins with internal repeats) are linked to cell wall β-1,3-glucan and are best studied in *Saccharomyces cerevisiae*. Sequential deletion of *S. cerevisiae PIR* genes produces strains with increasingly notable cell wall damage. However, a true null mutant lacking all five *S. cerevisiae PIR* genes was never constructed. Because only two *PIR* genes (*PIR1*, *PIR32*) were annotated in the *Candida albicans* genome, the initial goal of this work was to construct a true *Δpir/Δpir* null strain in this species. Unexpectedly, the phenotype of the null strain was almost indistinguishable from its parent, leading to the search for other proteins with Pir function. Bioinformatic approaches revealed nine additional *C. albicans* proteins that share a conserved Pir functional motif (minimally DGQ). Examination of the protein sequences revealed another conserved motif (QFQFD) toward the C-terminal end of each protein. Sequence similarities and presence of the conserved motif(s) were used to identify a set of 75 proteins across 16 fungal species that are proposed here as Pir proteins. The Pir family is greatly expanded in *C. albicans* and *C. dubliniensis* compared to other species and the orthologs are known to have specialized function during chlamydospore formation. Predicted Pir structures showed a conserved core of antiparallel beta-sheets and sometimes-extensive loops that contain amino acids with the potential to form linkages to cell wall components. Pir phylogeny demonstrated emergence of specific ortholog groups among the fungal species. Variation in gene expression patterns was noted among the ortholog groups during growth in rich medium. *PIR* allelic variation was quite limited despite the presence of a repeated sequence in many loci. Results presented here demonstrate that the Pir family is larger than previously recognized and lead to new hypotheses to test to better understand Pir proteins and their role in the fungal cell wall.

## Introduction

The cell wall serves as a barrier between the fungal cell and its environment and has received considerable attention as a mediator of pathogenicity and an antifungal drug target (Chaffin et al., 1998; Ruiz-Herrera et al., 2006; Chaffin, 2008; Gow and Hube, 2012). The fungal cell wall is composed of polymeric carbohydrates (e.g. chitin and β-glucans), mannoproteins, and a small amount of lipid (Northcote and Horne, 1952) roughly organized into distinct layers that surround the fungal cell membrane. Chitin comprises the innermost layer, with β-1,3- and β-1,6-glucans immediately external to chitin; linkages exist among the various cell wall components (Kapteyn et al., 2000). Mannoproteins in the outermost cell wall layer may be covalently or non-covalently bound to the β-glucans. Among the covalently bound proteins are those that are transiently modified with a glycosyl-phosphatidylinositol (GPI) anchor then crosslinked *via* the anchor remnant to β-1,6-glucan, commonly referred to as the GPI-cell wall proteins (GPI-CWPs; Lu et al., 1994).

Proteins with internal repeats (abbreviated Pir) are also linked covalently to the fungal cell wall (Mrša et al., 1997; Kapteyn et al., 1999) and can be released with mild alkali treatment (Mrša and Tanner, 1999; Moukadiri and Zueco, 2001; Castillo et al., 2003). Most work characterizing the Pir proteins was conducted in *S. cerevisiae* during the late 1990s and early 2000s, around the time that the genome sequence was completed (Goffeau et al., 1996). The *S. cerevisiae* literature describes four Pir proteins (Mrša and Tanner, 1999; Mazáň et al., 2008) while a fifth is apparent in the *Saccharomyces* Genome Database (SGD; http://yeastgenome.org; Engel et al., 2014). *S. cerevisiae* Pir proteins possess a secretory signal peptide and a Kex2 processing site (Moukadiri et al., 1999). *S. cerevisiae* Pir proteins are extensively modified by O-glycosylation (Mrša et al., 1997). The basis for the Pir family name is the presence of a repeated sequence (minimally DGQ[I/V]Q) in each *S. cerevisiae* protein. Pir4, which has only one copy of the repeated sequence, was used to demonstrate formation of an ester linkage between the γ-carboxyl group of the first Q in the motif and β-1,3-glucan (Ecker et al., 2006). This linkage is labile to the mild alkali treatment that released Pir proteins from the yeast cell wall in their initial characterization (Mrša et al., 1997).

Various *S. cerevisiae* strains with combinations of *PIR* gene deletions were constructed, including a strain that lacked *PIR1*, *PIR2*, *PIR3*, and *PIR4* (Mrša and Tanner, 1999; Mazáň et al., 2008). Compared to parental controls, the *Δpir1 Δpir2 Δpir3 Δpir4* strain grew more slowly and had a large, irregular shape. The mutant strain showed increased sensitivity to SDS, calcofluor white, and Congo red suggesting defects in cell wall structure and stability. A true *S. cerevisiae* null mutant, in which all 5 *PIR* genes were deleted, was never reported.

Kandasamy et al. (2000) and Kapteyn et al. (2000) noted the presence of Pir-like proteins in the *C. albicans* cell wall. Annotation of the *Candida albicans* SC5314 genome sequence indicated two *PIR* genes, named *PIR1* and *PIR32*. Martínez et al. (2004) suggested that *PIR1* is an essential gene because a *Δpir1/Δpir1* strain could not be constructed. Heterozygous mutants (*PIR1/Δpir1*) had altered cellular morphology, and increased sensitivity to growth on calcofluor white and Congo red, suggesting cell wall defects. Another report described a similar phenotype resulting from deletion of both *PIR32* alleles (Bahnan et al., 2012).

The initial goal of this work was to attempt construction of a *Δpir* null strain to better understand Pir protein contributions to *C. albicans* cell wall structure. The unanticipated near-total lack of phenotypic difference between the *Δpir1/Δpir1 Δpir32/Δpir32* null mutant and its wild-type parent revealed the presence of a much-larger Pir protein family in *C. albicans*, defined by shared sequence motifs and similar predicted structures. Public genome and gene expression databases were used to further expand this picture of the Pir family in *C. albicans*, *S. cerevisiae*, and 14 other fungal species. Analyses presented here re-examine and advance knowledge about Pir proteins, while highlighting the next experimental questions to address to understand Pir function.

## Results

### An Unexpectedly Minimal Effect of *PIR* Deletion on *C. albicans* Phenotype

The original goal of this work was to attempt construction of a *C. albicans* double null mutant strain (i.e. lacking both alleles at each of the *PIR1* and *PIR32* loci). **Materials and Methods** includes details for successful construction of a *Δpir1-1/Δpir1-2* strain (named 3097), a *Δpir32-1/Δpir32-2* strain (3545), and a double null mutant *Δpir1-1/Δpir1-2 Δpir32-1/Δpir32-2* (3543) derived from parent strain SC5314 (Gillum et al., 1984). Phenotypic testing compared mutant strains to the parent using methods described for evaluation of previously reported *C. albicans Δpir* mutant strains (Martínez et al., 2004; Bahnan et al., 2012; **Table 1**). **Table 1** also summarizes observations from the previous publications and from the current study. Details are presented below and in the **Supplementary Material**.

**Table 1:** Comparison between published phenotypes for *C. albicans* strains with mutations in *PIR* genes and results from the current study.

	*PIR1-1/Δpir1-2* and *Δpir1-1/PIR1-2* (Martínez et al., 2004)	*Δpir32-1/Δpir32-2* (Bahnan et al., 2012)	Δpir1-1/Δpir1-2,* Δpir32-1/Δpir32-2, Δpir1-1/Δpir1-2 Δpir32-1/Δpir32-2
Parent (control) strain	CAI4	RM1000	SC5314
Mutation method	Auxotrophic markers	Auxotrophic markers	Recyclable drug resistance marker
Growth rate	Slower at 28°C and 37°C		No difference from parent
Yeast morphology	Elongated, clumped	Large number of filamentous cells	No difference from parent
Germ tube/hypha formation		Increased in liquid medium, no difference on solid medium	No significant strain effect in liquid or on solid medium
Osmotic stress		Increased resistance at 1.5 M NaCl	No difference at 1.5 M NaCl
Oxidative stress		Increased resistance at 50 mM hydrogen peroxide	No difference from parent at 50 mM hydrogen peroxide
Heat shock		No difference at 42°C	No difference at 42°C
Antifungal resistance		No difference for Amphotericin B, caspofungin, fluconazole by E test	Differences of one or two dilutions for anidulafungin and itraconazole using Sensititre
SDS		Increased resistance at 0.015%	No difference at 0.03%
Cystamine			No difference at 50 mM
Calcofluor white	Increased sensitivity at 150 μg/ml	No difference at 50 μg/ml	Growth-medium-dependent slightly increased sensitivity at 150 μg/ml
Congo red	Increased sensitivity at 30 μg/ml	Increased sensitivity at 50 μg/ml	Inconsistent/non-repeatable results; growth-medium-dependent slightly increased sensitivity at 30 μg/ml
Adhesion		Delayed adhesion to HT-29 epithelial cells	No difference for adhesion of germ tubes or yeast to freshly collected human BECs
Biofilm formation		Decreased biofilm formation on serum-coated polystyrene	No difference on serum-coated polystyrene

Blank cells in the table indicate a parameter that was not reported.

*All three of the newly constructed Δpir mutant strains were listed in the same column of the table because results were similar for each.

**Figure 1:**
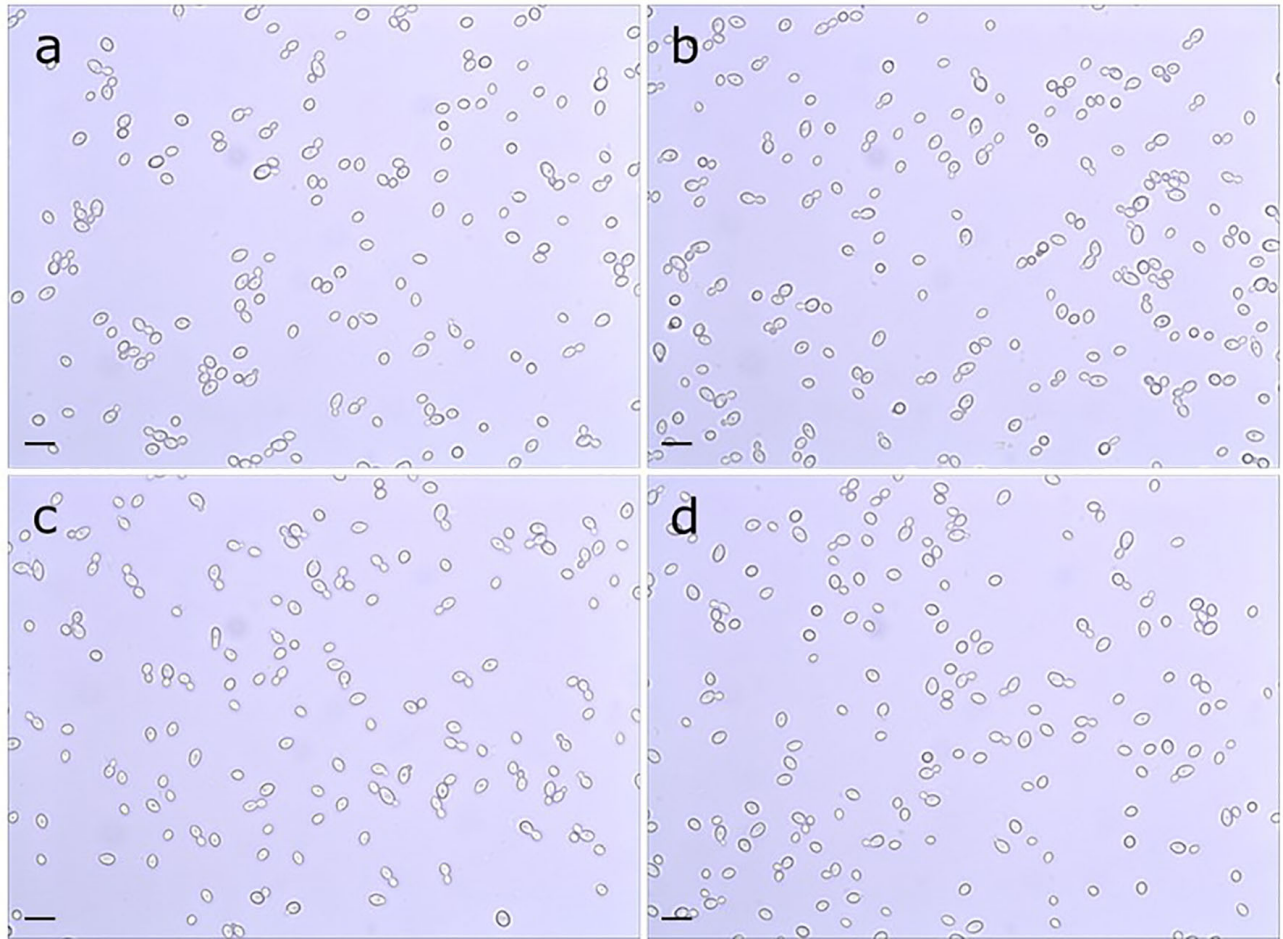
Light micrograph of *C. albicans* cells from parent strain **(A)** SC5314, **(B)** Δ*pir1-1*/Δ*pir1-2* strain 3097, **(C)** Δ*pir32-1*/Δ*pir32-2* strain 3545 and **(D)** Δ*pir1-1*/Δ*pir1-2* Δ*pir32-1*/Δ*pir32-2* strain 3543. Cultures were grown for 16 h in PDB at 37°C and 200 rpm shaking. All strains grew as budding yeast with minimal presence of elongated/irregular forms or aggregation. The scale bar in the lower left of each image = 10 μm.

**Figure 2:**
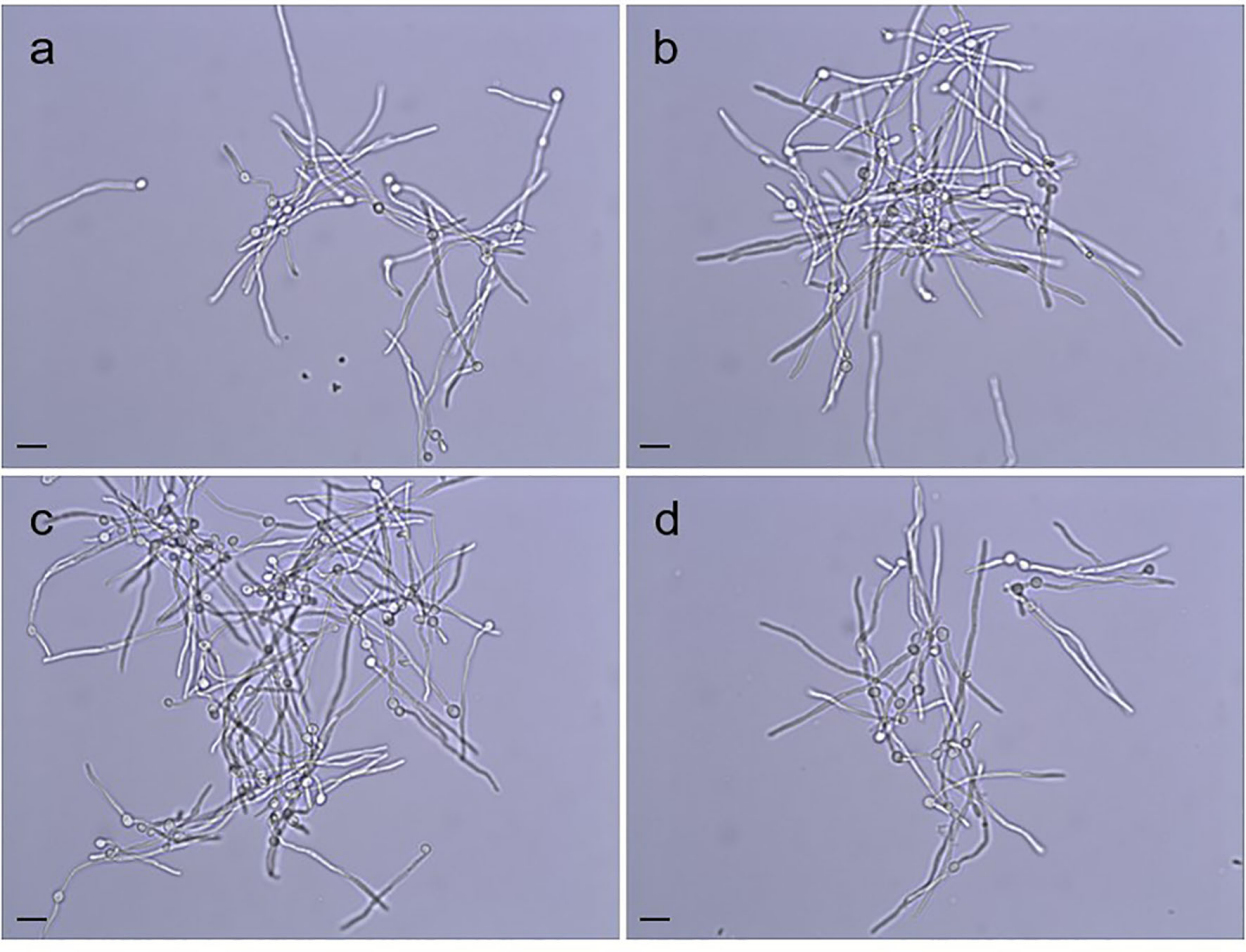
Hypha morphology of the strains **(A)** SC5314, **(B)** 3097 (Δ*pir1-1*/Δ*pir1-2*), **(C)** 3545 (Δ*pir32-1*/Δ*pir32-*2) and **(D)** 3543 (Δ*pir1-1*/Δ*pir1-2* Δ*pir32-1*/Δ*pir32-2*) incubated in FBS. Aliquots were collected from each culture at 2 h, 4 h, 6 h, 8 h and 10 h, while incubating at 28°C or 37°C. Cells shown above were paraformaldehyde-fixed after incubation at 37°C for 6 h. No difference in the shape, length, or clumping of the hyphae was noted among the strains for any of the growth conditions. The scale bar in the lower left of each image = 10 μm.

Doubling times were calculated for *C. albicans* SC5314 (2.02 ± 0.05 h), 3097 (2.02 ± 0.05 h), 3545 (2.11 ± 0.05 h), and 3543 (1.97 ± 0.05 h) in yeast extract/peptone/dextrose (YPD) medium. There was no significant difference in growth rate among the strains (P = 0.07). Examination of cellular morphology showed single or budding yeast with minimal aggregation or elongated/irregular forms (**Figure 1**). Classic round, white colonies appeared on YPD agar following streaking and incubation of the plates at 37°C for 24 h (**Supplementary Figure S1**). The consistent colony size among the various strains further reinforced the conclusion of their similar growth rates. Prolonged incubation of colonies on potato dextrose agar (PDA) plates (2 weeks at 28°C) also showed similar colony morphologies among the strains (**Supplementary Figure S2**). The outermost fringed edge that emerged from the colonies was consistent with hypha growth observed in wild-type *C. albicans* isolates. Germ tube formation was assessed by counting the number of germ-tube-positive cells in culture flask samples at various time points (**Table 2**). The overall effect of strain was not significant in the statistical analysis although a limited number of strain/growth medium combinations showed more-rapid germ tube formation for a mutant strain compared to the parent. Cellular morphology of hyphae was similar for all four strains in all growth conditions tested (**Figure 2**).

**Table 2:** Percent germ tubes in cultures of control strain SC5314 and the *Δpir/Δpir* mutants.

Strain*	Growth Condition				
	RPMI 60 min	Spider medium 60 min	YPD + serum 60 min	PDB + serum 40 min	PDB + serum 60 min
SC5314	33.8 ± 3.4	22.8 ± 6.6	11.7 ± 6.7	16.3 ± 6.8	62.5 ± 4.4
3097	37.2 ± 3.4	28.8 ± 6.6	11.3 ± 6.7	19.8 ± 6.8	69.7 ± 4.4
3545	33.8 ± 3.4	36.0 ± 6.6^†^	9.3 ± 6.7	22.8 ± 6.8	69.5 ± 4.4
3543	38.2 ± 3.4	37.3 ± 6.6^†^	9.7 ± 6.7	30.2 ± 6.8	76.8 ± 4.4^†^

*Strains include the wild-type control (SC5314), Δpir1-1/Δpir1-2 (3097), Δpir32-1/Δpir32-2 (3545) and Δpir1- 1/Δpir1-2 Δpir32-1/Δpir32-2 (3543). Replicate observations were collected for independent cultures on at least three different days. Means and their standard errors are shown. There was no significant strain effect for any of the growth conditions. P = 0.73 for RPMI, 60 min. P = 0.09 for Spider medium, 60 min. P = 0.95 for YPD + serum, 60 min. P = 0.24 for PDB + serum, 40 min. P = 0.13 for PDB + serum, 60 min.

^†^Individual comparisons showed that strain 3543 formed germ tubes more quickly than the control (SC5314) in Spider medium (P = 0.03) and at 60 min in potato dextrose broth (PDB) with serum (P = 0.03). Strain 3545 also formed germ tubes more quickly than the control in Spider medium (P = 0.04).

Strains 3097, 3545 and 3543 were compared to the SC5314 parent for their ability to grow under various stress conditions and in the presence of compounds that may reveal defects in cell integrity. No differences were observed between control and mutant strains for growth on 1 M or 1.5 M sodium chloride (**Supplementary Figure S3**), in response to oxidative stress (**Supplementary Figure S4**), following heat shock (**Supplementary Figure S5**), or in the presence of SDS (**Supplementary Figure S6**), cystamine (**Supplementary Figure S7**), or Hygromycin B (**Supplementary Figure S8**).

**Figure 3:**
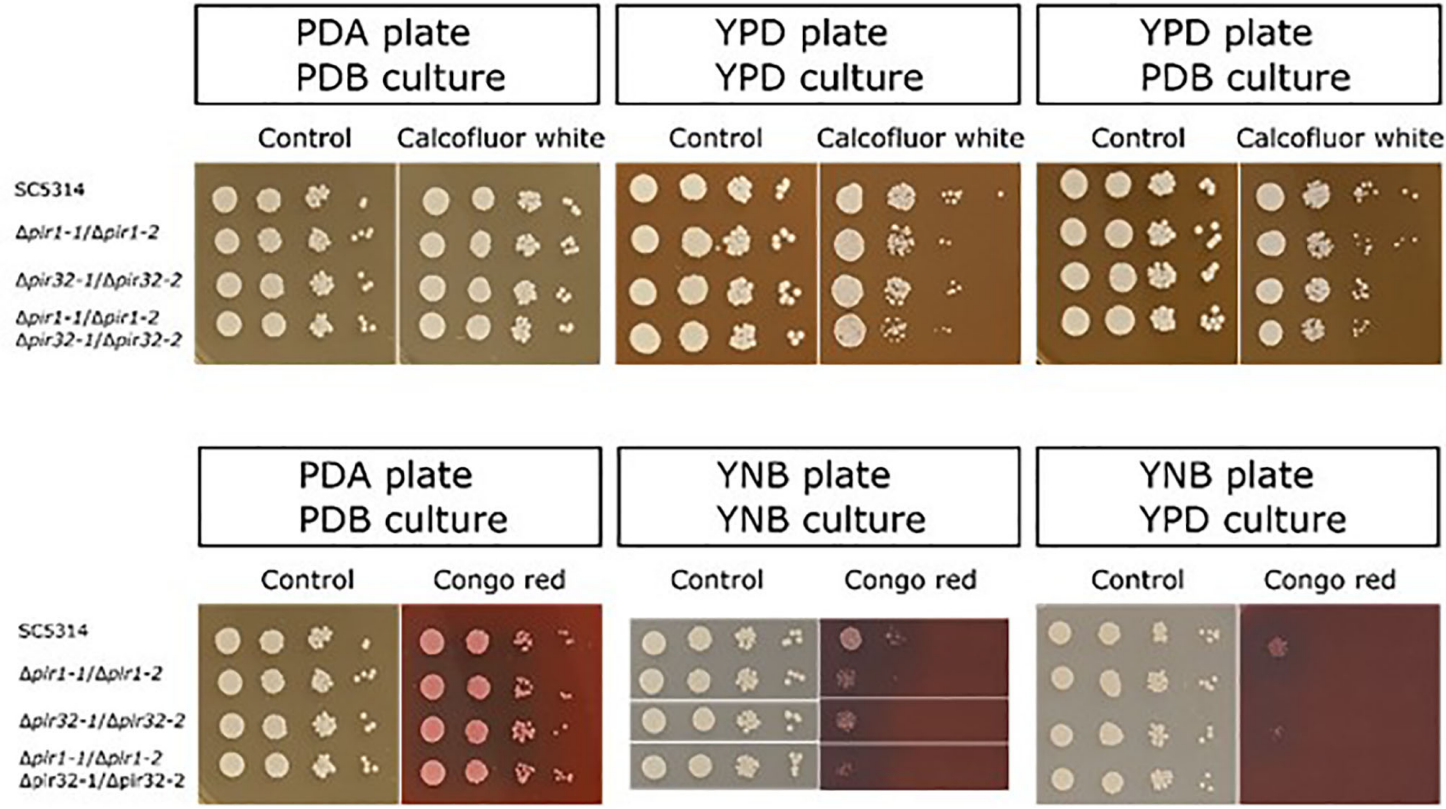
(Top panel) Growth of the *C. albicans Δpir* mutant strains on agar plates containing calcofluor white. Starter cultures were grown in either YPD or PDB. Cells were washed in DPBS, counted, and serially diluted. Five μl of each dilution (10^6^ to 10^3^ cells/ml, left to right in each panel) were spotted onto a YPD or PDA plate containing 150 μg/ml calcofluor white. Plates were incubated at 37°C for 48 h and photographed. Cells were grown without calcofluor white as a control (left panel for each pair of images). Growth inhibition by calcofluor white was dependent on the combination of growth medium used for the starter culture and for the agar plate. (Lower panel) Growth of the *Δpir* mutant strains on agar plates containing Congo red. Starter cultures were grown in PDB, YNB, or YPD. Cells were washed in DPBS, counted, and serially diluted. Five μl of each dilution (10^6^ to 10^3^ cells/ml, left to right in each panel) were spotted onto a PDA or YNB plate containing either 50 μg/ml (left panel) or 30 μg/ml (middle and right panels) Congo red. Plates were incubated at 37°C for 48 h and photographed. Cells were grown without Congo red as a control (left panel for each pair of images). Growth inhibition by Congo red was dependent on the culture medium and observed only for cells grown on YNB plates. Results indicating Congo red sensitivity among the mutant strains were not obtained in a repeatable manner. In each panel, control and experimental plates pictured were from the same assay day.

Growth of the mutant and parent strains on agar plates containing calcofluor white or Congo red (**Figure 3**) showed limited phenotypic effects of deleting *PIR* genes. Results were dependent on the combination of growth medium for the starter culture and agar plate. For example, no effect of calcofluor white was observed for parent or mutant strains grown in potato dextrose broth (PDB) then spotted onto a potato dextrose agar (PDA) plate (**Figure 3**). There was a slight effect of gene mutation on growth when cells were cultured in YPD or PBD liquid and spotted onto a YPD plate. Differential sensitivity to Congo red was observed inconsistently among assay replicates. When noted, a modest effect was apparent for cells grown on yeast nitrogen broth (YNB) agar plates (**Figure 3**).

Strain SC5314 and the *Δpir/Δpir* strains were tested for their sensitivity to antifungal drugs using the Sensititre method (**Supplementary Table S1**). Results were nearly identical among strains for the antifungal drugs tested. A one- or two-dilution difference was observed between the parent strain and the *Δpir* mutants with the mutant isolates showing increased resistance to anidulafungin. Mutant strains showed a one dilution increased sensitivity to itraconazole compared to the parent strain.

The *Δpir* strains were compared to the control for their ability to form a biofilm on the serum-coated surface of a polystyrene tissue culture plate. Crystal violet absorption was used as a measure of biofilm formation. The optical density values obtained were 0.44 ± 0.02 for strain SC5314, 0.46 ± 0.02 for strain 3097, and 0.45 ± 0.02 for strains 3545 and 3543 (P = 0.69) suggesting no difference in biofilm formation among the strains.

*C. albicans Δpir* strains were analyzed for their adhesion to freshly collected human buccal epithelial cells (BECs). Both germ tubes and yeast forms were tested. Germ tube adhesion was described as the mean number of germ tubes per BEC. Values were 22.9 ± 3.8 (SC5314), 20.9 ± 3.8 (3097), 28.2 ± 3.8 (3545), and 24.3 ± 3.8 (3543). There was no significant difference between the means (P = 0.33), suggesting that deletion of *PIR1* and/or *PIR32* did not affect the adhesive qualities of the *C. albicans* germ tube surface. Yeast cell adhesion was described as the total number of yeast cells that adhered to 100 BECs. Values were 31.2 ± 11.8 (SC5314), 65.8 ± 11.8 (3097), 67.8 ± 11.8 (3545), and 49.7 ± 11.8 (3543). Although there was a trend toward increased yeast cell adhesion in the mutant strains, the effect was not significant (P = 0.20).

### Revealing the Larger *PIR* Family

**Figure 4:**
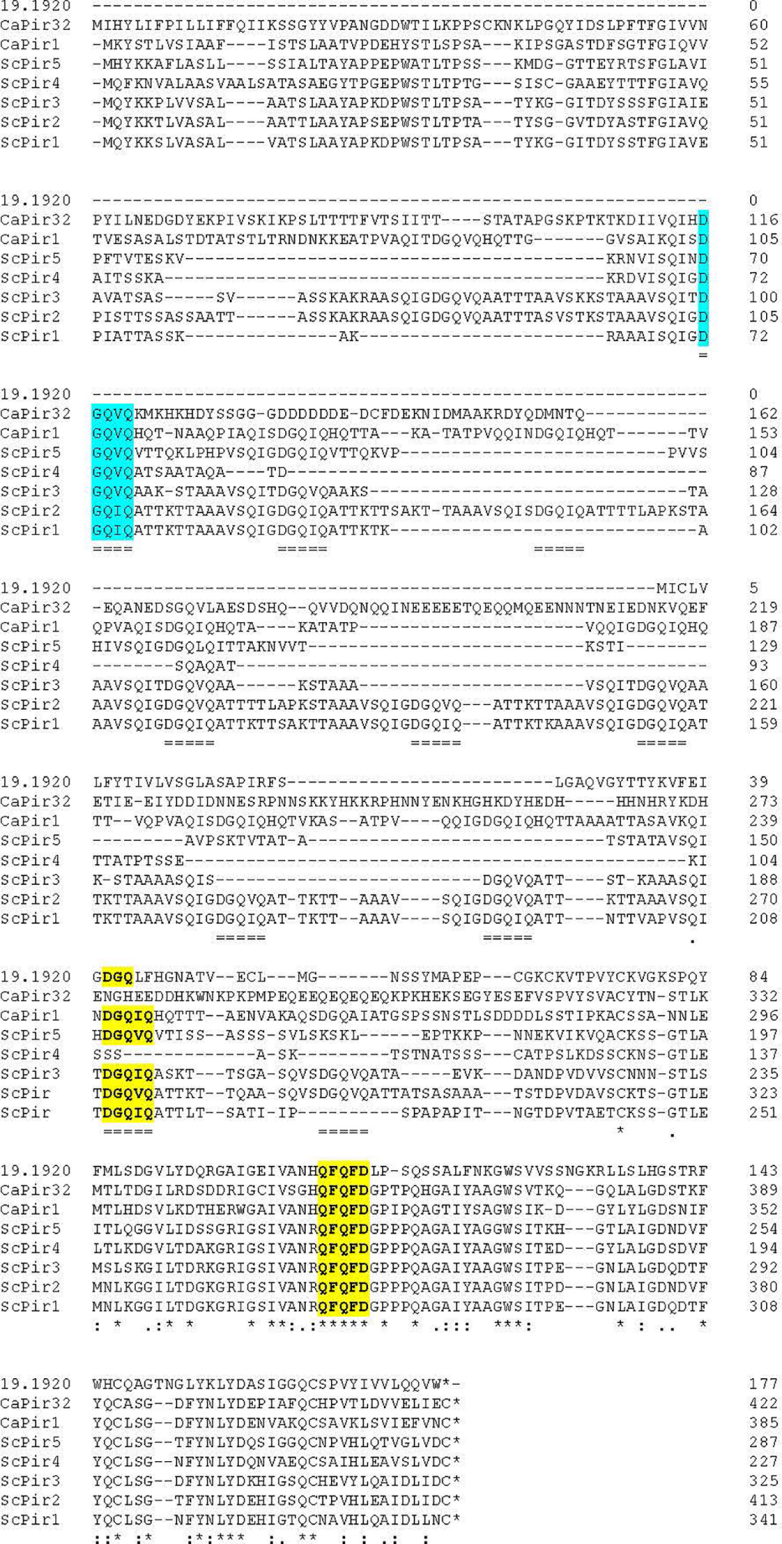
Alignment of amino acid sequences from known *C. albicans* and *S. cerevisiae* Pir proteins with the sequence of the newly identified *C. albicans* Pir protein, orf19.1920. Sequence position within each protein was noted by the numbers on the right of the diagram. Alignment among DGQ[V/I]Q repeated sequences was marked with double underlining. Asterisks showed positions of identity among all sequences. The overall alignment showed that the conserved Pir repeat was most abundant in ScPir1, ScPir2, and CaPir1. Despite its highly divergent sequence, the conserved Pir repeat was also present in CaPir32 (blue highlight). The newly recognized orf19.1920 was considerably shorter than the other proteins and had one Pir consensus sequence, shortened to DGQ (yellow highlight). Conservation among all sequences was most evident toward the C-terminal end of the proteins leading to identification of additional shared sequence motifs such as QFQFD (yellow highlight).

The unexpected lack of phenotypic consequences for deleting *C. albicans PIR1* and/or *PIR32* suggested the possibility that *C. albicans* had other proteins with Pir function. Initial searches of the *C. albicans* genome suggested that the protein predicted from orf19.1920 was similar to *C. albicans* Pir1 and Pir32 even though it was much smaller (177 amino acids, compared to 384 and 422 amino acids, respectively). The DGQ motif, a portion of the Pir repeat unit, was predicted from orf19.1920. C-terminal-region amino acid sequences were conserved among the proteins suggesting additional conserved motifs such as QFQFD (**Figure 4**).

Further BLAST searches of the *C. albicans* genome using the *S. cerevisiae* and *C. albicans* Pir sequences as queries revealed additional potential Pir proteins. Besides orf19.1920, eight other proteins consistently were identified: orf19.31, orf19.4515, orf19.1148, orf19.555, orf19.654, orf19.4463, orf19.4170, and orf19.3512. Alignment of these sequences (**Supplementary Figure S9**) showed the C-terminal region similarities that were observed in **Figure 4**, including the conserved QFQFD motif. Each of the newly identified proteins also had the DGQ motif found in the other Pir proteins (**Supplementary Figure S9**).

The nine *C. albicans* genes identified by BLAST were annotated with a “*CIS3–*” designation in the *Candida* Genome Database (CGD; http://www.candidagenome.org; Skrzypek et al., 2017; **Supplementary Table S2**). In *S. cerevisiae*, *CIS3* (*cik1*Δ suppressing) is an alias for *PIR4* (Manning et al., 1997). *PIR4* was the best *S. cerevisiae* BLAST “hit” for *C. albicans* orf19.1148, although most of the *C. albicans* “*CIS3–*” sequences aligned best with *S. cerevisiae PIR3*. The *C. albicans* genes were located on 4 of the 8 C*. albicans* chromosomes. Orf19.555 and orf19.654 translated to predict essentially identical proteins. These genes were located on Chromosome R, approximately 42 kb apart and transcribed in opposite directions. CGD annotations recognized the proteins for their potential role in cell wall structure. Orf19.555 and orf19.4515 were proposed to be essential since previous large-scale mutant strain construction attempts failed to produce null mutants for these genes. Two of the genes (orf19.4170 and orf19.3512) were noted for increased expression during chlamydospore development and given the names *CSP2* and *CSP1*, respectively (Palige et al., 2013). Examination of the Palige et al. (2013) dataset indicated that 5 of the 9 C*. albicans* genes and their *C. dubliniensis* orthologs were up-regulated under growth conditions that favor chlamydospore formation (**Supplementary Table S2**).

*S. cerevisiae* and *C. albicans* Pir sequences were used as BLAST queries to identify potential Pir proteins in other fungal species including *Candida dubliniensis* CD36 (10 Pir proteins), *Candida tropicalis* MYA-3404 (6), *Lodderomyces elongisporus* NRRL YB-4239 (2), *Candida parapsilosis* CDC 317 (6), *Candida orthopsilosis* Co 90-125 (4), *Candida metapsilosis* ATCC 96143 (7), *Candida auris* B8441 (3), *Clavispora lusitaniae* ATCC 42720 (2), *Yamadazyma tenuis* ATCC 10573 (3), *Spathaspora passalidarum* NRRL Y-27907 (3), *Scheffersomyces stipitis* CBS 6054 (3), *Meyerozyma guilliermondii* ATCC 6260 (3), *Debaryomyces hansenii* CBS 767 (2), and *Candida glabrata* CBS 138 (5). Species studied included human pathogens and some with biotechnological importance, many of which are featured on the *Candida* Gene Order Browser (CGOB; Maguire et al., 2013). The predicted Pir proteins and their features are presented in **Supplementary Table S3**. Pattern search functions for the DGQ and QFQFD motifs were used within CGD, SGD, and FungiDB (https://fungidb.org) to seek additional potential Pir proteins. The DGQIQ sequence was found in other *S. cerevisiae* cell wall proteins (Cwp1, Cwp2, Tir1, Tir2), but none also had QFQFD (data not shown). No additional proteins were identified to add to the Pir family proposed here.

The predicted Pir proteins each had a secretory signal peptide although current genome sequences and annotations sometimes misassigned the protein start (**Supplementary Table S3**). In cases where a signal peptide was not apparent, one was located by selecting a nearby Met as the protein start. Examples included CaCis308, Cd15040, Cd30400, Cp205800, Co0D05920, and Cl05291. For these entries, the original annotated protein sequence was included in full, with “strikethrough” used to indicate amino acids unlikely to be part of the protein. Signal peptide sequences were highlighted in gray. Light blue highlights were used for DGQ sequences and yellow for QFQFD. Each protein had both motifs except *C. glabrata* CAGL0M08514g which lacked DGQ. Some motifs were modified (e.g. EGQ instead of DGQ, QLQFD instead of QFQFD) and marked accordingly.

CGOB (Maguire et al., 2013), which displays pillars of proteins aligned due to syntenic location (i.e. conserved blocks of gene order between species) in the various genome sequences, was used to assign the genes/proteins to ortholog groups (OG) named with an arbitrary alphabetic code. For example, orthologs of *C. albicans PIR1* were assigned to Group A, *C. albicans PIR32* orthologs to Group B, etc. moving down **Supplementary Table S3** in order of gene/protein presentation. Because *C. glabrata* was not included in CGOB, a Group A ortholog was inferred from annotation of the ATCC 2001 genome sequence (Xu et al., 2020). **Supplementary Figure S10** expands upon the relationship between *S. cerevisiae* and *C. glabrata PIR* loci, which are contiguous on two chromosomes in each species. Each species in **Supplementary Table S3** except *S. passalidarum* had a Group A (*CaPIR1, ScPIR1*) ortholog. These sequences featured multiple copies of the DGQ motif, consistent with the presence of multiple repeat units. A Group B (*CaPIR32*) ortholog was found in most species, except *C. lusitaniae* and *D. hansenii* which had a Group M ortholog instead. Group M orthologs were also present in *C. auris*, *Y. tenuis*, *S. passalidarum*, *S. stipitis*, and *M. guilliermondii*.

Known features of *S. cerevisiae* Pir proteins were also assessed for the proteins in **Supplementary Table S3**. *S. cerevisiae* Pir proteins are processed by Kex2 at a site located between the end of the secretory signal peptide and the DGQ motif (Mrša et al., 1997). These sites were marked in green for the *S. cerevisiae* Pir proteins and predicted for the other proteins using ProP 1.0 (Duckert et al., 2004). Additional potential processing sites not recognized by the program were highlighted in purple. *S. cerevisiae* Pir proteins are also O-mannosylated (Mrša et al., 1997). NetOGlyc – 4.0 (Steentoft et al., 2013) was used to predict O-glycosylation sites in the newly identified proteins (**Supplementary Table S3**). *S. cerevisiae* and *C. glabrata* proteins were predicted to be heavily O-glycosylated in contrast to the 10 C*. dubliniensis* Pir proteins that were predicted to have no O-linked carbohydrate. Sequences were also assessed for N-linked carbohydrate addition potential using NetNGly – 1.0 (Gupta and Brunak, 2002). A limited number of sites were identified and highlighted in red.

**Figure 5:**
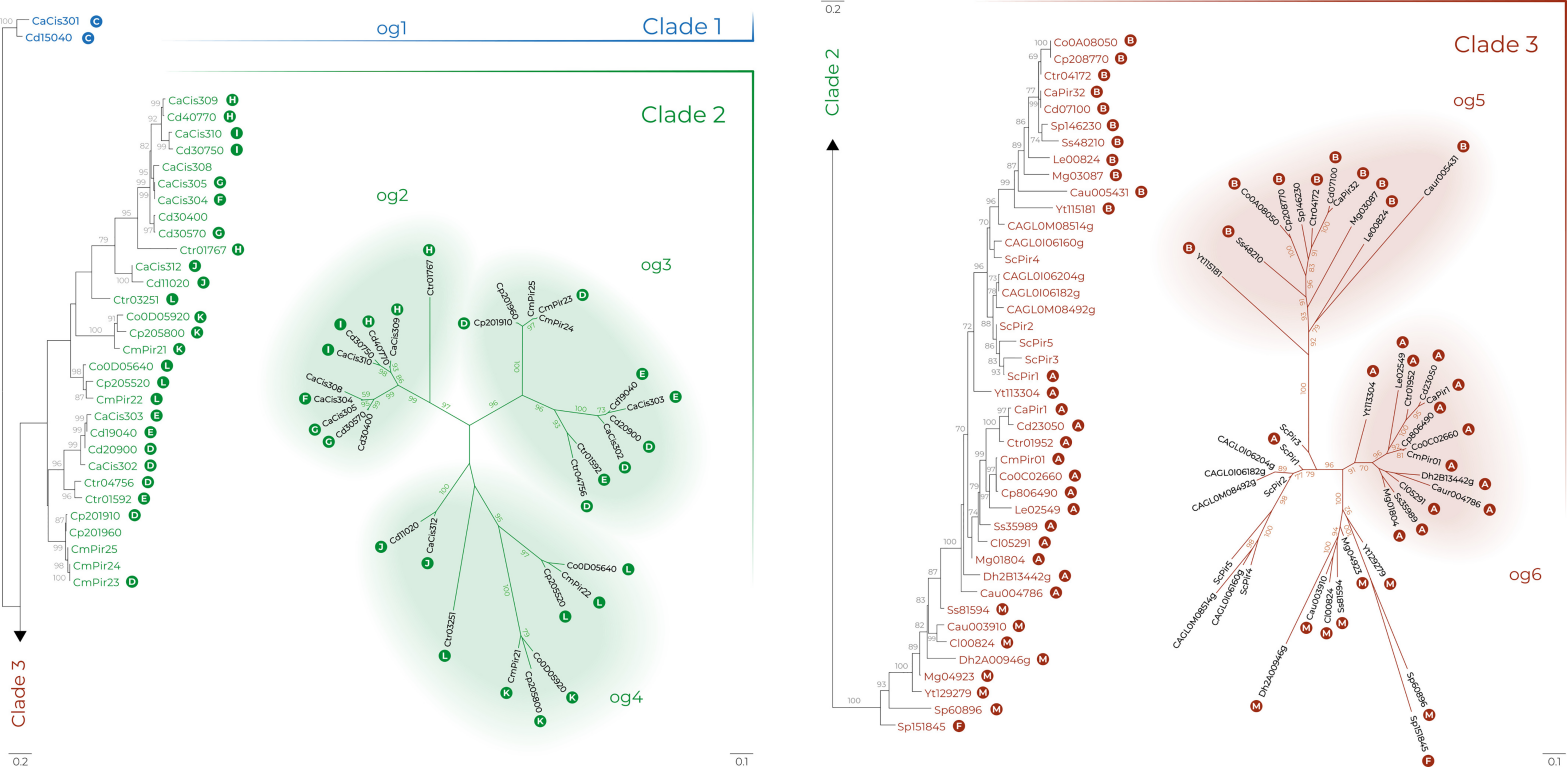
Pir family phylogeny in *Candida albicans* (Ca), *C. auris* (Cau), *C. dubliniensis* (Cd), *Candida glabrata* (CAGL), *C. metapsilosis* (Cm), *C. orthopsilosis* (Co), *C. parapsilosis* (Cp), *C. tropicalis* (Ctr), *Clavispora lusitaniae* (Cl), *Debaryomyes hansenii* (Dh), *Loddermyces elongisporus* (Le), *Meyerozyma guilliermondii* (Mg), *Saccharomyces cerevisiae* (Sc), *Scheffersomyces stipitis* (Ss), *Spathaspora passalidarum* (Sp) and *Yamadazyma tenuis* (Yt). The best-scoring maximum likelihood tree is shown with maximum likelihood bootstrap values appended to the nodes; only support values higher than 70% were shown. Trees in radial layout (right) show phylogenies of clades 2 and 3 with tentative orthologous groups (og) designated by a colored background. White letters in colored circles indicate *Candida* Gene Order Browser Ortholog Groups (OG) as designated in **Supplementary Table S3**. Scale bars represent substitutions per site with different scale bars presented for each tree layout (left and right).

Amino acid sequences from **Supplementary Table S3** were aligned and the conserved positions used to estimate the Pir protein phylogeny, displayed in **Figure 5** as a maximum likelihood tree. Pir protein sequences were highly divergent and resolved into three main clades. Clade 1 only included the orthologs CaCis301 and CdCis15040. Phylogeny conclusions were supported by examination of CGOB that indicated synteny for the *CaCIS301* and *CdCIS15040* loci (Group C; **Supplementary Table S3**). CGOB designations were included across **Figure 5** to further highlight similar observations from analysis of synteny and the maximum likelihood tree.

Clade 2 included only proteins from the closely related species *C. albicans* (Ca), *C. dubliniensis* (Cd), *C. tropicalis* (Ctr), *C. parapsilosis* (Cp), *C. orthopsilosis* (Co), and *C. metapsilosis* (Cm). Proteins were segregated into three provisional orthologous groups (og2 to og 4, **Figure 5**). In og2, the ancestral protein Ctr01767 underwent multiple gene duplications in *C. albicans* and *C. dubliniensis*. These genes were found to be up-regulated during chlamydospore formation in *C. albicans* and *C. dubliniensis* (**Supplementary Table S2**).

In contrast to og2, the origin of og3 was likely preceded by gene duplication because all proteins included two in-paralogs except for *C. metapsilosis* with three in-paralogs (CmPir23, CmPir24, CmPir25). This ortholog seemed to have been completely lost from the *C. orthopsilosis* genome. The og4 group included proteins from CGOB Groups J, K, and L (**Supplementary Table S3**). These proteins were similar in that they did not encode any of the conserved Cys residues that were speculated to attach Pir proteins to the *S. cerevisiae* cell wall (Castillo et al., 2003; see below). Synteny analysis placed Ctr03251 as an ortholog of Co0D05640/Cp205520/CmPir22.

Clade 3 included two nearly completely resolved orthologous groups without any in-paralogs: og5 (11 proteins; CGOB Group B that includes CaPir32) and og6 (13 proteins; Group A that includes CaPir1). Clade 3 also included the *S. cerevisiae* and *C. glabrata* proteins that clustered together. Synteny analysis suggested that ScPir1 and Yt113304 were CaPir1 orthologs (**Supplementary Table S3**).

Some proteins in clade 3 did not resolve into clear orthologous groups. Combining evidence from the phylogenetic analysis with CGOB synteny data suggested that Sp151845 was an ortholog of CaCis304 (Group F), located in Clade 2, og2. Other examples of unresolved Clade 3 proteins included proteins designated as Group M (**Supplementary Table S3**). Like proteins in Group A, Group M sequences contained multiple copies of the DGQ motif. Overall, data from the phylogenetic analysis showed strong agreement with synteny data from CGOB and led to greater understanding of the relationship between *PIR* genes in these species.

### *PIR* Repeated Sequences and Allelic Variation

Attention paid to the *S. cerevisiae PIR* repeated sequences was so great that the genes were named for this feature (Toh-E et al., 1993). Repeat-dependent allele length affects function of other fungal proteins such as those in the agglutinin-like sequence (Als) family (Oh et al., 2005; Hoyer et al., 2008). Characterization of *PIR* allelic variation was pursued in *C. albicans* using a previously described collection of diverse strains isolated from humans and wildlife species (Wrobel et al., 2008).

**Figure 6:**
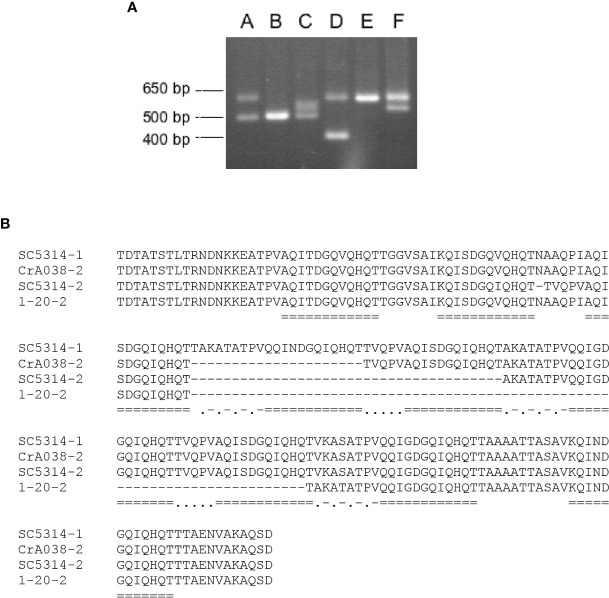
Variation among *C. albicans PIR1* alleles attributed to the repeat-containing region. **(A)** Six different patterns of PCR products amplified using primer pair 3/4 (**Supplementary Figure S12**) and genomic DNA from a diverse collection of *C. albicans* isolates (**Supplementary Table S4**). Patterns were labeled A through F, corresponding to DNA amplified from strains SC5314, 1-178, 1-233, 1-20, OpA052, and CrA038, respectively. Molecular size markers are on the left of the image. **(B)** Alignment of the Pir1 amino acid sequences translated from the four alleles shown in **(A)**. *PIR1* alleles from SC5314 had 9 and 7 copies of the repeated sequence (called SC5314-1 and SC5314-2, respectively). The smaller *PIR1* allele from strain CrA038 (CrA038-2) had 8 repeat copies and the smaller allele from strain 20-2 (20-2-2) had 5 copies. Repeated sequences were marked by double underlining. Dots and dashes marked a conserved intervening sequence ([A/V] KA [S/T] ATPV). Dots alone marked a different, conserved intervening sequence (TVQPV).

CGD annotation suggested that *PIR1* alleles were different lengths in *C. albicans* strain SC5314 (**Supplementary Figure S11**). Three primer sets were designed to amplify different regions of *PIR1* (**Supplementary Figure S12**). Length variation was found in the center of the gene and attributable to variable numbers of repeated sequence copies. PCR amplification of genomic DNA from 41 human and 27 wildlife *C. albicans* isolates revealed six different PCR product patterns (**Figure 6A**; **Supplementary Table S4**).

DNA sequence analysis of the cloned fragments showed identity among the large fragments from patterns A, D, E and F. Sequences of the smaller fragment from patterns A and C were identical to each other and to the fragment from B. The larger fragment from pattern C was identical to the smaller fragment from F. In all, four distinct alleles were detected which predicted 9, 8, 7 or 5 copies of the 12-amino-acid repeated sequence [A/Q/K]QI[T/N]DGQ[V/I]QHQT (**Figure 6B**).

PCR product patterns were strongly associated with phylogenetic clade of the *C. albicans* isolate (Odds et al., 2007). Twenty-nine of the clade 1 isolates (94%) had PCR product pattern A; only 2 strains showed a different pattern, in this case, pattern E which resulted from loss of heterozygosity in pattern A. In both human and wildlife *C. albicans* isolates, PCR product patterns B and C were associated with clade 3 strains (**Supplementary Table S4**). Pattern B reflected loss of heterozygosity in pattern C (**Figure 6**). Clade association with PCR product pattern was inconsistent between human and wildlife *C. albicans* isolates in clades 8 and 11. In human isolates, clade 8 strains tended to have PCR product pattern D while wildlife isolates had pattern E (which results from loss of heterozygosity in pattern D).

The SC5314 *PIR32* locus had far less allelic variation than *PIR1* (**Supplementary Figure S13**). In *C. albicans* SC5314, *PIR32* encoded only one Pir repeat copy and it was truncated to 9 amino acids. Length variation between SC5314 *PIR32* alleles was limited to only 6 nucleotides (2 amino acids). PCR analysis (**Supplementary Figure S14**) echoed this lack of length variation in the collection of human and wildlife strains. DNA sequencing of the cloned PCR product from 10 selected strains showed limited sequence differences including I58V, L64P, and P100S (**Supplementary Figure S13**). Alleles from the isolates also showed some of the variability observed between SC5314 alleles such as expansion of sequences encoding tracts of the same amino acid (D or E, for example) or the KV-to-NA change observed near amino acid 215.

This detailed analysis suggested the hypothesis that Group A genes (orthologs of *CaPIR1*) have limited length variation in repeat copy number while Group B genes (orthologs of *CaPIR32*) have even-more-subtle allelic variation. These ideas were tested by examining draft genome sequences in the NCBI database (https://www.ncbi.nlm.nih.gov/genome). The short length of the *PIR* repeat unit and overall short length of *PIR* genes provided reasonable assurance of accurate allele assembly in the various draft genome sequences. Examination of several dozen *S. cerevisiae* genome sequences failed to reveal repeat copy number variation for any of the 5 *PIR* genes (data not shown). Among *C. auris* genome sequences, *Cau004786* (Group A) and *Cau003910* (Group M) alleles with one more or fewer repeat unit copy were observed (data not shown). Overall, the most-common *PIR* allelic variations were subtle sequence changes similar to those observed for *CaPIR32* (above).

Repeat unit sequences were aligned across Pir proteins to attempt to derive a consensus sequence. Repeat units varied in sequence and length within the same protein, as well as across Pir proteins from the various species (data not shown). For example, ScPir2 repeat units varied from 18 to 26 amino acids in length and were different in sequence from the 17- to 25-amino-acid-long repeat units in Cl00824. Each repeated unit tended to include the motif QI_DGQ_Q for the species studied here. For proteins that contained only one repeat copy, the consensus sequence was further reduced to DGQ. The DGQ sequence was found in all proteins examined except CAGL0M08514g (**Supplementary Table S3**).

### Pir Protein Structural Predictions

ScPir4 was chosen for much of the biochemical characterization of Pir proteins because it has only one copy of the repeated unit (Castillo et al., 2003; Ecker et al., 2006). Various techniques were used to assign function to specific amino acids and protein regions, particularly with respect to Pir localization and cell-wall linkage. Although an experimental structural solution was never derived for a Pir protein, recent release of the highly accurate structural prediction algorithm AlphaFold (Jumper et al., 2021) led to online availability of structural predictions for the entire *S. cerevisiae* and *C. albicans* proteome (https://www.alphafold.ebi.ac.uk). The AlphaFold Colab sites (see *Materials and Methods*) were also used to make structural predictions for the other Pir proteins (**Supplementary Table S3**). These predictions were based on amino acid sequences and did not take protein modifications such as glycosylation into account. ScPir4 was used as a starting point to display structural features inferred from the literature and from data presented above, and to explore feature conservation across the larger Pir family.

**Figure 7:**
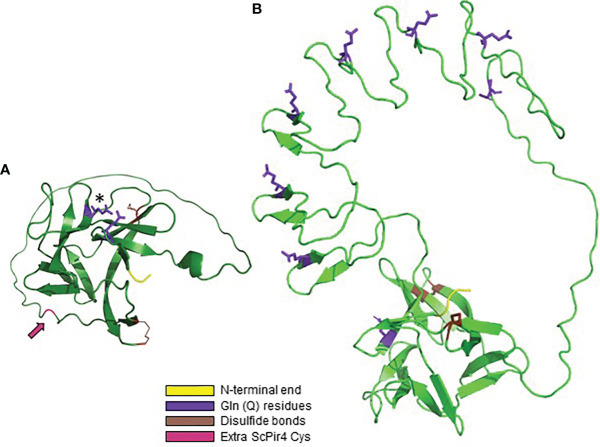
AlphaFold structural predictions for *S. cerevisiae* Pir proteins. **(A)** ScPir4, processed to remove the secretory signal peptide and to reflect Kex2 cleavage. The mature protein began at amino acid 65; the N-terminal sequence of the mature protein (DVI) is highlighted in yellow. The Gln (Q74) residue that forms a carboxyl ester with β-1,3-glucose is highlighted in purple and marked with an asterisk. Also highlighted in purple is Q160, the N-terminal Gln in the QFQFD motif. ScPir4 Cys residues were predicted to form two disulfide bonds (C195-C212, C128-C225) highlighted in brown. These Cys residues are conserved in all *S. cerevisiae* Pir proteins. ScPir4 has 6 Cys residues: one (C41) removed by Kex2 processing and the other (C120; highlighted in magenta and indicated by an arrow) available to form disulfide bonds with other components of the cell wall, likely explaining release of ScPir4 by β-mercaptoethanol treatment (Castillo et al., 2003). **(B)** AlphaFold structural prediction for ScPir1 to show the position of repeated units relative to the core region of antiparallel beta-sheets. The start of the mature protein (AAA, highlighted in yellow) reflected removal of the secretory signal peptide and Kex2 cleavage. The Gln from each DGQ in the repeated unit was highlighted in purple. Gln from the first repeated unit (Q74) was adjacent to the N-terminal Gln of the QFQFD motif (the latter was not highlighted). The four conserved Cys in ScPir1 were predicted to form two disulfide bonds (highlighted in brown).

**Figure 7A** shows the AlphaFold predicted structure for ScPir4, the smallest of the ScPir proteins. **Figure 7B** contrasts that image with ScPir1 that includes 8 copies of the Pir repeated sequence. Both molecules had a core region of antiparallel beta-sheets. Ecker et al. (2006) elegantly demonstrated that ScPir4 Q74 forms a carboxyl ester with β-1,3-linked glucose. Numerous repeat copies in ScPir1 presumably provide more opportunity for linkages to cell wall β-glucan. Identification of the larger Pir family revealed the conserved QFQFD motif (**Figure 5**). In the predicted ScPir4 structure (**Figure 7A**), the N-terminal Q in the QFQFD motif was in close approximation to Q74 (of DGQ). The first DGQ repeat in ScPir1 (**Figure 7B**) was predicted to occupy the same location as the single repeat unit in ScPir4. Structural predictions for other Pir proteins with multiple repeat units often, but not always, showed the first repeat unit in this location (data not shown).

The location of Cys residues in *S. cerevisiae* Pir proteins was examined because treatment with β-mercaptoethanol was noted to release ScPir4 from the cell wall (Castillo et al., 2003). Each *S. cerevisiae* Pir protein has 4 conserved Cys residues while ScPir4 has two more for a total of six (**Figure 7**). AlphaFold predicted formation of two disulfide bonds among the 4 Cys residues, which included a C-terminal Cys that is conserved among the *S. cerevisiae* Pir proteins. For ScPir4, one of the extra Cys (C41) was removed by Kex2 processing, while location of the other (C120) was predicted in a loop with the potential to form a disulfide bond with other cell wall structural constituents (**Figure 7A**).

**Figure 8:**
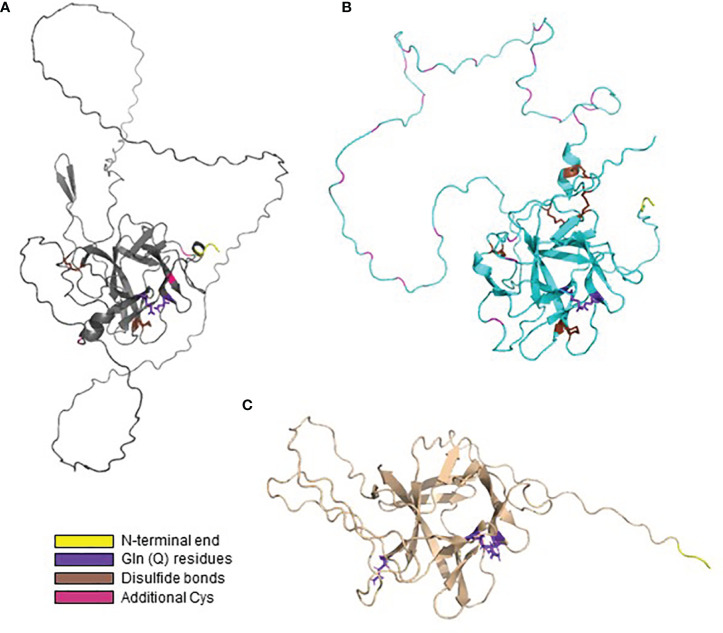
AlphaFold structural predictions for representative proteins that vary from the *S. cerevisiae* model protein sequences. In each image, Gln residues are highlighted in purple, Cys in predicted disulfide bonds marked in brown and other Cys highlighted in magenta. N-terminal amino acids are highlighted in yellow. **(A)** AlphaFold predicted structure for Co0A08050, representative of CGOB Group B sequences. **(B)** Predicted structure for the Cys-rich CaCis310 (Csp1). **(C)** Predicted structure for CmPir21, which did not have any Cys residues.

AlphaFold was also used to predict the structures for Pir proteins with sequences that varied considerably from the *S. cerevisiae* models that were the focus of published biochemical characterization. For example, proteins in CGOB Group B tended to be larger than the *S. cerevisiae* model proteins, had few copies of the Pir repeat unit, and unusual amino acid compositions. **Figure 8A** shows the AlphaFold predicted structure for Co0A08050 (7 Cys with 4 conserved as in *S. cerevisiae*), which was representative of this group. **Figure 8B** shows the structure predicted for CaCis310 (Csp1), a protein with 31 Cys residues (9% of the total) and only three that aligned with the 4 conserved Cys in the model *S. cerevisiae* proteins. The predicted structure showed formation of 5 disulfide bonds. CaCis310 was also notable because of its richness in Asp (20%) and Lys (19%) residues. Lastly, proteins in clade 2, og 4 (**Figure 5**) which included CGOB Groups J, K and L did not have any Cys residues. The predicted structure for CmPir21, which was representative of these groups, is shown in **Figure 8C**. Despite considerably diverse sequences, many of the predicted Pir protein structures shared notable similarities including the core region of antiparallel beta-sheets and extensive loop structures with the potential to form linkages to cell wall components.

### *PIR* Gene Expression

Considerable information about *PIR* gene expression across the various fungal species can be gleaned from analysis of RNA-Seq datasets from the NCBI Sequence Read Archive (https://www.ncbi.nlm.nih.gov/sra). Emphasis was placed on identifying datasets that featured growth in rich medium for species with different complements of *PIR* genes. Because it is often used as a housekeeping control in gene expression analyses (e.g. Niewerth et al., 2003; Nailis et al., 2006), transcriptional activity for the gene encoding actin (*ACT1*) was reported here (**Table 3**). Transcriptional activity of genes encoding glycolytic enzymes was high in the actively growing cells, so enolase (*ENO1*) expression levels were also reported to provide a context for maximal transcriptional activity in each experiment.

**Table 3:** Relative gene expression levels from RNA-Seq datasets available on the NCBI Sequence Read Archive.

C. albicans*	OG	Control	Congo red	*C. auris‡*	OG	WT Growth	*C. glabrata¦*	OG	WT Growth
*CaPIR1*	A	482 ± 85	455 ± 20	*Cau004786*	A	57 ± 13	*GVI51_M08437*	(A)	575 ± 22
*CaPIR32*	B	1 ± 0	1 ± 0	*Cau005431*	(B)	9 ± 3	*GVI51_M08459*	None	234 ± 13
*CaCIS301*	C	0 ± 0	0 ± 0	*Cau003910*	M	1355 ± 315	*GVI51_I05951*	None	534 ± 32
*CaCIS302*	D	15 ± 6	11 ± 2	*CauACT1*	N/A	1341 ± 262	*GVI51_I05973*	None	666 ± 63
*CaCIS303*	E	4 ± 1	3 ± 0	*CauENO1*	N/A	1603 ± 383	*GVI51_I05995*	None	331 ± 21
*CaCIS304*	F	0 ± 0	0 ± 0				*CgACT1*	N/A	1291 ± 64
*CaCIS305*	G	0 ± 0	0 ± 0	***C. lusitaniae* 1**§		**WT Growth**	*CgENO1*	N/A	1233 ± 109
*CaCIS309*	H	18 ± 13	19 ± 3	*Cl05291*	A	1 ± 0			
*CaCIS310*	I	3 ± 1	2 ± 0	*Cl00824*	M	510 ± 62			
*CaCIS312*	J	1 ± 1	1 ± 0	*ClACT1*	N/A	15 ± 1			
*CaCIS308*	None	1 ± 0	1 ± 0	*CaENO1*	N/A	26813 ± 991			
*CaACT1*	N/A	1396 ± 126	1522 ± 1						
*CaENO1*	N/A	7246 ± 1438	6486 ± 284	***C. lusitaniae* 2**§		**WT Growth**			
				*Cl05291*	A	0			
***C. orthopsilosis*** †		**WT Growth**		*Cl00824*	M	1770			
*Co0C02660*	A	415 ± 41		*ClACT1*	N/A	4			
*Co0A08050*	B	3 ± 0		*CaENO1*	N/A	43372			
*Co0D05920*	K	15 ± 0							
*Co0D05640*	L	18 ± 1							
*CoACT1*	N/A	2144 ± 17							
*CoENO1*	N/A	3617 ± 497							

Means and their standard deviations are reported for datasets that had more than one experimental replicate. OG = CGOB ortholog Group (**Supplementary Table S3**).

*C. albicans data were from SRX023480 and SRX023481 (Control), SRX023478 and SRX023479 (Congo red; Bruno et al., 2010). Strain SC5314 from a saturated overnight YPD culture was diluted into fresh medium to OD_600_ = 0.1 then grown at 30°C until OD_600_ = 1.0. The culture was divided into two flasks with 100 μg/ml Congo red added to one and an equal volume of water added to the other. Cultures grew for 2 h at 30°C, cells were harvested by centrifugation then stored at -80°C until RNA was extracted.

^†^C. orthopsilosis data were from SRX1879292 and SRX1879293. Strain Co 90-125 was grown overnight in YPD medium then diluted in fresh medium to OD_600_ = 0.2. The culture was grown at 30°C and 200 rpm shaking for 3.5 h, cells collected by filtration, and RNA extracted.

^‡^C. auris data were from SRX9620787, SRX9620788, and SRX9620789 (Zamith-Miranda et al., 2021). Strain MMC1 was grown in Sabouraud medium.

§C. lusitaniae data were from two different experiments. Experiment 1 was SRR2141706 and SRR2141707 (Froyd et al., 2013; Kapoor et al., 2015). The C. lusitaniae strain was derived from CL143 which is congenic to ATCC 42720. Cells were taken from a culture in the log phase of growth in YPD medium at 30°C. Experiment 2 was SRX10085525, which was aimed at capturing the transcriptome of strain MJ12. Cells were grown in trypticase soy broth at 28°C.

¦C. glabrata data were from SRX707647, SRX707648, and SRX707649 (Linde et al., 2015). Strain ATCC 2001 was grown in YPD medium at 37°C and 180 rpm shaking for 14-16 h, then resuspended in fresh medium and grown for 1 h.

Bruno et al. (2010) conducted RNA-Seq analysis of *C. albicans* under a variety of conditions with the goal of improving annotation of the transcriptome. One experiment involved comparing strain SC5314 growth in YPD medium with and without the addition of Congo red. Analysis of this dataset showed that *CaPIR1* was more active than the other *PIR* genes (**Table 3**). Transcription from 8 of the 11 *PIR* genes including *CaPIR32*, was barely detectable. Compared to *CaPIR1*, *CaCIS302* and *CaCIS309* were next-most-highly transcribed although at a level approximately 25-fold lower. Addition of Congo red made little difference in expression of these genes.

An RNA-Seq dataset for transcriptional profiling of *C. orthopsilosis* was also examined (**Table 3**). *C. orthopsilosis* has an ortholog of *CaPIR1* (*Co0C02660*; CGOB Group A), an ortholog of *CaPIR32* (*Co0A08050*; Group B) and one gene each from Group K (*Co0D05920*) and Group L (*Co0D05640*). Group K was unique to the *C. parapsilosis* species complex (that includes *C. parapsilosis*, *C. orthopsilosis*, and *C. metapsilosis*; Tavanti et al., 2005) while Group L also had an ortholog in *C. tropicalis* (**Supplementary Table S3**). Similar to *C. albicans*, the Group A *C. orthopsilosis* ortholog was the most highly expressed during growth in rich medium and the Group B ortholog was transcriptionally quiet. The Group K and L orthologs showed similar expression levels that were higher than Group B, but still relatively low compared to the Group A ortholog.

Datasets were specifically sought from fungi that had a CGOB Group M ortholog since these were only found in a subset of the species and, like Group A genes, tended to have many copies of the *PIR* repeat unit (**Supplementary Table S3**). Growth of *C. auris* cells in YPD medium showed higher expression for the Group A ortholog compared to Group B, but considerably higher expression levels for the Group M ortholog (**Table 3**). This same trend was noted for *C. lusitaniae*, which had only two *PIR* genes (**Supplementary Table S3**). Transcription from the Group M ortholog was far higher than from the Group A ortholog. (**Table 3**).

A *C. glabrata* dataset was also sought since this species, like *S. cerevisiae*, had 5 *PIR* genes and a similar arrangement of loci in the genome (**Supplementary Figure S10**). Analysis of an RNA-Seq dataset derived from cells grown in YPD medium showed a relatively equal and strong contribution of expression from each *PIR* locus (**Table 3**). Relative gene expression data collected from these public datasets established different categories of expression patterns for the *PIR* genes in the various species.

## Discussion

The initial goal for this work was construction of a null mutant strain with all *PIR* loci deleted. Since *C. albicans* was expected to only have two *PIR* genes, it was selected as the target species despite literature warnings that the effort may not be successful. For example, Martínez et al. (2004) could not isolate a *Δpir1/Δpir1* null strain and suggested that *PIR1* may be essential in *C. albicans*. However, a *Δpir1/Δpir1* strain was included in a collection resulting from a large-scale *C. albicans* gene deletion effort that used *HIS1* and *LEU2* as selectable markers (Noble et al., 2010). The *Δpir1/Δpir1* mutant in that collection did not show differences in growth rate or cellular morphology compared to a parental control. Deletion of *C. albicans PIR32* was reported to affect cellular morphology (Bahnan et al., 2012). In contrast, the current study showed that deletion of *PIR1* and/or *PIR32* resulted in strains with indistinguishable growth rate and morphology compared to the parent. Notable differences in growth rate and cellular morphology between mutant and parent strains likely account for the phenotypic effects observed for previous *PIR* disruption efforts (Martínez et al., 2004; Bahnan et al., 2012; **Table 1**).

The unexpected lack of phenotypic difference between the *Δpir/Δpir* null strain and its parental control led to a larger investigation of *PIR* genes in *C. albicans* and beyond. Because of their shared features and sequence motifs, the 75 proteins across the 16 fungal species studied are proposed to belong to the Pir family (**Supplementary Table S3**). This family is far larger than previously described, particularly in *C. albicans* and *C. dubliniensis* which have 11 and 10 Pir proteins, respectively. Overall, Pir proteins are small (approximately 200 to 400 amino acids). Pir proteins have obvious repeated units (such as in CGOB Groups A and M) or a remnant of the repeated motif, minimally DGQ, that likely functions in linkage to cell wall β-1,3-glucan as demonstrated for *S. cerevisiae* Pir4 (Ecker et al., 2006). Ecker et al. (2006) hypothesized that carboxyl ester bond formation by the Q in multiple repeat copies would stabilize and strengthen a mesh network in the cell wall, creating a structure that is similar to bacterial peptidoglycan. The position of the repeat unit Q residues in the AlphaFold structures presented here supports this idea (**Figure 7B**).

Alignment of the Pir sequences revealed another consensus sequence motif, QFQFD, located toward the C-terminal end of each protein for which function is still uncharacterized. Presence of Q residues in the QFQFD motif prompt speculation that they may also be involved in linkage of the protein to β-1,3-glucan. For loci that do not have extensive copy numbers of the repeated motif DGQ, the presence of the C-terminal motif QFQFD may provide additional opportunity for constructing important linkages. Or, perhaps, the function of QFQFD is entirely different since studies in *S. cerevisiae* Pir4 that led to identification of the Q74 contribution were done with a protein that presumably had an intact QFQFD sequence (Ecker et al., 2006). It is also formally possible that the motifs have somewhat different roles in various species, another hypothesis that requires investigation.

Unlike some *Candida* gene families in which repeated sequence variation leads to a tremendous number of alleles (Zhang et al., 2003; Hoyer et al., 2008; Oh et al., 2021), *PIR* genes are more highly constrained in repeat size and copy number. Predicted protein structures also unify the family showing a core structure of antiparallel beta-sheets surrounded by loops of varying size and complexity depending on sequence composition. The role of the core structure is unknown, while the loops are proposed to serve as the basis for broad contact with other fungal cell wall components that promotes wall strength (Ecker et al., 2006). *S. cerevisiae* Pir proteins are heavily O-glycosylated and that feature is predicted for many of the other Pir proteins described here, particularly the orthologs in CGOB Group A (**Supplementary Table S3**). One notable exception is the predicted near-complete lack of O-glycosylation for *C. dubliniensis* Pir proteins, which also remains to be understood.

Public gene expression datasets were analyzed to form additional hypotheses about the larger *PIR* family. Although *CaPIR1* expression was considerably higher than *CaPIR32*, *CaPIR1* expression was modest during growth in rich medium (**Table 3**). *CaPIR1* was singled out in a microarray-based experiment as one of the most highly expressed genes during regeneration of protoplasts (Castillo et al., 2006). *CaPIR1* showed a 2.7-fold increase in expression at 30 min and a 6-fold increase at 6 h during the regeneration process. A similar experiment with *S. cerevisiae* showed up-regulation of *ScPIR1*, *ScPIR2*, and *ScPIR3* during protoplast regeneration (Castillo et al., 2008). Perhaps the *Δpir1-1/Δpir1-2 Δpir32-1/Δpir32-2* strain described here would display defects during protoplast regeneration. Testing this idea, as well as assessing mutant vs. parent gene expression differences during growth in rich medium, could shed additional light on the role of Pir proteins in *C. albicans* and possibly reveal compensatory gene expression mechanisms within *C. albicans*.

Although most of the *C. albicans PIR* genes are not expressed during yeast cell growth in rich medium, several are highly expressed during chlamydospore formation. Chlamydospore formation has been used traditionally as a diagnostic identification aid (Walsh et al., 2018). Staib and Morschhäuser (2005) noted that *C. albicans* chlamydospore formation is enhanced by deletion of *NRG1*. In contrast, wild-type *C. dubliniensis* forms an abundance of chlamydospores under conditions where wild-type *C. albicans* grows as a budding yeast (Staib and Morschhäuser, 1999). RNA-Seq analysis of wild-type *C. albicans*, *Δnrg1 C. albicans*, and wild-type *C. dubliniensis* cells grown under chlamydospore-forming conditions yielded gene expression data that were compared to identify highly up-regulated genes (Palige et al., 2013; **Supplementary Table S2**). *C. albicans* orf19.3512 (*CIS310*) and its ortholog *C. dubliniensis Cd30750* were named *CSP1* while *C. albicans* orf19.4170 (*CIS309*) and its *C. dubliniensis* ortholog *Cd40770* were named *CSP2*. Green fluorescent protein (GFP) fusions for each *C. dubliniensis* protein were localized to the chlamydospore cell wall (Palige et al., 2013). Jansons and Nickerson (1970) described the chemical composition of *C. albicans* chlamydospores noting an outer layer comprised of glucan (presumably β-1,3-linked) and a small amount of chitin. It is possible that the Pir proteins interact with chlamydospore β-1,3-glucan similarly to what has been demonstrated for *S. cerevisiae* yeast cells (Ecker et al., 2006). Single deletions of *CSP1* or *CSP2* in *C. dubliniensis* did not reveal differences in chlamydospore production, germ tube formation, or sensitivity to calcofluor white, Congo red, or oxidative stress (Palige et al., 2013). Expression of other *PIR* loci likely masked the effect of mutation (**Supplementary Table S2**).

Moving away from *C. albicans* on the phylogenetic tree (Li et al., 2021) reveals species with unique complements of *PIR* genes. For example, CGOB Groups K and L emerge in the closely related species *C. parapsilosis*, *C. orthopsilosis*, and *C. metapsilosis* (**Supplementary Table S3** and **Figure 5**). Analysis of *C. orthopsilosis* RNA-Seq data from growth in rich medium showed that the Group A ortholog is most highly expressed and just like *C. albicans*, the other *C. orthopsilosis PIR* genes are barely transcribed (**Table 3**). Perhaps expression of the Group K and L genes has a specialized purpose that has yet to be identified. It is worth noting that *C. tropicalis*, located between *C. albicans* and *C. parapsilosis* on the phylogenetic tree, has a CGOB Group L ortholog. *C. tropicalis* also has Ctr01767, the ancestral gene that gave rise to the *C. albicans* and *C. dubliniensis* chlamydospore-associated genes (**Figure 5**).

Data presented here suggest additional investigation into the chlamydospore-forming ability of *C. tropicalis*. *Larone’s Medically Important Fungi* (Walsh et al., 2018) recognizes infrequent production of teardrop-shaped chlamydospores by *C. tropicalis* even though other sources (e.g. Palige et al., 2013; Hernández-Cervantes et al., 2020) consider *C. tropicalis* as chlamydospore-negative. The work by Hernández-Cervantes et al. (2020) elegantly demonstrated that the transcription factor Rme1 activates expression of genes that are up-regulated during chlamydospore formation in *C. albicans* and *C. dubliniensis*. The authors showed that *RME1* expression levels in *C. albicans* correlate positively with chlamydospore-formation phenotype. The authors also showed that overexpression of *C. tropicalis RME1* in a *C. albicans Δrme1/Δrme1* strain cannot restore chlamydospore formation. However, they did not test whether overexpression of *C. albicans RME1* in *C. tropicalis* could activate latent chlamydospore-formation capabilities, elements of which are visible in the data presented here.

*C. auris* and *C. lusitaniae* (Family *Metschnikowiaceae*) are even-farther removed from *C. albicans* (Family *Debaryomycetaceae*) on the phylogenetic tree of species (Li et al., 2021). These species are of interest because they have a CGOB Group M *PIR* ortholog (**Supplementary Table S3**). In *C. auris* growing in rich medium, the Group M ortholog was expressed over 20-fold more highly than the Group A ortholog (**Table 3**). The same pattern was observed in two independent *C. lusitaniae* RNA-Seq datasets. These results suggest *C. lusitaniae* as the species in which a complete *Δpir* null mutant could be pursued since only two *PIR* loci are present. A more-marked phenotype would be expected compared to the *C. albicans* work detailed here. Group M alleles are also present in the *Debaryomycetaceae* species *Y. tenuis*, *S. passalidarum*, *S. stipitis*, *M. guilliermondii*, and *D. hansenii* (**Supplementary Table S3**), often as one gene in a total of 2 or 3 *PIR* loci in each species. RNA-Seq analysis would reveal whether the Group M allele plays the central role during rich-medium growth for these species, as observed for *C. auris* and *C. lusitaniae*.

Characterization of Pir function was conducted primarily in *S. cerevisiae* as detailed above. As each of four *PIR* genes (*ScPIR1*, *ScPIR2*, *ScPIR3*, *ScPIR4*) were deleted sequentially, the resulting mutant strains revealed increasingly notable cell-wall-defect phenotypes (Mrša and Tanner, 1999; Mazáň et al., 2008). These observations suggest a meaningful contribution from each gene to cell wall structure. Of the other species studied here, *C. glabrata* is most closely related to *S. cerevisiae*; both are in the Family *Saccharomycetaceae*. RNA-Seq analysis showed that each of the five *C. glabrata PIR* genes is transcribed at similar levels during growth in rich medium. Expression levels were high, approximately 20% to 50% of levels observed for *ACT1* and *ENO1* (**Table 3**). Sequential *PIR* gene deletion in *C. glabrata* would likely reveal a similar phenotype to that observed in *S. cerevisiae*.

Knowledge about the fungal cell wall is important because the cell wall is essential for cell integrity and central to considerations regarding antifungal development. There is no doubt that Pir proteins are part of the cell wall in the species examined to date (Mrša and Tanner 1999; Martínez et al., 2004; Sumita et al., 2005; Ecker et al., 2006). Four of the *S. cerevisiae* Pir proteins have been used as fusion partners to direct other proteins to cell-surface localization (Shimma et al., 2006) and GFP fusions to *C. albicans/C. dubliniensis* Csp proteins are localized to the cell wall (Palige et al., 2013; Hernández-Cervantes et al., 2020). Perhaps Pir proteins can serve as a marker for studying cell wall structural variation among the pathogenic species. Many unknowns remain to be addressed. For example, while there is agreement that Pir proteins are linked to β-1,3-glucan with an alkali-labile bond, the nature of proposed disulfide linkages (Moukadiri and Zueco, 2001) is not yet understood. Castillo et al. (2003) used site-directed mutagenesis and constructed deletion mutants to identify the Cys residue(s) involved in the linkage. Their work did not identify the Cys residue, but did not test C120, the residue implicated by AlphaFold to be available for disulfide linkage to the cell wall (**Figure 7A**). The nature of Pir protein release to the extracellular medium also requires further study (Russo et al., 1992). Moreover, it is still unclear how much information can be generalized across species and even among proteins within the same species. The ideas presented here provide an initial roadmap to pursue additional knowledge about the Pir proteins, their comparative structure and function, and their contributions to cell integrity.

## Materials and Methods

### Microbial Strains and Culture Conditions

All microbial strains were stored at -80°C in 38% glycerol. Microbial strains were streaked to agar plates and incubated for 16 h at 37°C. Plates were stored at 4°C for no more than one week.

*C. albicans* SC5314 was used as a reference and as the background for strain constructions. *C. albicans* strains are listed in **Supplementary Table S5**. *C. albicans* strains isolated from healthy humans and from wildlife species were described previously (Wrobel et al., 2008). *E. coli* TOP10 and TOP10 F’ (Thermo Fisher Scientific) were used for cloning and plasmid propagation.

*C. albicans* growth media included YPD (per liter: 10 g yeast extract, 20 g peptone, 20 g dextrose), YPM (per liter: 10 g yeast extract, 20 g peptone, 20 g maltose), potato dextrose broth (PDB; per liter: infusion from boiling 200 g of potatoes for 30 min, filtered through cheesecloth, 20 g dextrose), and yeast nitrogen base (YNB; per liter: 6.7 g yeast nitrogen base, 5 g glucose). *E. coli* was grown in LB medium (per liter: 10 g tryptone, 5 g sodium chloride, 5 g yeast extract). Liquid growth media were solidified by addition of 20 g of Bacto agar per liter for fungal growth and 15 g Bacto agar per liter for bacterial growth. All media, except YNB, was sterilized by autoclaving. YNB was sterilized by filtration across a 0.4 µm pore-size filter (Thermo Fisher Scientific). Growth media were supplemented as needed. Details are presented in the method for each experiment.

### DNA Extraction, Amplification, and Analysis

Plasmids were purified from *E. coli* transformants using a Speed Prep method (Goode and Feinstein, 1992). Larger-scale plasmid preparations used an alkaline lysis protocol (Birnboim and Doly, 1979). *C. albicans* genomic DNA was extracted using the MasterPure Yeast DNA Purification Kit (Epicentre). Modifications to the manufacturer’s instructions included omitting the ethanol wash step, dissolving the precipitated nucleic acid in Tris EDTA buffer (10 mm Tris-HCl, pH 8.0, 1 mM EDTA) and incubating with RNase at 37°C for 2 h. DNA concentration was measured spectrophotometrically.

Oligonucleotide primers were designed using the PrimerQuest Tool (www.idtdna.com) and synthesized by Integrated DNA Technologies (Coralville, IA). For PCR, a typical 50-µl reaction included 10 µl of 5× buffer containing 15 mM MgCl_2_, 5 µl of each PCR primer (10 µM final concentration), 4 µl of 2.5 mM dNTPs, 200 ng of DNA template, 0.125 units of DNA polymerase (Taq polymerase from Invitrogen or Q5 polymerase from New England Biolabs), and water to 50 µl. Standard PCR conditions were 95°C for 5 min followed by 25 cycles of 95°C (30 sec), 55°C (30 sec), 72°C (1 min) then 7 min at 72°C. PCR products were analyzed on 1% agarose gels in Tris-Acetate EDTA buffer (TAE; 40 mM Tris pH 7.6, 20 mM acetic acid, 1 mM EDTA). Gels were stained with ethidium bromide and visualized using ultraviolet light.

For cloning, PCR fragments were excised with a scalpel. DNA was purified from the agarose slice using a GeneClean III kit (QBiogene). Purified DNA was ligated into vector pJET-TA (Fermentas) and transformed into *E. coli* TOP10. The transformants were screened by PCR using a portion of each colony as the template. Primers flanked the insert fragment in the vector. Products were analyzed by agarose gel electrophoresis and those of the expected size selected for DNA sequence analysis. Cloning of DNA fragments from restriction enzyme digestion followed a similar method; enzymes were used according to manufacturer’s instructions.

Sanger DNA sequencing reactions were completed at the Roy J. Carver Biotechnology Center DNA Services Lab (University of Illinois at Urbana-Champaign, Urbana, IL). Plasmids from selected clones were purified using the Wizard DNA Clean-Up System (Promega). DNA sequences were analyzed using Chromas Lite software (Technelysium Pty Ltd, South Brisbane, Australia).

### Deletion of *PIR1* in *C. albicans*

The *SAT1* flipper method of gene disruption was used to delete both alleles of *PIR1* (Reuß et al., 2004). **Supplementary Figure S15** shows a summary of the process for deleting both *PIR1* alleles in strain SC5314. Plasmids used and created during the study are listed in **Supplementary Table S6**. Primers are detailed in **Supplementary Table S7**.

Integration of the deletion cassette into the *PIR1* locus was directed by cloning DNA from upstream and downstream of *PIR1* into the 5’ and 3’ polylinker regions in plasmid 3027. Specifically, the downstream flanking region of *PIR1* was amplified with the primer pair PIR1- dnF/PIR1-dnR to yield a 859-bp fragment that was cloned into *Sac*II-*Sac*I-digested plasmid 3027 to yield plasmid 3054 (**Supplementary Table S6**). Plasmid 3054 was digested with *Kpn*I-*Xho*I and a 948-bp PCR product (amplified with primers PIR1-upF/PIR1-upR) ligated into it to yield plasmid 3059. Approximately 30 µg of plasmid 3059 was digested with *Kpn*I-*Sac*I to release the deletion cassette. The plasmid preparation was visualized on an agarose gel to ensure that digestion was complete. The digestion was extracted with phenol-choloroform-isoamyl alcohol and DNA precipitated with ethanol. DNA was resuspended in nuclease-free water at a concentration of approximately 2 µg/ml.

The deletion cassette was transformed into *C. albicans* strain SC5314 using a lithium acetate method (Ramon and Fonzi, 2009). SC5314 cells from a 16-h YPD culture were counted using a hemacytometer and resuspended at a density of 2 × 10^6^ cells/ml in 50 ml YPD in a 250-ml flask. The culture was incubated at 30°C and 200 rpm shaking. When the cell density reached 1-2 × 10^7^ cells/ml (approximately 4 h of incubation), the cells were collected by centrifugation in a 50-ml conical tube. Cells were washed twice with sterile deionized water, resuspended in a 1-ml volume and transferred to a microfuge tube. The cell pellet was washed with 1 ml of sterile 1× TE-LiAc (Sasse and Morschhäuser, 2012) and cells collected by centrifugation. The cells were resuspended to a density of 2 × 10^9^ cells/ml in 1× TE-LiAc and the tube placed on ice.

Each transformation reaction included at least 10 µg of the DNA cassette and 5 µg of denatured salmon sperm (carrier) DNA. A viability control and a negative control were used, each with deionized water instead of DNA. Fifty µl of LiAc-treated SC5314 cells were added to each transformation reaction, followed by 300 µl of 40% PEG 3350 solution and mixing by pipetting. Tubes were incubated for 16 h at 30°C with gentle rotation. Tubes were heat shocked at 44°C for 15 min, and cells collected by centrifugation. The cell pellet was resuspended in sterile deionized water and the volume was divided equally onto two YPD plates containing 100 µg/ml nourseothricin (Gold Biotechnology). The negative control reaction was also plated on YPD with nourseothricin, while the viability control was plated on YPD. Plates were incubated at 30°C for 2-4 days to allow transformants to grow.

After transformation, the nourseothricin resistance marker was removed by inducing the expression of ca*FLP* (encoding the *C. albicans* Flp recombinase), which is regulated by a *MAL2* promoter. Once expressed, the caFlp recombinase excises the nourseothricin resistance gene between two *FRT* target sequences. ca*FLP* expression was induced by growing the *C. albicans* culture in a maltose-containing mediumum (10 ml of YPM at 30°C for 16 h). The culture was diluted, plated on YPD with 25 µg/ml nourseothricin, and incubated at 30°C for 24 h. Colonies from the YPM-grown cells were picked and streaked on YPD or YPD with 200 µg/ml nourseothricin. The plates were incubated at 30°C for 24 h. Colonies that only grew on the YPD plate were selected. Southern blotting was used to demonstrate the deletion of the *PIR1* coding region.

Southern blotting used the Genius System (Boehringer Mannheim Biochemicals) according to the manufacturer’s instructions. Two probes were prepared. The first was a *PIR1* downstream fragment that was amplified with primer pair PIR1-dnF/PIR1-dnR and the second was the *PIR1* coding region that was amplified with primer pair PIR1-CDF/PIR1-CDR. The amplified fragments were purified with GeneClean and labelled by incorporation of digoxigenin-modified nucleotides.

Transformation of *C. albicans* parent strain SC5314 with *PIR1*-deletion plasmid 3059 yielded strains 3077 and 3080 (**Supplementary Table S5**). Strain 3077 had *PIR1-1* removed while strain 3080 had the *PIR1-2* removed. Excision of the nourseothricin resistance marker from strains 3077 and 3080 produced strains 3082 and 3083, respectively. Strain 3082 was transformed again with plasmid 3059 to result in strain 3087. Removal of the nourseothricin resistance gene from strain 3087 produced strain 3097, a *Δpir1/Δpir1* null mutant. Primer pair PIR1 DnOrfCheckF/PIR1 DnOrfCheckR was used to amplify and sequence the starting portion of orf19.223 to ensure that its sequence was not altered by the insertion of the *PIR1* deletion cassette in strain 3097 (**Supplementary Figure S15**).

### Deletion of *PIR32* in Wild-Type and Δ*pir1*/Δ*pir1 C. albicans* Strains

The *SAT1* cassette was used to delete *PIR32* alleles in *C. albicans*, as described above for *PIR1*. **Supplementary Figures S16**, **S17** summarize this process. The upstream flanking region of *PIR32* was amplified with primer pair PIR32 Kpn upF/PIR32 Xho upR to yield a 597-bp fragment that was ligated into *Kpn*I-*Xho*I-digested plasmid 3027. The resulting plasmid was called 3502 and was digested with *Sac*II-*Sac*I to incorporate a 134-bp PCR fragment of the downsteam flanking region that was amplified using primers PIR32 SacII dnF/PIR32 SacI dnR. The resulting plasmid, 3505, was transformed into *C. albicans* strain SC5314 with the intention of creating a *Δpir32/Δpir32* strain. The cassette integrated into the *PIR32-1* allele, creating strain 3521 (**Supplementary Table S4**). Excision of the deletion cassette resulted in strain 3524.

Because plasmid 3505 would not integrate into the second *PIR32* allele despite multiple attempts, another deletion cassette, plasmid 3529, was constructed. The *PIR32* flanking regions in plasmid 3529 were altered to include only sequences that remain in the *PIR32-2* allele in strain 3524. This fragment was amplified using primers Pir32 SacII FII/Pir32 SacI RII, producing a 326-bp fragment that was cloned into the *Sac*II-*Sac*I-digested plasmid. The resulting plasmid was called 3529. Transformation of strain 3524 with plasmid 3529 yielded strain 3537 in which both *PIR32* alleles were deleted. Removal of the deletion cassette resulted in strain 3543, a *Δpir32/Δpir32* null mutant.

The *PIR32* deletion cassettes were also used to transform strain 3097 (*Δpir1/Δpir1*) with the intention of constructing a double-null strain (*Δpir1/Δpir1 Δpir32/Δpir32*). **Supplementary Figure S18** summarizes this process. Plasmid 3505 was used to transform 3097, yielding strain 3511, from which the *PIR32-1* allele was deleted. Excision of the cassette resulted in strain 3516. Plasmid 3529 was used to transform strain 3516 to yield strain 3540, from which both *PIR32* alleles were deleted in the *Δpir1/Δpir1* background. The deletion cassette was removed to produce strain 3543, the double-null mutant.

All *PIR32* strain constructions were validated using PCR. The primer pairs NatCheck F/Pir32 InsrtCheckR, Pir32 allA F/Pir32 allA R and Pir32 allB F/Pir32 allB R were used to verify the strains 3521, 3537, 3511 and 3540. The primer pairs Pir32 allA F/Pir32 allA R and Pir32 allB F/Pir32 allB R were designed so the 5’ end of the primers were specific for one of the two alleles of *PIR32*. Thus, amplification with each primer set was specific to one of the two *PIR32* alleles. This primer set was used to keep track of which *PIR32* was deleted. The primer pair NatCheck F/Pir32 InsrtCheckR had the forward primer within the deletion cassette and the reverse primer in the region outside of the deletion cassette, downstream of where the cassette was inserted. Genomic DNA from the strains, extracted as described above, was used for PCR. The genomic DNA from SC5314 was used as a positive control for all PCR.

### Growth Rate Measurement

A single *C. albicans* colony from a YPD agar plate was inoculated into 20 ml YPD in a 50-ml flask. This starter culture was incubated for 16 h at 30°C and 200 rpm shaking. Cells were counted using a hemacytometer and inoculated into 20 ml fresh YPD at a density of 1 × 10^6^ cells/ml. Cultures were incubated at 30°C and 200 rpm shaking. OD_620_ readings were taken in triplicate at the 0 h time point and each hour afterward. Growth rates were measured on three different days from separate starter cultures. Statistical analysis involved calculating rate of growth and doubling time from the linear portion of the growth curve using the exponential growth equation in nonlinear regression in GraphPad Prism (GraphPad Software). The same software was used to assess the statistical differences between the growth rates using a one-way ANOVA.

### Assessment of *C. albicans* Colony and Cellular Morphology

*C. albicans* isolates were streaked from -80°C glycerol vials to YPD plates and grown for 16 h at 37°C. This stock plate was stored at 4°C for no more than one week. *C. albicans* morphology was assessed in various ways, as described for *Δpir* strains (Bahnan et al., 2012). A single colony from the YPD plate was streaked onto a Potato Dextrose Agar (PDA) plate. One plate was incubated for 24 h at 37°C and another for 14 d at 28°C. Plates were photographed and compared for differences in colony morphology. The experiment was repeated to ensure reproducibility.

Morphology of yeast cells was assessed by inoculating a single *C. albicans* colony from the stock plate into 20 ml Potato Dextrose Broth (PDB) in a 50-ml flask. The flask was incubated at 37°C with 200 rpm shaking for 16 h. An aliquot of the culture was placed onto a microscope slide and a representative field of view was photographed. Observations were repeated on at least one additional, independent occasion.

The ability of *C. albicans* strains to form a germ tube and the relative rate of germ tube formation were also assessed. A 16-h PDB starter culture was grown as described above. Cells were washed in Dulbecco’s Phosphate-Buffered Saline without Ca^2+^ or Mg^2+^ (DPBS) and counted using a hemacytometer. Cells were inoculated into four different growth conditions that promote germ tube formation: prewarmed RPMI 1640 without L-glutamine (RPMI; Invitrogen catalog no. 11875-085), Spider medium (Liu et al., 1994), YPD with 10% fetal bovine serum (FBS), or PDB with 20% FBS. Cells were inoculated at a density 5 × 10^6^ cells/ml in 10 ml of medium in a 50-ml flask. The flasks were incubated at 37°C shaking at 200 rpm and 100 µl samples collected at 20 min, 40 min and 60 min, then fixed with 4% (v/v) paraformaldehyde. Three different time points were used because rates of germ tube formation were growth-medium-dependent. The goal was to identify at least one time point where differences in germ tube formation could be evaluated. Samples were viewed microscopically with 100 cells evaluated for each growth condition. Cells with a germ tube longer than one diameter of the mother yeast cell were considered germ-tube-positive while cells with shorter or no germ tube were called germ-tube-negative. Replicate observations were collected for independent cultures on at least three different days. The mean values were calculated and a mixed model analysis of variance (PROC MIXED in SAS) was used to assess differences in germ tube formation.

Morphology of hyphae was studied for the control and *Δpir* strains. A single colony from a YPD stock culture plate was inoculated into 10 ml of FBS in a 50-ml conical tube. Cultures were incubated at 28°C or 37°C. An aliquot was removed from each culture at 2 h, 4 h, 6 h, 8 h, and 10 h and cells were fixed in 4% (v/v) paraformaldehyde. Cells were observed under a light microscope and photographed to document results.

### Evaluation of Phenotype in Response to Stress Conditions

Assays that involved spotting dilutions of *C. albicans* parent and mutant isolates onto agar plates were used to evaluate phenotype of the mutant strains in response to various stress conditions. Specialized agar plates were prepared as described below and many different assays were conducted on the same day. Experimental plates were matched with the control plate from the same assay day and presented in figures throughout the paper. Results are presented in separate figures, rather than combining all results into one image, to preserve detail in the figure legend. As such, the same control plate appears in multiple figures. Details about assay replication are presented below.

To test growth in the presence of osmotic stress, a single *C. albicans* colony from a YPD agar stock plate was inoculated into 20 ml PDB in a 50-ml flask. This starter culture was incubated for 16 h at 37°C and 200 rpm shaking. Cells were collected by centrifugation and washed twice in DPBS. Cells were diluted, counted using a hemacytometer, and resuspended in DPBS at a density of 1 × 10^8^ cells/ml. Serial 10-fold dilutions were prepared in DPBS and 5 µl of each (using dilutions ranging from 10^6^ to 10^3^ cells/ml) spotted onto the surface of PDA plates that incorporated either 1.0 M or 1.5 M sodium chloride. Control plates that did not contain sodium chloride were also prepared to monitor cell growth in the absence of osmotic stress. One set of plates was incubated at 28°C and another at 37°C for 48 h. Plates were photographed to document results. The experiment was repeated at least twice in its entirety.

To test growth following oxidative stress, *C. albicans* PDB or YPD starter cultures and washed dilutions of cells were prepared as described above. Dilutions of cells (ranging from 2 × 10^6^ to 2 × 10^3^ cells/ml) in DPBS were added 1:1 to 100 mM hydrogen peroxide (diluted in DPBS) for a final concentration of 50 mM. Cultures were incubated 1 h at room temperature, then spotted onto PDA or YPD plates. One set of plates was incubated at 28°C and another at 37°C for 48 h. Control cells that were not exposed to hydrogen peroxide were also plated and incubated. Plates were photographed to document results. The experiment was repeated at least twice in its entirety.

To test growth following heat shock, *C. albicans* PDB starter cultures were prepared as described above. Dilutions of washed cells (using dilutions ranging from 10^6^ to 10^3^ cells/ml) in DPBS were incubated for 3 h in a 42°C water bath, then for 20 min at 28°C. Cells were spotted onto PDA plates. One set of plates was incubated at 28°C and another at 37°C for 48 h. Control cells that were not exposed to heat shock were also plated and incubated. Plates were photographed to document results. The experiment was repeated at least twice in its entirety.

To test sensitivity to cell-wall-disrupting agents, *C. albicans* PDB starter cultures were prepared as described above. Serial 10-fold dilutions of cells were prepared in DPBS and 5 µl of each (using dilutions ranging from 10^6^ to 10^3^ cells/ml) were spotted onto the surface of PDA plates that incorporated various cell-wall-disrupting agents. Supplements included calcofluor white (150 µg/ml), Congo red (30 and 50 µg/ml), cystamine (50 mM), Hygromycin B (100 µg/ml), and SDS (0.03%). Concentrations for various agents were determined from the literature (Martínez et al., 2004; Bahnan et al., 2012) and empirically. One set of plates was incubated at 28°C and another at 37°C for 48 h. Plates were photographed to document results. To provide complete comparisons of newly constructed *C. albicans* strains to published reports of *Δpir* phenotypes, the same assay was conducted for calcofluor white and Congo red using *C. albicans* cells that were pre-grown in YPD or YNB, then spotted onto YPD and/or YNB agar plates. Experiments were repeated in their entirety on at least two independent occasions.

### Sensitivity to Antifungal Drugs

Antifungal drug sensitivity was measured using the Sensititre YeastOne broth microdilution plate (catalog number YO-9; TREK Diagnostic Systems, Thermo Scientific). *C. albicans* colonies for the assay were taken from YPD agar stock plates. Colony material was added to 5 ml of Sensititre demineralized water (catalog number T2339) to achieve a 0.5 McFarland turbidity. Twenty ml of this suspension was added to Sensititre YeastOne inoculum broth (Catalog number Y3462) and a Sensititre dosehead attached to the inoculated broth. After mixing, 100 μl of the broth was dispensed into each well of the microdilution plate. The plate was sealed with adhesive and incubated at 33°C for 24 h. The positive control well was checked for a red color and the plate evaluated.

### Biofilm Formation on Polystyrene

The method was adapted from Bahnan et al. (2012). A 24-well, flat-bottomed polystyrene plate was coated with 5% FBS at 4°C overnight. A single *C. albicans* colony from a YPD stock plate was used to grow a PDB starter culture as described above. Cells were washed in DPBS, counted using a hemacytometer and resuspended at a concentration of 1 × 10^7^ cells/ml. FBS was removed from each well and 500 µl of the *C. albicans* cell suspension added. The plate was incubated at 37°C for 3 h and 75 rpm on a rotary shaker. Wells were washed twice with DPBS, then 1 ml YNB added. The plates were returned to incubation at 37°C for 48 h and 75 rpm shaking. Wells were washed with DPBS to remove nonadherent *C. albicans* cells and the growth fixed with 99% methanol for 15 min. Methanol was removed and the plate air-dried for 20 min, then 500 µl of 0.2% crystal violet added for 20 min. Wells were washed 5× with distilled water to remove excess crystal violet. Crystal violet was released from the plate with 750 µl of 33% acetic acid. The released crystal violet was diluted 1:10 and 1:20 with 33% acetic acid and the absorbance read at 590 nm. The experiment was conducted on three separate occasions. Each experimental replicate included six individual observations (wells) for each *C. albicans* strain. *C. albicans* strains were randomly assigned to a well in the 24-well plate to avoid positional effects. A mixed model analysis of variance was used to study the difference in adherence to BEC. Data were analyzed using PROC MIXED in SAS. Separation of means was performed with the LSMEANS option.

### Adhesion to Buccal Epithelial Cells (BECs)

The assay was conducted as described previously (Zhao et al., 2004). BECs were collected from five human volunteer donors and pooled. Each donor provided written consent for participation in the study and collection procedures followed the guidelines of the University of Illinois Institutional Review Board. Cells were washed twice in DPBS and counted using a hemacytometer. Cells were resuspended at a concentration of 8 × 10^4^ cells/ml and kept on ice. *C. albicans* strains were inoculated from a stock YPD plate into 10 ml liquid PDB and grown for 16 h at 37°C with 200 rpm shaking. *C. albicans* cells were washed in DPBS and counted. For the adhesion assay with germ tubes, 2 × 10^6^ C*. albicans* cells were inoculated into 4 ml RPMI in a 25-ml flask that was incubated at 37°C for 1 h with 200 rpm shaking. Then, 2 × 10^4^ BEC were added to each flask that now contained germ tubes. For adhesion of the yeast form of *C. albicans*, 2 × 10^4^ BEC and 2 × 10^7^ C*. albicans* cells were combined in 4 ml of DPBS in a 25-ml Erlenmeyer flask. The flask was incubated at 37°C and 200 rpm shaking for 30 min. Cells were vacuum filtered across 12 µm pore size Nucleopore polycarbonate filters (Corning catalog number 111116). Filters were washed dropwise with 25 ml DPBS to remove nonadherent *C. albicans* cells. Filters were removed from the vacuum filtration device, inverted onto glass microscope slides and dried. Following removal of the filter from the slide, slides were heat fixed, stained with crystal violet, washed with tap water, dried and examined microscopically. The number of *C. albicans* cells adhering to the first 100 BEC observed on the center of each slide was recorded. Replicates for each strain were conducted on three separate days using a different pool of BEC on each day. Results for the germ tube adhesion assay were expressed as the mean number of *C. albicans* germ tubes that adhered to each BEC. Results for the yeast cell adhesion assay were reported as the total number of yeast cells adhering to 100 BECs. A mixed model analysis of variance was used to study the difference in adherence to BEC. The mean number of adherent germ tubes for each replicate within a strain per day was analyzed using PROC MIXED in SAS. Separation of means was performed with the LSMEANS option.

### *C. metapsilosis PIR* Sequences

Candidate *PIR* genes from *Candida metapsilosis* ATCC 96143 were amplified by PCR using methods described above, then verified by Sanger sequencing. Primer sequences were included in **Supplementary Table S7**. Amplification and cloning of *CmPIR11* yielded two clear alleles, both of which were deposited into GenBank. GenBank accession numbers for the *CmPIR* genes were MT017922 (*CmPIR01*), OL539426 (*CmPIR11-1*), OL539427 (*CmPIR11-2*), MT017923 (*CmPIR21*), MT017924 (*CmPIR22*), MT017925 (*CmPIR23*), MT017926 (*CmPIR24*), and MT017927 (*CmPIR25*).

### Bioinformatic Analyses

*C. albicans* sequences were downloaded from the *Candida* Genome Database (CGD; http://www.candidagenome.org; Skrzypek et al., 2017). “A” alleles from the diploid genome assembly were used preferentially. *S. cerevisiae* sequences were downloaded from the *Saccharomyces* Genome Database (SGD; http://www.yeastgenome.org; Engel et al., 2014). Database searches used BLAST *via* the utility on either the CGD, SGD, or NCBI website (https://blast.ncbi.nlm.nih.gov/Blast.cgi).

Sequence alignments were generated using Clustal Omega (http://www.ebi.ac.uk/Tools/msa/clustalo; Madeira et al., 2019). EMBOSS Transeq (http://www.ebi.ac.uk/Tools/st/emboss_transeq) or ExPASy (http://web.expasy.org/translate) were used to translate DNA sequences.

The *Candida* Gene Order Browser (CGOB; Maguire et al., 2013) was used to assign the *PIR* ortholog groups (**Supplementary Table S3**). SignalP-5.0 Server (http://www.cbs.dtu.dk/services/SignalP; Nielsen, 2017) was used to locate putative secretory signal peptides. European Bioinformatics Institute (EMBL-EBI) tools were used for translating nucleotide sequences, sequence alignment, and other general processes (https://www.ebi.ac.uk/services; Cook et al., 2017). ProP 1.0 was used to predict arginine and lysine propeptide cleavage sites (https://services.healthtech.dtu.dk/service.php?ProP-1.0; Duckert et al., 2004). N-linked glycosylation sites were predicted using NetNGly – 1.0 (https://services.healthtech.dtu.dk/service.php?NetNGlyc-1.0; Gupta and Brunak, 2002). O-linked glycosylation sites were predicted using NetOGlyc – 4.0 (https://services.healthtech.dtu.dk/service.php?NetOGly-4.0; Steentoft et al., 2013).

Fastq datasets were downloaded from NCBI Sequence Read Archive (https://www.ncbi.nlm.nih.gov/sra). STAR was used to map sequence reads to the reference genome (Dobin and Gingeras, 2016). featureCounts was used for read summarization (Liao et al., 2014). Relative gene expression levels were compared following normalization of read counts to gene length and total reads in the experiment.

### Phylogenetic Analysis

Phylogeny of the *PIR* family was estimated based on the amino acid sequence alignment created with PROMALS3D (Pei et al., 2008). Poorly aligned regions were eliminated using Gblocks v 0.91b with options allowing the least stringent selection (Castresana, 2000). There were only 96 positions retained in the final alignment from a total of 928 in the original alignment. Model selection was performed using ModelFinder (Kalyaanamoorthy et al., 2017) implemented in IQ-TREE (Nguyen et al., 2015); WAG+I+G4 was chosen as a best-fit model according to the Bayesian information criterion. The maximum likelihood tree was constructed with IQ-TREE v. 1.6.12 with nodal support determined by nonparametric bootstrapping with 1000 replicates. Subsequent phylogenetic analyses were conducted separately for clades 2 and 3 resulting from the first analysis to retain a higher number of positions in the alignments and consequently increase the chance to identify tentative orthologous groups in the dataset. There were 145 and 158 positions retained in the alignments of clade 2 and clade 3, respectively, and WAG+I+G4 model was chosen in both cases. The trees were constructed as described above.

### Protein Structural Predictions

Protein structural predictions were derived using AlphaFold (Jumper et al., 2021), accessed using AlphaFold Colab (https://colab.research.google.com/github/deepmind/alphafold/blob/main/notebooks/AlphaFold.ipynb). ColabFold (Mirdita et al., 2021; https://colab.research.google.com/github/sokrypton/ColabFold/blob/main/AlphaFold2.ipynb) was used for more-rapid generation of structural prototypes. ColabFold utilized MMSeqs2 (UniRef+Environmental). Structural visualization and alignments used the PyMOL Molecular Graphics System, Version 2.5.2 (Schrödinger, LLC).

## Data Availability Statement

The datasets presented in this study can be found in online repositories. The names of the repository/repositories and accession number(s) can be found in the article/**Supplementary Material**.

## Ethics Statement

The studies involving human participants were reviewed and approved by University of Illinois at Urbana-Champaign Office for the Protection of Research Subjects. The patients/participants provided their written informed consent to participate in this study.

## Author Contributions

LH conceptualized the study, acquired funding, and was responsible for project administration. VH conducted formal analysis. JK, S-HO, RR-B, XZ, and LH performed the investigation. JK, S-HO, RR-B, VH, XZ, and LH developed the study methodology. LH wrote the original draft. All authors contributed to the article and approved the submitted version.

## Funding

This work was funded by R01 DE14158 and R15 DE026401 from the National Institute of Dental and Craniofacial Research, National Institutes of Health.

## Conflict of Interest

The authors declare that the research was conducted in the absence of any commercial or financial relationships that could be construed as a potential conflict of interest.

## Publisher’s Note

All claims expressed in this article are solely those of the authors and do not necessarily represent those of their affiliated organizations, or those of the publisher, the editors and the reviewers. Any product that may be evaluated in this article, or claim that may be made by its manufacturer, is not guaranteed or endorsed by the publisher.

## References

[B1] Bahnan W. Koussa J. Younes S. Abi Rizk M. Khalil B. El Sitt S.. (2012). Deletion of the *Candida albicans PIR32* Results in Increased Virulence, Stress Response, and Upregulation of Cell Wall Chitin Deposition. Mycopathologia 174, 107–119. doi: 10.1007/s11046-012-9533-z 22391823

[B2] Birnboim H. C. Doly J. (1979). A Rapid Alkaline Extraction Procedure for Screening Recombinant Plasmid DNA. Nucleic Acids Res. 7, 1513–1523. doi: 10.1093/nar/7.6.1513 388356 PMC342324

[B3] Bruno V. M. Wang Z. Marjani S. L. Euskirchen G. M. Martin J. Sherlock G.. (2010). Comprehensive Annotation of the Transcriptome of the Human Fungal Pathogen *Candida albicans* Using RNA-Seq. Genome Res. 20, 1451–1458. doi: 10.1101/gr.109553.110 20810668 PMC2945194

[B4] Castillo L. Martínez A. I. Garcerá A. Elorza M. V. Valentín E. Sentandreu R. (2003). Functional Analysis of the Cysteine Residues and the Repetitive Sequence of *Saccharomyces cerevisiae* Pir4/Cis3: The Repetitive Sequence is Needed for Binding to the Cell Wall β-1,3-Glucan. Yeast 20, 973–983. doi: 10.1002/yea.1016 12898712

[B5] Castillo L. Martínez A. I. Garcerá A. García-Martínez J. Ruiz-Herrera J. Valentín E.. (2006). Genomic Response Programs of *Candida albicans* Following Protoplasting and Regeneration. Fungal Genet. Biol. 43, 124–134. doi: 10.1016/j.fgb.2005.12.002 16455273

[B6] Castillo L. Martinez A. I. Gelis S. Ruiz-Herrera J. Valentin E. Sentandreu R. (2008). Genomic Response Programs of *Saccharomyces cerevisiae* Following Protoplasting and Regeneration. Fungal Genet. Biol. 45, 253–265. doi: 10.1016/j.fgb.2007.10.002 18032075

[B7] Castresana J. (2000). Selection of Conserved Blocks From Multiple Alignments for Their Use in Phylogenetic Analysis. Mol. Biol. Evol. 17, 540–542. doi: 10.1093/oxfordjournals.molbev.a026334 10742046

[B8] Chaffin W. L. (2008). *Candida albicans* Cell Wall Proteins. Microbiol. Mol. Biol. Rev. 72, 495–544. doi: 10.1128/MMBR.00032-07 18772287 PMC2546859

[B9] Chaffin W. L. López-Ribot J. L. Casanova M. Gozalbo D. Martínez J. P. (1998). Cell Wall and Secreted Proteins of *Candida albicans*: Identification, Function, and Expression. Microbiol. Mol. Biol. Rev. 62, 130–180. doi: 10.1128/MMBR.62.1.130-180.1998 9529890 PMC98909

[B10] Cook C. E. Bergman M. T. Cochrane G. Apweiler R. Birney E. (2017). The European Bioinformatics Institute in 2017: Data Coordination and Integration. Nucleic Acids Res. 46, D21–D29. doi: 10.1093/nar/gkx1154 PMC5753251 29186510

[B11] Dobin A. Gingeras T. R. (2016). Optimizing RNA-Seq Mapping With STAR. Methods Mol. Biol. 1415, 245–262. doi: 10.1007/978-1-4939-3572-7_13 27115637

[B12] Duckert P. Brunak S. Blom N. (2004). Prediction of Proprotein Convertase Cleavage Sites. Prot. Eng. Des. Sel. 17, 107–112. doi: 10.1093/protein/gzh013 14985543

[B13] Ecker M. Deutzmann R. Lehle L. Mrša V. Tanner W. (2006). Pir Proteins of *Saccharomyces cerevisiae* are Attached to β-1,3-Glucan by a New Protein-Carbohydrate Linkage. J. Biol. Chem. 281, 11523–11529. doi: 10.1074/jbc.M600314200 16495216

[B14] Engel S. R. Dietrich F. S. Fisk D. G. Binkley G. Balakrishnan R. Costanzo M. C.. (2014). The Reference Genome Sequence of *Saccharomyces cerevisiae*: Then and Now. G3 4, 389–398. doi: 10.1534/g3.113.008995 24374639 PMC3962479

[B15] Froyd C. A. Kapoor S. Dietrich F. Rusche L. N. (2013). The Deacetylase Sir2 From the Yeast *Clavispora lusitaniae* Lacks the Evolutionarily Conserved Capacity to Generate Subtelomeric Heterochromatin. PLoS Genet. 9, e1003935. doi: 10.1371/journal.pgen.1003935 24204326 PMC3814328

[B16] Gillum A. M. Tsay E. Y. Kirsch D. R. (1984). Isolation of the *Candida albicans* Genes for Orotidine-5’-Phosphate Decaryboxylase by Complementation of *S. cerevisiae ura3* and *E. voli pyrF* Mutations. Mol. Gen. Genet. 198, 179–182. doi: 10.1007/BF00328721 6394964

[B17] Goffeau A. Barrell B. G. Bussey H. Davis R. W. Dujon B. Feldmann H.. (1996). Life With 6000 Genes. Science 274(546), 563–567. doi: 10.1126/science.274.5287.546 8849441

[B18] Goode B. L. Feinstein S. C. (1992). “Speedprep” Purification of Template for Double-Stranded DNA Sequencing. BioTechniques 12, 374–375. 1571144

[B19] Gow N. A. Hube B. (2012). Importance of the *Candida albicans* Cell Wall During Commensalism and Infection. Curr. Opin. Microbiol. 15, 406–412. doi: 10.1016/j.mib.2012.04.005 22609181

[B20] Gupta R. Brunak S. (2002). Prediction of Glycosylation Across the Human Proteome and the Correlation to Protein Function. Pac. Symp. Biocomput. 2002, 310–322. 11928486

[B21] Hernández-Cervantes A. Znaidi S. van Wijlick L. Denega I. Basso V. Ropars J.. (2020). A Conserved Regulator Controls Asexual Sporulation in the Fungal Pathogen *Candida albicans* . Nat. Commun. 11, 6224. doi: 10.1038/s41467-020-20010-9 33277479 PMC7718266

[B22] Hoyer L. L. Green C. B. Oh S.-H. Zhao X. (2008). Discovering the Secrets of the *Candida albicans* Agglutinin-Like Sequence (*ALS*) Gene Family – a Sticky Pursuit. Med. Mycol. 46, 1–15. doi: 10.1080/13693780701435317 17852717 PMC2742883

[B23] Jansons V. K. Nickerson W. J. (1970). Chemical Composition of Chlamydospores of *Candida albicans* . J. Bacteriol. 104, 922–932. doi: 10.1128/jb.104.2.922-932.1970 4099099 PMC285076

[B24] Jumper J. Evans R. Pritzel A. Green T. Figurnov M. Ronneberger O.. (2021). Highly Accurate Protein Structure Prediction With AlphaFold. Nature 596, 583–589. doi: 10.1038/s41586-021-03819-2 34265844 PMC8371605

[B25] Kalyaanamoorthy S. Minh B. Q. Wong T. K. F. von Haeseler A. Jermiin L. S. (2017). ModelFinder: Fast Model Selection for Accurate Phylogenetic Estimates. Nat. Methods 14, 587–589. doi: 10.1038/nmeth.4285 28481363 PMC5453245

[B26] Kandasamy R. Vediyappan G. Chaffin W. L. (2000). Evidence for the Presence of Pir-Like Proteins in *Candida albicans* . FEMS Microbiol. Lett. 186, 239–243. doi: 10.1111/j.1574-6968.2000.tb09111.x 10802178 PMC4833757

[B27] Kapoor S. Zhu L. Froyd C. Liu T. Rusche L. N. (2015). Regional Centromeres in the Yeast *Candida lusitaniae* Lack Pericentromeric Heterochromatin. Proc. Natl. Acad. Sci. U. S. A. 112, 12139–12144. doi: 10.1073/pnas.1508749112 26371315 PMC4593121

[B28] Kapteyn J. C. Hoyer L. L. Hecht J. E. Müller W. H. Andel A. Verkleij A. J.. (2000). The Cell Wall Architecture of *Candida albicans* Wild-Type Cells and Cell Wall-Defective Mutants. Mol. Microbiol. 35, 601–611. doi: 10.1046/j.1365-2958.2000.01729.x 10672182

[B29] Kapteyn J. C. Van Egmond P. Sievi E. Van Den Ende H. Makarow M. Klis F. M. (1999). The Contribution of the O-Glycosylated Protein Pir2p/Hsp150 to the Construction of the Yeast Cell Wall in Wild-Type Cells and β1,6-Glucan-Deficient Mutants. Mol. Microbiol. 31, 1835–1844. doi: 10.1046/j.1365-2958.1999.01320.x 10209754

[B30] Liao Y. Smyth G. K. Shi W. (2014). featureCounts: An Efficient General Purpose Program for Assigning Sequence Reads to Genomic Features. Bioinformatics 30, 923–930. doi: 10.1093/bioinformatics/btt656 24227677

[B31] Linde J. Duggan S. Weber M. Horn F. Sieber P. Hellwig D.. (2015). Defining the Transcriptomic Landscape of *Candida glabrata* by RNA-Seq. Nucleic Acids Res. 43, 1392–1406. doi: 10.1093/nar/gku1357 25586221 PMC4330350

[B32] Li Y. Steenwyk J. L. Chang Y. Wang Y. James T. Y. Stajich J. E.. (2021). A Genome-Scale Phylogeny of the Kingdom Fungi. Curr. Biol. 26, 1653–1665. doi: 10.1016/j.cub.2021.01.074 PMC8347878 33607033

[B33] Liu H. Kohler J. Fink G. R. (1994). Suppression of Hyphal Formation in *Candida albicans* by Mutation of a *STE12* Homolog. Science 266, 1723–1726. doi: 10.1126/science.799205 7992058

[B34] Lu C. F. Kurjan J. Lipke P. N. (1994). A Pathway for Cell Wall Anchorage of *Saccharomyces cerevisiae* alpha-Agglutinin. Mol. Cell. Biol. 14, 4825–4833. doi: 10.1128/mcb.14.7.4825-4833.1994 8007981 PMC358855

[B35] Madeira M. Park Y. M. Lee J. Buso N. Gur T. Madhusoodanan N.. (2019). The EMBL-EBI Search and Sequence Analysis Tools APIs in 2019. Nucleic Acids Res. 47, W636–W641. doi: 10.1093/nar/gkz268 30976793 PMC6602479

[B36] Maguire S. L. ÓhÉigeartaigh S. S. Byrne K. P. Schröder M. S. O’Gaora P. Wolfe K. H.. (2013). Comparative Genome Analysis, and Gene Finding in *Candida* Species Using CGOB. Mol. Biol. Evol. 30, 1281–1291. doi: 10.1093/molbev/mst042 23486613 PMC3649674

[B37] Manning B. D. Padmanabha R. Snyder M. (1997). The Rho-GEF Rom2p Localizes to Sites of Polarized Cell Growth and Participates in Cytoskeletal Functions in *Saccharomyces cerevisiae* . Mol. Biol. Cell 8, 1829–1844. doi: 10.1091/mbc.8.10.1829 9348527 PMC25625

[B38] Martínez A. I. Castillo L. Garcerá A. Elorza M. V. Valentín E. Sentandreu R. (2004). Role of Pir1 in the Construction of the *Candida albicans* Cell Wall. Microbiology 150, 3151–3161. doi: 10.1099/mic.0.27220-0 15470096

[B39] Mazáň M. Mazáňová K. Farkaš V. (2008). Phenotype Analysis of *Saccharomyces cerevisiae* Mutants With Deletions in Pir Cell Wall Glycoproteins. Antonie Van Leeuwenhoek 94, 335–342. doi: 10.1007/s10482-008-9228-0 18278564

[B40] Mirdita M. Schutze K. Moriwaki Y. Heo L. Ovchinnikov S. Steinegger M. (2021). ColabFold – Making Protein Folding Accessible to All. bioRxiv Preprint. doi: 10.1101/2021.08.15.456425 PMC9184281 35637307

[B41] Moukadiri I. Jaafar L. Zueco J. (1999). Identification of Two Mannoproteins Released From Cell Walls of a *Saccharomyces cerevisiae mnn1 mnn9* Double Mutant by Reducing Agents. J. Bacteriol. 181, 4741–4745. doi: 10.1128/JB.181.16.4741-4745.1999 10438739 PMC93956

[B42] Moukadiri I. Zueco J. (2001). Evidence for the Attachment of Hsp150/Pir2 to the Cell Wall of *Saccharomyces cerevisiae* Through Disulfide Bridges. FEMS Yeast Res. 1, 241–245. doi: 10.1111/j.1567-1364.2001.tb00040.x 12702350

[B43] Mrša V. Seidl T. Gentzsch M. Tanner W. (1997). Specific Labelling of Cell Wall Proteins by Biotinylation. Identification of Four Covalently Linked O-Mannosylated Proteins of *Saccharomyces cerevisiae* . Yeast 13, 1145–1154. doi: 10.1002/(SICI)1097-0061(19970930)13:12<1145::AID-YEA163>3.0.CO;2-Y 9301021

[B44] Mrša V. Tanner W. (1999). Role of NaOH-Extractable Cell Wall Proteins Ccw5p, Ccw6p, Ccw7p and Ccw8p (Members of the Pir Protein Family) in Stability of the *Saccharomyces cerevisiae* Cell Wall. Yeast 15, 813–820. doi: 10.1002/(SICI)1097-0061(199907)15:10A<813::AID-YEA421>3.0.CO;2-Y 10407261

[B45] Nailis H. Coenye T. Van Nieuwerburgh F. Deforce D. Nelis H. J. (2006). Development and Evaluation of Different Normalization Strategies for Gene Expression Studies in *Candida albicans* Biofilms by Real-Time PCR. BMC Mol. Biol. 7, 25. doi: 10.1186/1471-2199-7-25 16889665 PMC1557526

[B46] Nguyen L.-T. Schmidt H. A. von Haeseler A. Minh B. Q. (2015). IQ-TREE: A Fast and Effective Stochastic Algorithm for Estimating Maximum-Likelihood Phylogenies. Mol. Biol. Evol. 32, 268–274. doi: 10.1093/molbev/msu300 25371430 PMC4271533

[B47] Nielsen H. (2017). “Predicting Secretory Proteins With SignalP,” in Protein Function Prediction, Methods and Protocols, vol. 1611. Ed. Kihara D. (New York, NY: Humana Press), 59–73. doi: 10.1007/978-1-4939-7015-5_6 28451972

[B48] Niewerth M. Kunze D. Seibold M. Schaller M. Korting H. C. Hube B. (2003). Ciclopirox Olamine Treatment Affects the Expression Pattern of *Candida albicans* Genes Encoding Virulence Factors, Iron Metabolism Proteins, and Drug Resistance Factors. Antimicrob. Agents Chemother. 47, 1805–1817. doi: 10.1128/AAC.47.6.1805-1817.2003 12760852 PMC155814

[B49] Noble S. M. French S. Kohn L. A. Chen V. Johnson A. D. (2010). Systematic Screens of a *Candida albicans* Homozygous Deletion Library Decouple Morphogenetic Switching and Pathogenicity. Nat. Genet. 42, 590–598. doi: 10.1038/ng.605 20543849 PMC2893244

[B50] Northcote D. H. Horne R. W. (1952). The Chemical Composition and Structure of the Yeast Cell Wall. Biochem. J. 51, 232–236. doi: 10.1042/bj0510232 14944578 PMC1197826

[B51] Odds F. C. Bougnoux M.-E. Shaw D. J. Bain J. M. Davidson A. D. Diogo D.. (2007). Molecular Phylogenetics of *Candida albicans* . Eukaryot. Cell 6, 1041–1052. doi: 10.1128/EC.00041-07 17416899 PMC1951527

[B52] Oh S.-H. Cheng G. Nuessen J. A. Jajko R. Yeater K. M. Zhao X.. (2005). Functional Specificity of *Candida albicans* Als3p Proteins and Clade Specificity of *ALS3* Alleles Discriminated by the Number of Copies of the Tandem Repeat Sequence in the Central Domain. Microbiology 151, 673–681. doi: 10.1099/mic.0.27680-0 15758214

[B53] Oh S.-H. Schliep K. Isenhower A. Rodriguez-Bobadilla R. Vuong V. M. Fields C. J.. (2021). Using Genomics to Shape the Definition of the Agglutinin-Like Sequence (*ALS*) Family in the Saccharomycetales. Front. Cell. Infect. Microbiol. 11, 794529. doi: 10.3389/fcimb.2021.794529 34970511 PMC8712946

[B54] Palige K. Linde J. Martin R. Böttcher B. Citiulo F. Sullivan D. J.. (2013). Global Transcriptome Sequencing Identifies Chlamydospore Specific Markers in *Candida albicans* and *Candida dubliniensis* . PLoS One 8, e61940. doi: 10.1371/journal.pone.0061940 23613980 PMC3626690

[B55] Pei J. Kim B.-H. Grishin N. V. (2008). PROMALS3D: A Tool for Multiple Protein Sequence and Structure Alignments. Nucleic Acids Res. 36, 2295–2300. doi: 10.1093/nar/gkn072 18287115 PMC2367709

[B56] Ramon A. Fonzi W. (2009). “Genetic Transformation of *Candida albicans* ,” in Candida albicans, Methods and Protocols. Eds. Cihlar R. Calderone R. (New York, NY: Humana Press), 169–174.

[B57] Reuß O. Vik Å. Kolter R. Morschhäuser J. (2004). The *SAT1* Flipper, an Optimized Tool for Gene Disruption in *Candida albicans* . Gene 341, 119–127. doi: 10.1016/j.gene.2004.06.021 15474295

[B58] Ruiz-Herrera J. Elorza M. V. Valentín E. Sentandreu R. (2006). Molecular Organization of the Cell Wall of *Candida albicans* and its Relation to Pathogenicity. FEMS Yeast Res. 6, 14–29. doi: 10.1111/j.1567-1364.2005.00017.x 16423067

[B59] Russo P. Kalkkinen N. Sareneva H. Paakkola J. Makarow M. (1992). A Heat Shock Gene from *Saccharomyces cerevisiae* Encoding a Secretory Glycoprotein. Proc. Natl. Acad. Sci. U. S. A. 89, 3671–3675. doi: 10.1073/pnas.89.9.3671 1570286 PMC525552

[B60] Sasse C. Morschhäuser J. (2012). “Gene Deletion in *Candida albicans* Wild-Type Strains Using the *SAT1*-Flipping Strategy,” in Host-Fungus Interactions, Methods and Protocols. Eds. Brand A. C. MacCallum D. M. (New York, NY: Humana Press), 3–17. 10.1007/978-1-61779-539-8_1 22328364

[B61] Shimma Y-I Saito F. Oosawa F. Jigami Y. (2006). Construction of a Library of Human Glycosylatransferases Immobilized in the Cell Wall of *Saccharomyces cerevisiae* . Appl. Environ. Microbiol. 72, 7003–7012. doi: 10.1128/AEM.01378-06 16936046 PMC1636194

[B62] Skrzypek M. S. Binkley J. Binkley G. Miyasato S. R. Simison M. Sherlock G. (2017). The *Candida* Genome Database (CGD): Incorporation of Assembly 22, Systematic Identifiers and Visualization of High Throughput Sequencing Data. Nucleic Acids Res. 45, D592–D596. doi: 10.1093/nar/gkw924 27738138 PMC5210628

[B63] Staib P. Morschhäuser J. (1999). Chlamydospore Formation on Staib Agar as a Species-Specific Characteristic of *Candida dubliniensis* . Mycoses 42, 521–524. doi: 10.1046/j.1439-0507.1999.00516.x 10592694

[B64] Staib P. Morschhäuser J. (2005). Differential Expression of the *NRG1* Repressor Controls Species-Specific Regulation of Chlamydospore Development in *Candida albicans* and *Candida dubliniensis* . Mol. Microbiol. 55, 637–652. doi: 10.1111/j.1365-2958.2004.04414.x 15659176

[B65] Steentoft C. Vakhrushev S. Y. Joshi H. J. Kong Y. Vester-Christensen M. B. Schjoldager K. T.. (2013). Precision Mapping of the Human O-GalNAc Glycoproteome Through SimpleCell Technology. EMBO J. 32, 1478–1488. doi: 10.1038/emboj.2013.79 23584533 PMC3655468

[B66] Sumita T. Yoko-o T. Shimma Y. Jigami Y. (2005). Comparison of Cell Wall Localization Among Pir Family Proteins and Functional Dissection of the Region Required for Cell Wall Binding and Bud Scar Recruitment of Pir1p. Eukaryot. Cell 4, 1872–1881. doi: 10.1128/EC.4.11.1872-1881.2005 16278454 PMC1287866

[B67] Tavanti A. Davidson A. D. Gow N. A. Maiden M. C. Odds F. C. (2005). *Candida orthopsilosis* and *Candida metapsilosis* spp. nov. Replace *Candida parapsilosis* Groups II and III. J. Clin. Microbiol. 43, 284–292. doi: 10.1128/JCM.43.1.284-292.2005 15634984 PMC540126

[B68] Toh-E A. Yasunaga S. Nisogi H. Tanaka K. Oguchi T. Matsui Y. (1993). Three Yeast Genes, *PIR1*, *PIR2* and *PIR3*, Containing Internal Tandem Repeats, are Related to Each Other, and *PIR1* and *PIR2* are Required for Tolerance to Heat Shock. Yeast 9, 481–494. doi: 10.1002/yea.320090504 8322511

[B69] Walsh T. J. Hayden R. T. Larone D. H. (2018). Larone’s Medically Important Fungi: A Guide to Identification. 6th edition (Washington, DC: ASM Press). doi: 10.1128/9781555819880

[B70] Wrobel L. Whittington J. K. Pujol C. Oh S.-H. Ruiz M. O. Pfaller M. A.. (2008). Molecular Phylogenetic Analysis of a Geographically and Temporally Matched Set of *Candida albicans* Isolates From Humans and Nonmigratory Wildlife in Central Illinois. Eukaryot. Cell 7, 1475–1486. doi: 10.1128/EC.00162-08 18621922 PMC2547061

[B71] Xu Z. Green B. Benoit N. Schatz M. Wheelan S. Cormack B. (2020). *De novo* Genome Assembly of *Candida glabrata* Reveals Cell Wall Protein Complement and Structure of Dispersed Tandem Repeat Arrays. Mol. Microbiol. 113, 1209–1224. doi: 10.1111/mmi.14488 32068314

[B72] Zamith-Miranda D. Amatuzzi R. F. Munhoz da Rocha I. F. Martins S. T. Lucena A. C. R. Vieira A. Z.. (2021). Transcriptional and Translational Landscape of *Candida auris* in Response to Caspofungin. Comput. Struct. Biotech. J. 19, 5264–5277. doi: 10.1016/j.csbj.2021.09.007 PMC8481930 34630944

[B73] Zhang N. Harrex A. L. Holland B. R. Fenton L. E. Cannon R. D. Schmid J. (2003). Sixty Alleles of the *ALS7* Open Reading Frame in *Candida albicans: ALS7* Is a Hypermutable Contingency Locus. Genome Res. 13, 2005–2017. doi: 10.1101/gr.1024903 12952872 PMC403672

[B74] Zhao X. Oh S.-H. Cheng G. Green C. B. Nuessen J. A. Yeater K.. (2004). *ALS3* and *ALS8* Represent a Single Locus That Encodes a *Candida albicans* Adhesin; Functional Comparisons Between Als3p and Als1p. Microbiology 150, 2415–2428. doi: 10.1099/mic.0.26943-0 15256583

