# Supplementary material for: Peering Into Candida albicans Pir Protein Function and Comparative Genomics of the Pir Family
Source: Front Cell Infect Microbiol. 2022 Mar 18;12:836632. doi: 10.3389/fcimb.2022.836632 (PMC8975586; doi:10.3389/fcimb.2022.836632)
Supplement: Supplementary file 1 [file Presentation_1.pptx]

## Slide 1
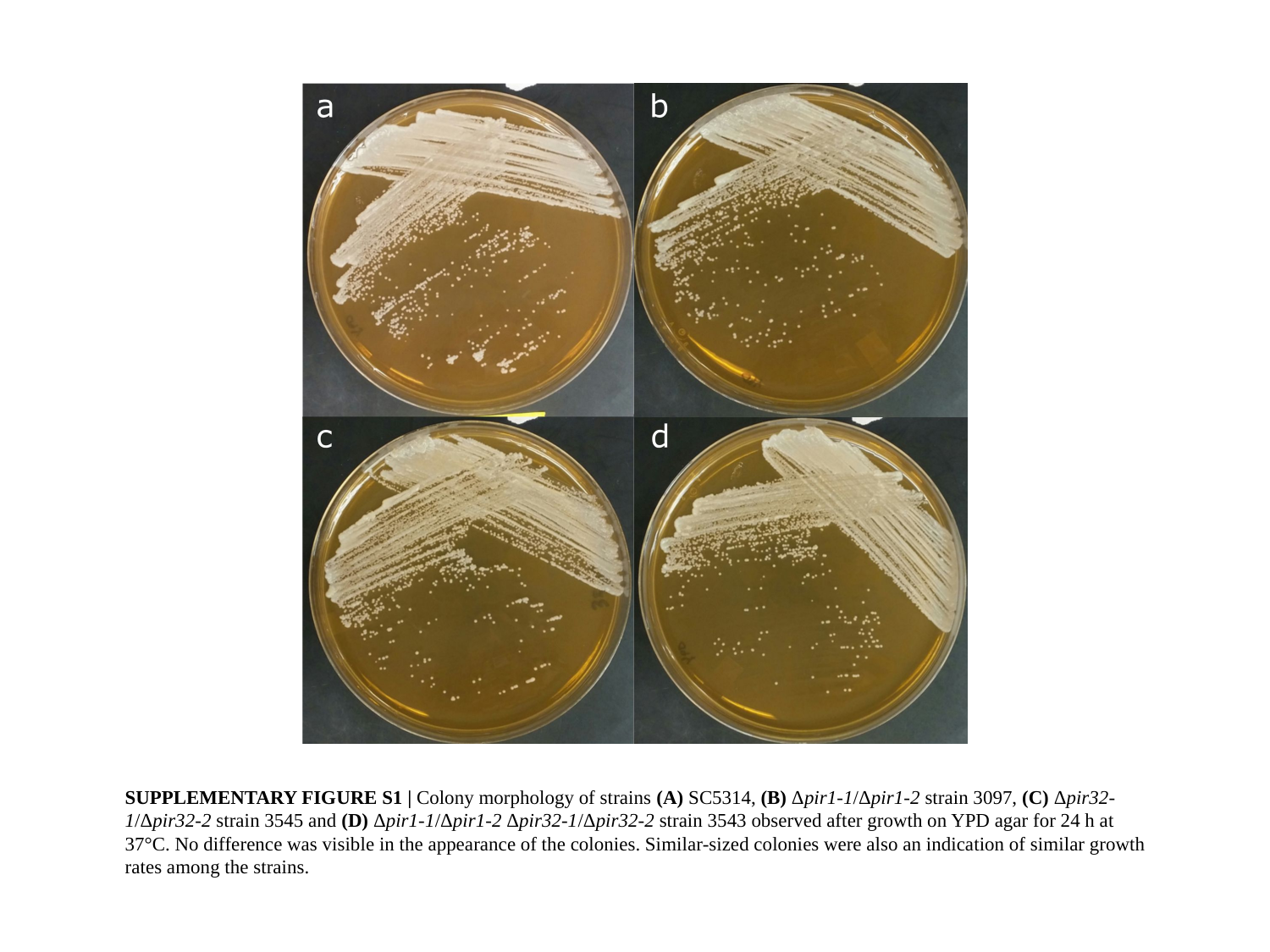

SUPPLEMENTARY FIGURE S1 | Colony morphology of strains (A) SC5314, (B) Δpir1-1/Δpir1-2 strain 3097, (C) Δpir32- 1/Δpir32-2 strain 3545 and (D) Δpir1-1/Δpir1-2 Δpir32-1/Δpir32-2 strain 3543 observed after growth on YPD agar for 24 h at 37°C. No difference was visible in the appearance of the colonies. Similar-sized colonies were also an indication of similar growth rates among the strains.

## Slide 2
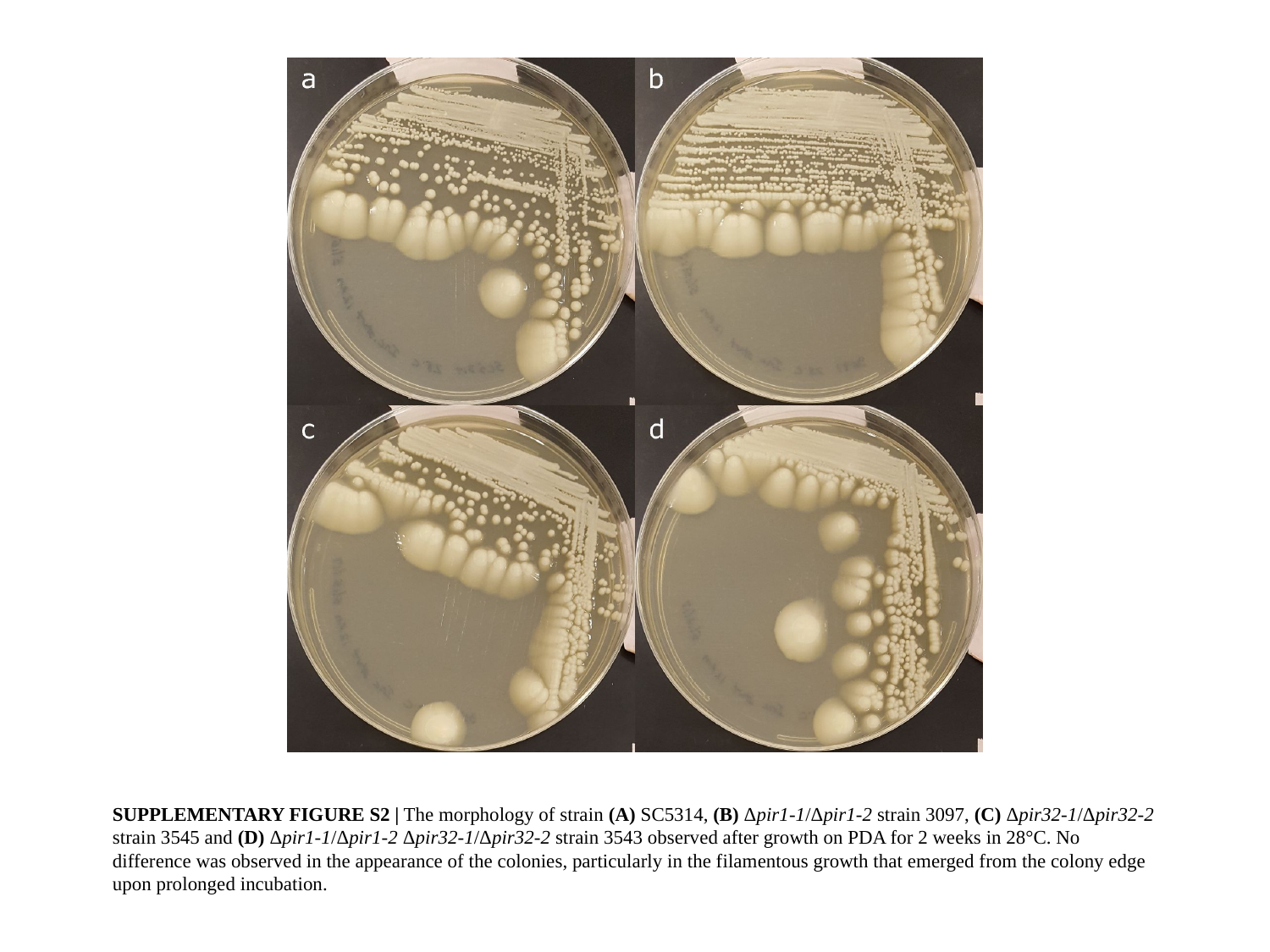

SUPPLEMENTARY FIGURE S2 | The morphology of strain (A) SC5314, (B) Δpir1-1/Δpir1-2 strain 3097, (C) Δpir32-1/Δpir32-2 strain 3545 and (D) Δpir1-1/Δpir1-2 Δpir32-1/Δpir32-2 strain 3543 observed after growth on PDA for 2 weeks in 28°C. No difference was observed in the appearance of the colonies, particularly in the filamentous growth that emerged from the colony edge upon prolonged incubation.

## Slide 3
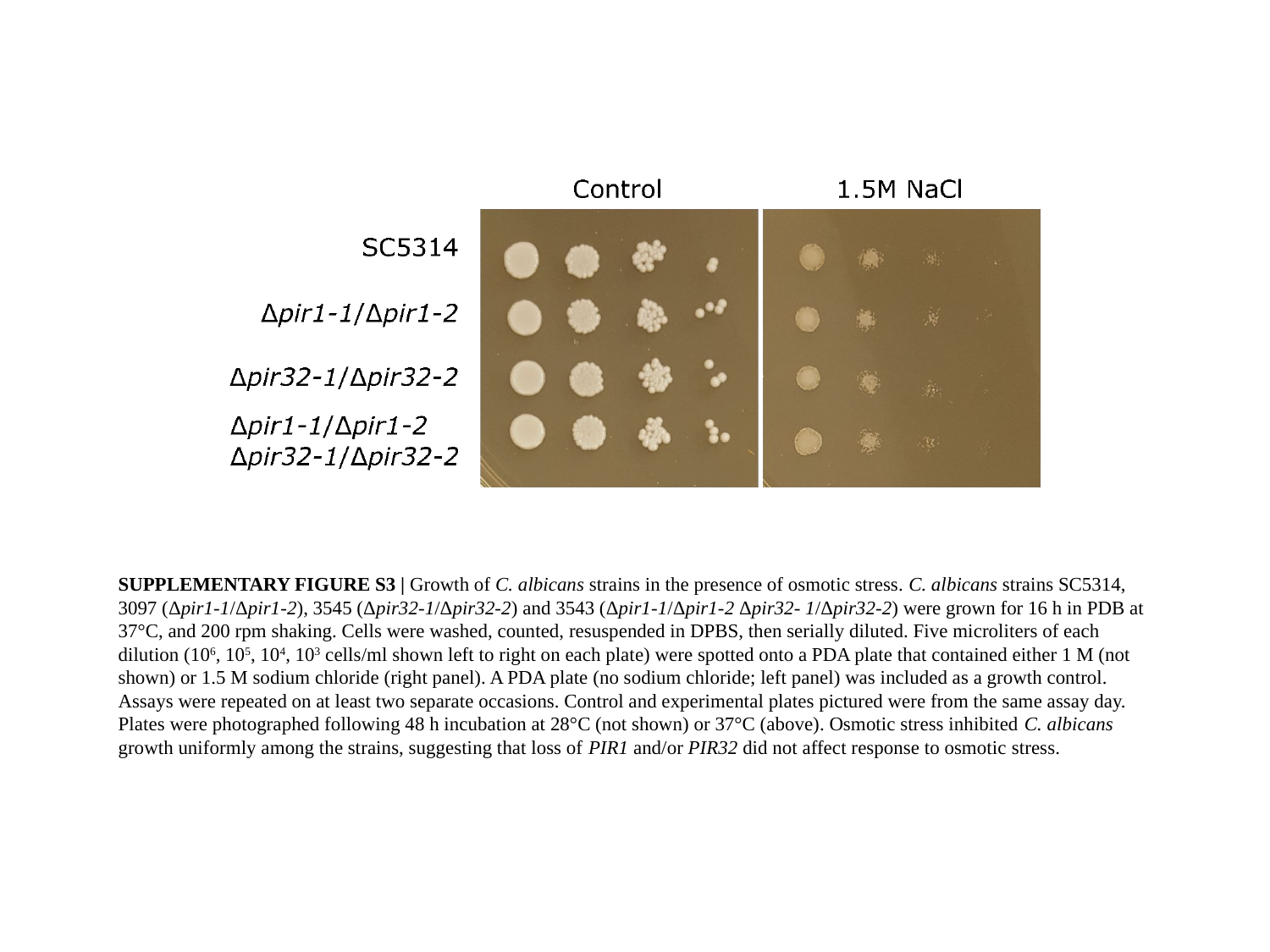

SUPPLEMENTARY FIGURE S3 | Growth of C. albicans strains in the presence of osmotic stress. C. albicans strains SC5314, 3097 (Δpir1-1/Δpir1-2), 3545 (Δpir32-1/Δpir32-2) and 3543 (Δpir1-1/Δpir1-2 Δpir32- 1/Δpir32-2) were grown for 16 h in PDB at 37°C, and 200 rpm shaking. Cells were washed, counted, resuspended in DPBS, then serially diluted. Five microliters of each dilution (106, 105, 104, 103 cells/ml shown left to right on each plate) were spotted onto a PDA plate that contained either 1 M (not shown) or 1.5 M sodium chloride (right panel). A PDA plate (no sodium chloride; left panel) was included as a growth control. Assays were repeated on at least two separate occasions. Control and experimental plates pictured were from the same assay day. Plates were photographed following 48 h incubation at 28°C (not shown) or 37°C (above). Osmotic stress inhibited C. albicans growth uniformly among the strains, suggesting that loss of PIR1 and/or PIR32 did not affect response to osmotic stress.

## Slide 4
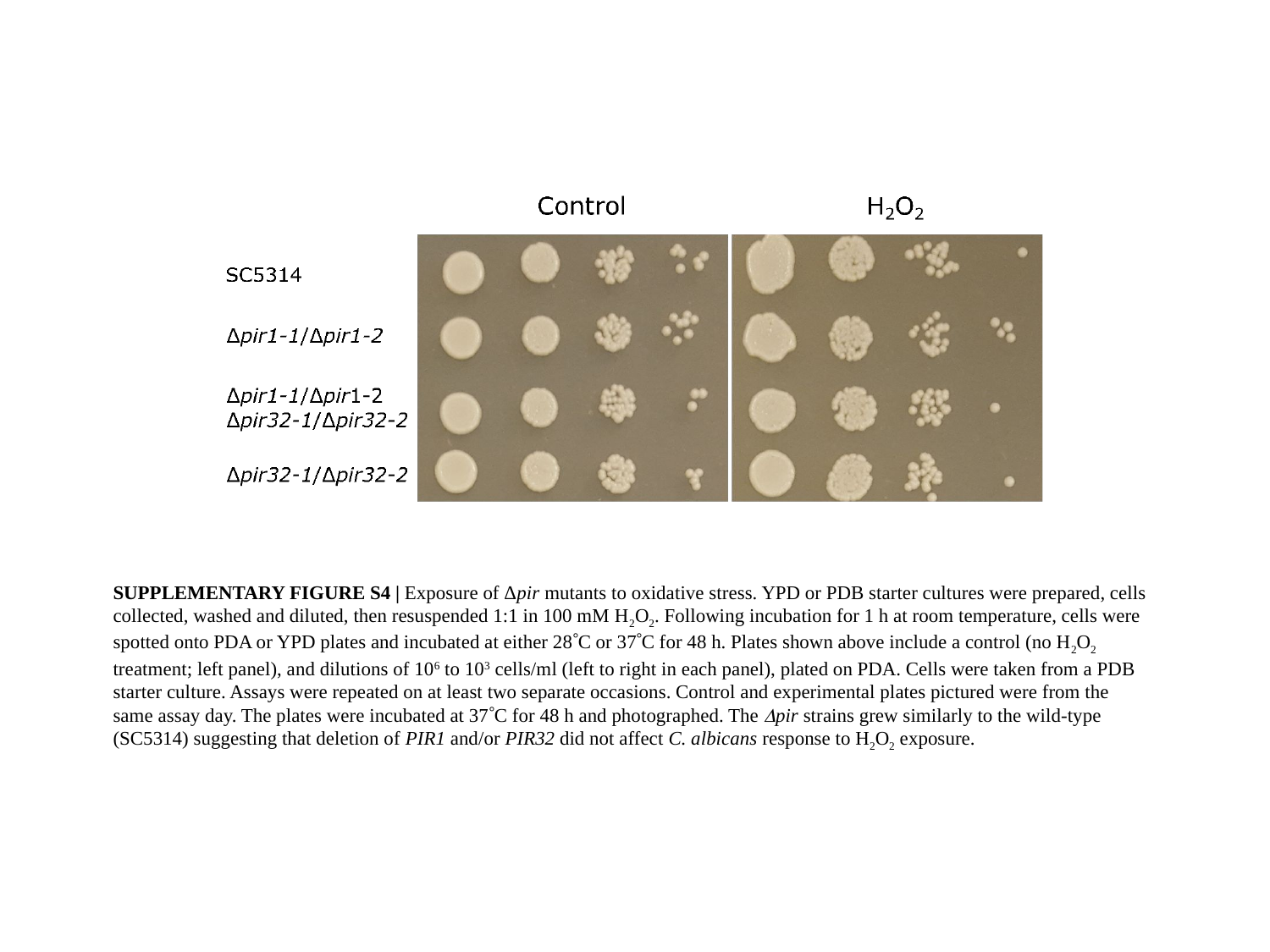

SUPPLEMENTARY FIGURE S4 | Exposure of Δpir mutants to oxidative stress. YPD or PDB starter cultures were prepared, cells collected, washed and diluted, then resuspended 1:1 in 100 mM H2O2. Following incubation for 1 h at room temperature, cells were spotted onto PDA or YPD plates and incubated at either 28°C or 37°C for 48 h. Plates shown above include a control (no H2O2 treatment; left panel), and dilutions of 106 to 103 cells/ml (left to right in each panel), plated on PDA. Cells were taken from a PDB starter culture. Assays were repeated on at least two separate occasions. Control and experimental plates pictured were from the same assay day. The plates were incubated at 37°C for 48 h and photographed. The Dpir strains grew similarly to the wild-type (SC5314) suggesting that deletion of PIR1 and/or PIR32 did not affect C. albicans response to H2O2 exposure.

## Slide 5
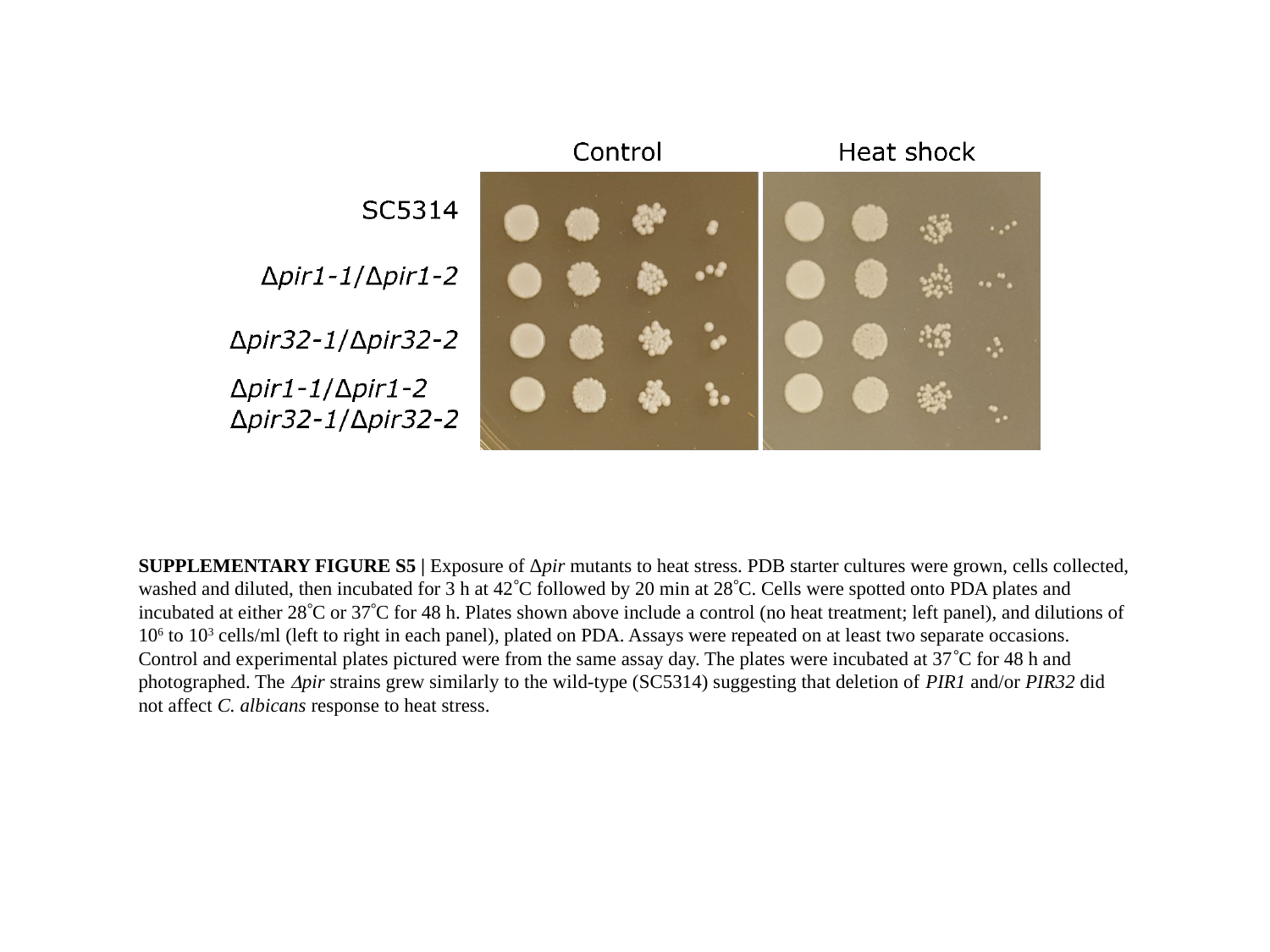

SUPPLEMENTARY FIGURE S5 | Exposure of Δpir mutants to heat stress. PDB starter cultures were grown, cells collected, washed and diluted, then incubated for 3 h at 42°C followed by 20 min at 28°C. Cells were spotted onto PDA plates and incubated at either 28°C or 37°C for 48 h. Plates shown above include a control (no heat treatment; left panel), and dilutions of 106 to 103 cells/ml (left to right in each panel), plated on PDA. Assays were repeated on at least two separate occasions. Control and experimental plates pictured were from the same assay day. The plates were incubated at 37°C for 48 h and photographed. The Dpir strains grew similarly to the wild-type (SC5314) suggesting that deletion of PIR1 and/or PIR32 did not affect C. albicans response to heat stress.

## Slide 6
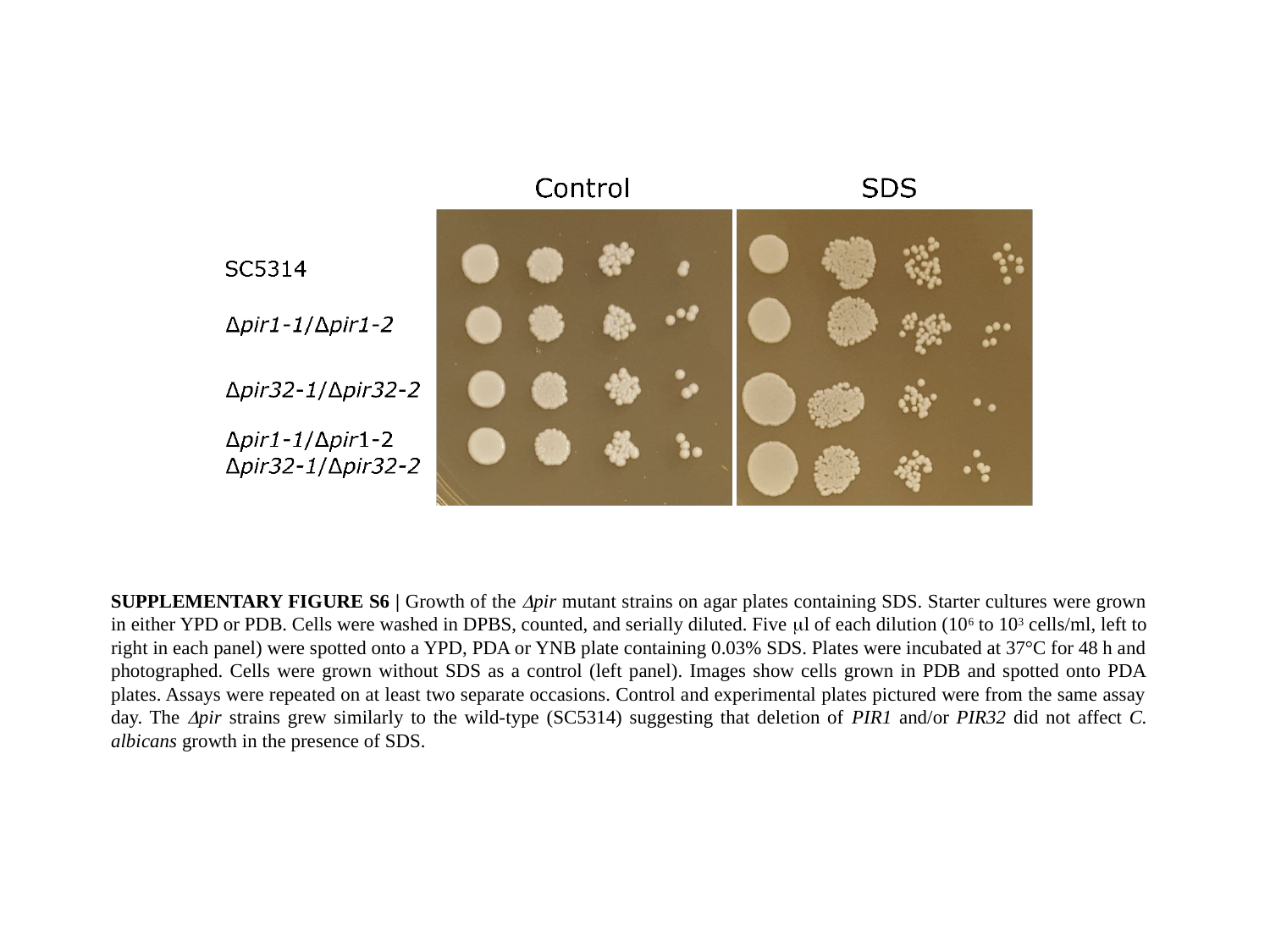

SUPPLEMENTARY FIGURE S6 | Growth of the Dpir mutant strains on agar plates containing SDS. Starter cultures were grown in either YPD or PDB. Cells were washed in DPBS, counted, and serially diluted. Five ml of each dilution (106 to 103 cells/ml, left to right in each panel) were spotted onto a YPD, PDA or YNB plate containing 0.03% SDS. Plates were incubated at 37°C for 48 h and photographed. Cells were grown without SDS as a control (left panel). Images show cells grown in PDB and spotted onto PDA plates. Assays were repeated on at least two separate occasions. Control and experimental plates pictured were from the same assay day. The Dpir strains grew similarly to the wild-type (SC5314) suggesting that deletion of PIR1 and/or PIR32 did not affect C. albicans growth in the presence of SDS.

## Slide 7
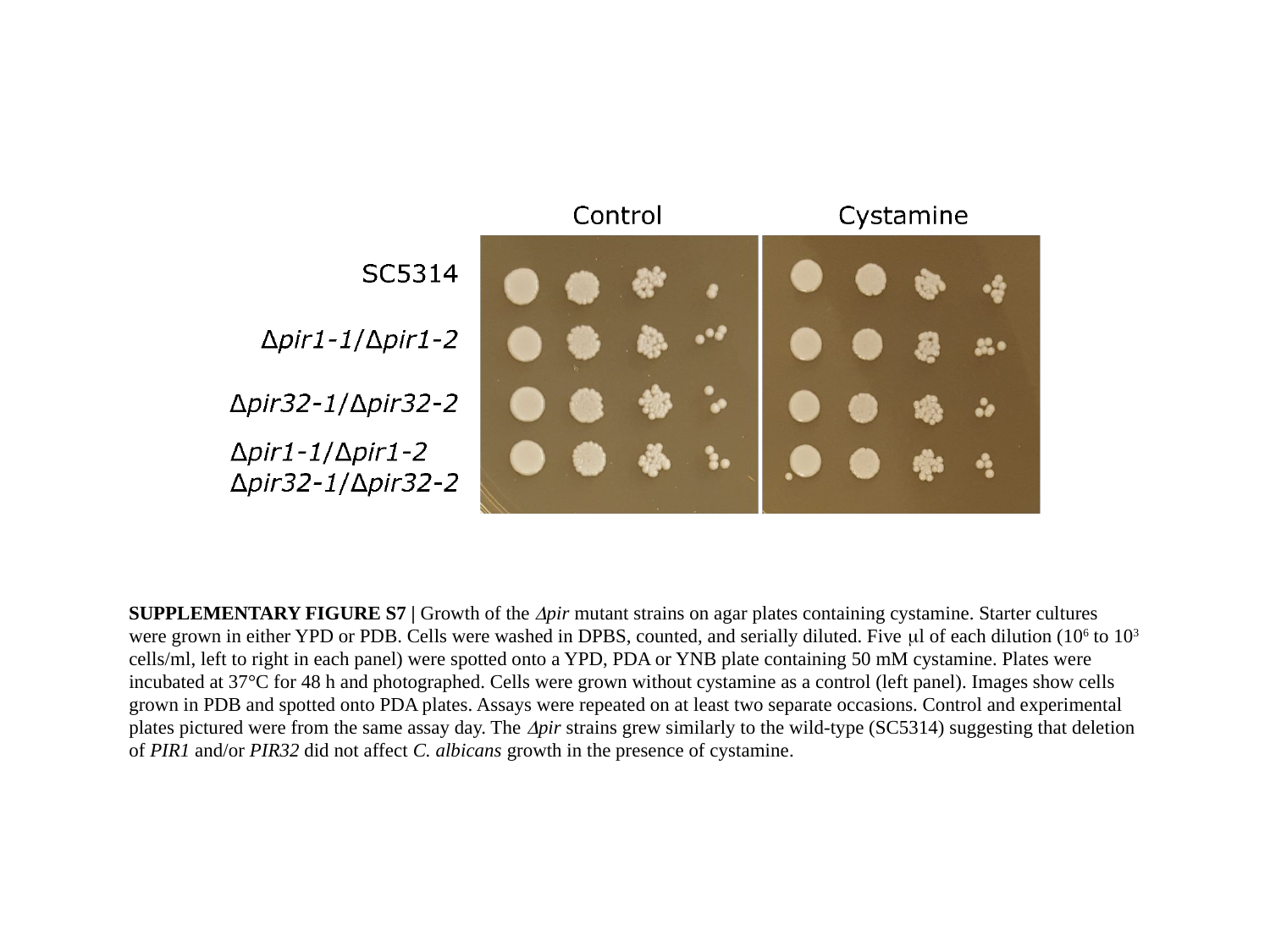

SUPPLEMENTARY FIGURE S7 | Growth of the Dpir mutant strains on agar plates containing cystamine. Starter cultures were grown in either YPD or PDB. Cells were washed in DPBS, counted, and serially diluted. Five ml of each dilution (106 to 103 cells/ml, left to right in each panel) were spotted onto a YPD, PDA or YNB plate containing 50 mM cystamine. Plates were incubated at 37°C for 48 h and photographed. Cells were grown without cystamine as a control (left panel). Images show cells grown in PDB and spotted onto PDA plates. Assays were repeated on at least two separate occasions. Control and experimental plates pictured were from the same assay day. The Dpir strains grew similarly to the wild-type (SC5314) suggesting that deletion of PIR1 and/or PIR32 did not affect C. albicans growth in the presence of cystamine.

## Slide 8
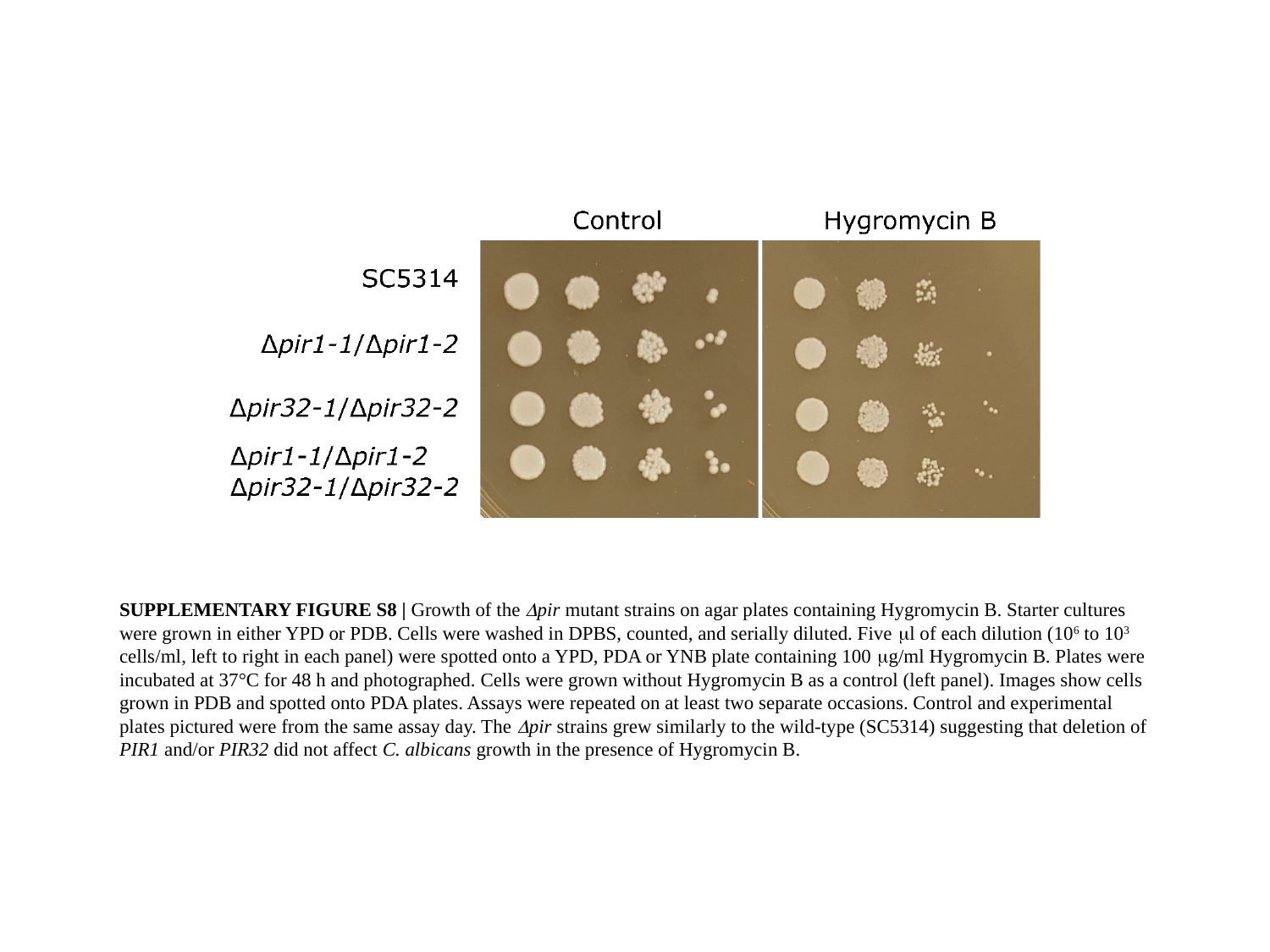

SUPPLEMENTARY FIGURE S8 | Growth of the Dpir mutant strains on agar plates containing Hygromycin B. Starter cultures were grown in either YPD or PDB. Cells were washed in DPBS, counted, and serially diluted. Five ml of each dilution (106 to 103 cells/ml, left to right in each panel) were spotted onto a YPD, PDA or YNB plate containing 100 mg/ml Hygromycin B. Plates were incubated at 37°C for 48 h and photographed. Cells were grown without Hygromycin B as a control (left panel). Images show cells grown in PDB and spotted onto PDA plates. Assays were repeated on at least two separate occasions. Control and experimental plates pictured were from the same assay day. The Dpir strains grew similarly to the wild-type (SC5314) suggesting that deletion of PIR1 and/or PIR32 did not affect C. albicans growth in the presence of Hygromycin B.

## Slide 9
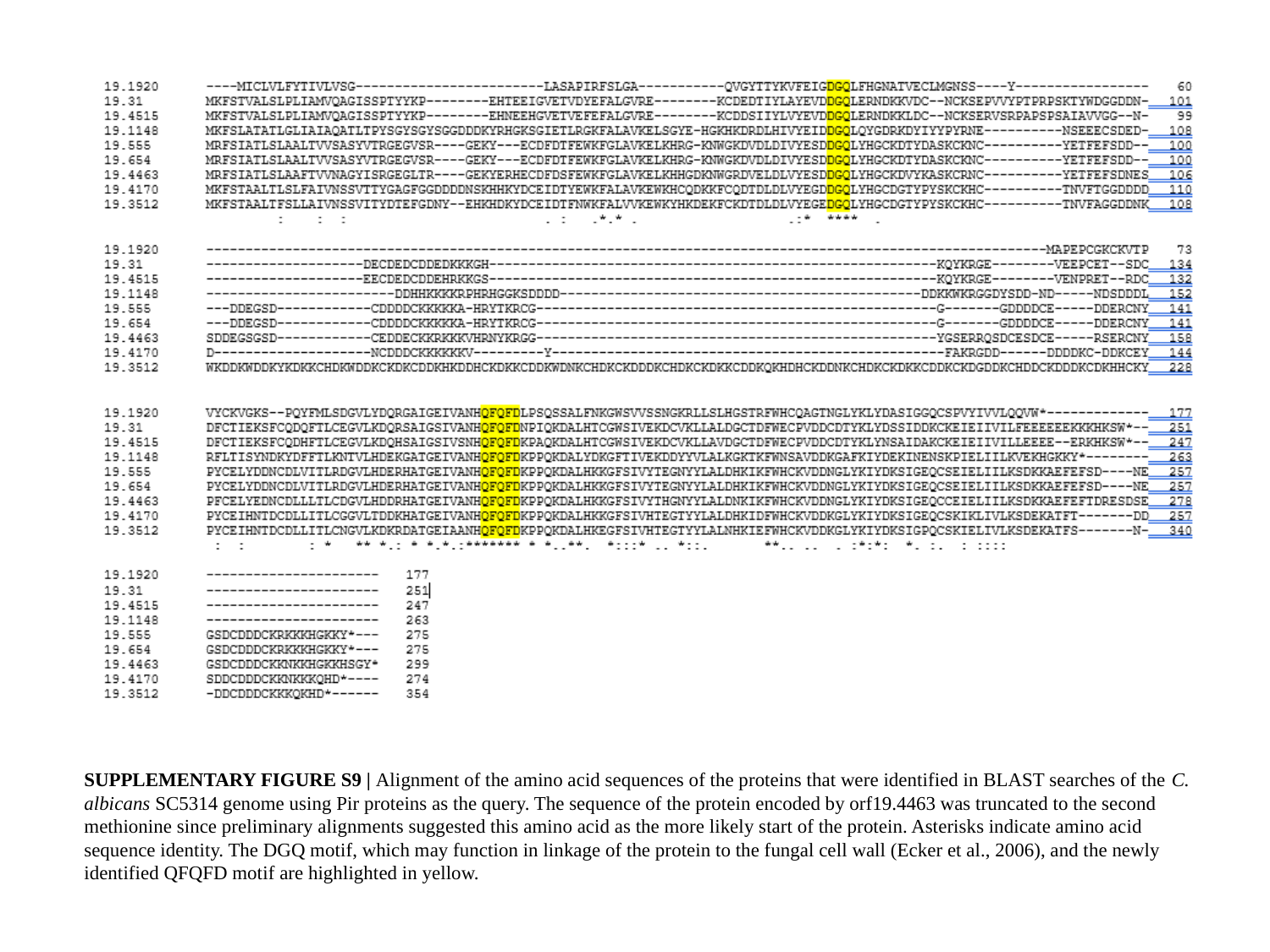

SUPPLEMENTARY FIGURE S9 | Alignment of the amino acid sequences of the proteins that were identified in BLAST searches of the C. albicans SC5314 genome using Pir proteins as the query. The sequence of the protein encoded by orf19.4463 was truncated to the second methionine since preliminary alignments suggested this amino acid as the more likely start of the protein. Asterisks indicate amino acid sequence identity. The DGQ motif, which may function in linkage of the protein to the fungal cell wall (Ecker et al., 2006), and the newly identified QFQFD motif are highlighted in yellow.

## Slide 10
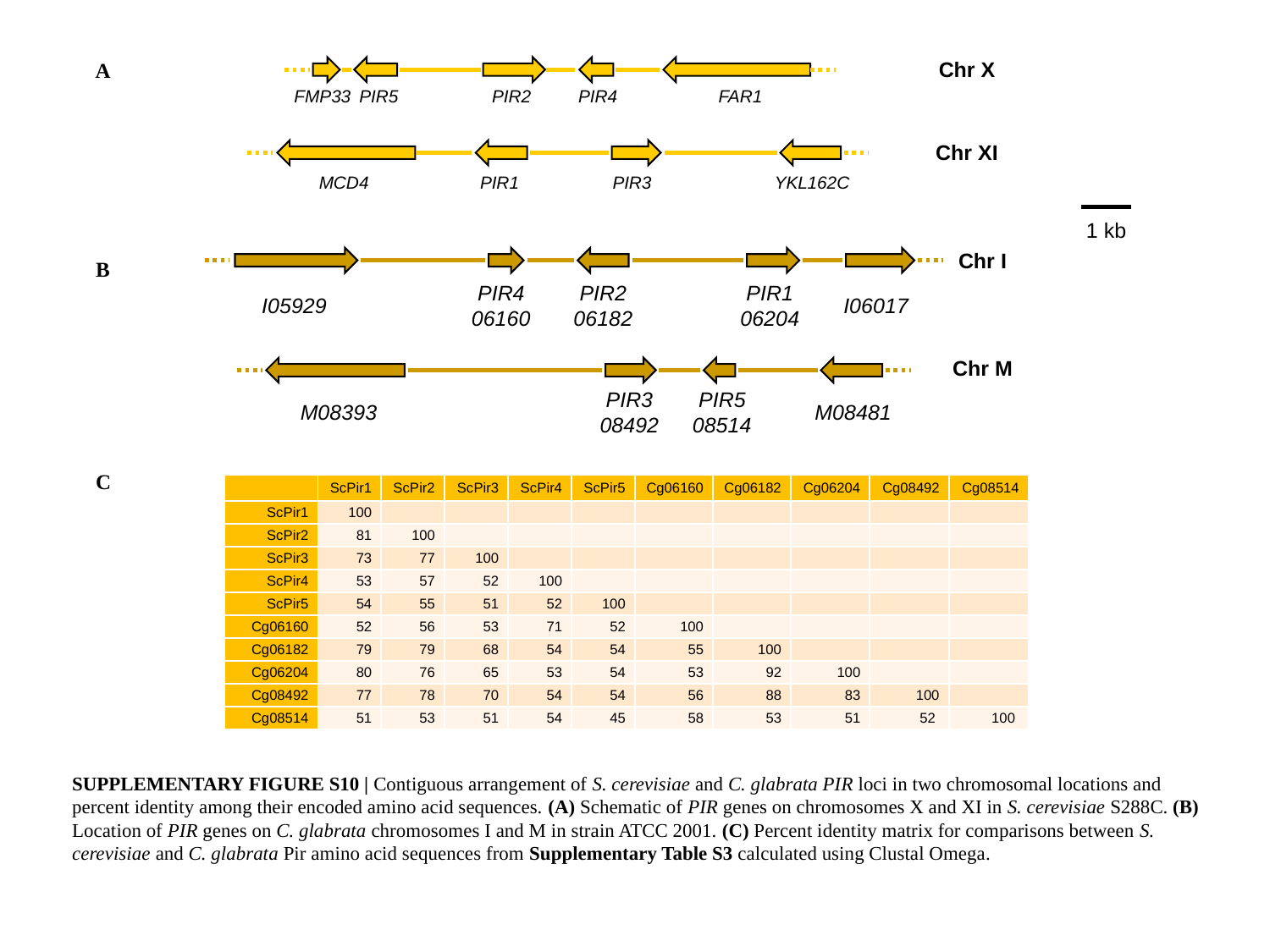

Chr X
FMP33
PIR5
PIR2
PIR4
FAR1
Chr XI
MCD4
PIR1
PIR3
YKL162C
A
1 kb
Chr I
PIR4
06160
PIR2
06182
PIR1
06204
I05929
I06017
Chr M
PIR3
08492
PIR5
08514
M08393
M08481
B
C
| | ScPir1 | ScPir2 | ScPir3 | ScPir4 | ScPir5 | Cg06160 | Cg06182 | Cg06204 | Cg08492 | Cg08514 |
| --- | --- | --- | --- | --- | --- | --- | --- | --- | --- | --- |
| ScPir1 | 100 | | | | | | | | | |
| ScPir2 | 81 | 100 | | | | | | | | |
| ScPir3 | 73 | 77 | 100 | | | | | | | |
| ScPir4 | 53 | 57 | 52 | 100 | | | | | | |
| ScPir5 | 54 | 55 | 51 | 52 | 100 | | | | | |
| Cg06160 | 52 | 56 | 53 | 71 | 52 | 100 | | | | |
| Cg06182 | 79 | 79 | 68 | 54 | 54 | 55 | 100 | | | |
| Cg06204 | 80 | 76 | 65 | 53 | 54 | 53 | 92 | 100 | | |
| Cg08492 | 77 | 78 | 70 | 54 | 54 | 56 | 88 | 83 | 100 | |
| Cg08514 | 51 | 53 | 51 | 54 | 45 | 58 | 53 | 51 | 52 | 100 |
SUPPLEMENTARY FIGURE S10 | Contiguous arrangement of S. cerevisiae and C. glabrata PIR loci in two chromosomal locations and percent identity among their encoded amino acid sequences. (A) Schematic of PIR genes on chromosomes X and XI in S. cerevisiae S288C. (B) Location of PIR genes on C. glabrata chromosomes I and M in strain ATCC 2001. (C) Percent identity matrix for comparisons between S. cerevisiae and C. glabrata Pir amino acid sequences from Supplementary Table S3 calculated using Clustal Omega.

## Slide 11
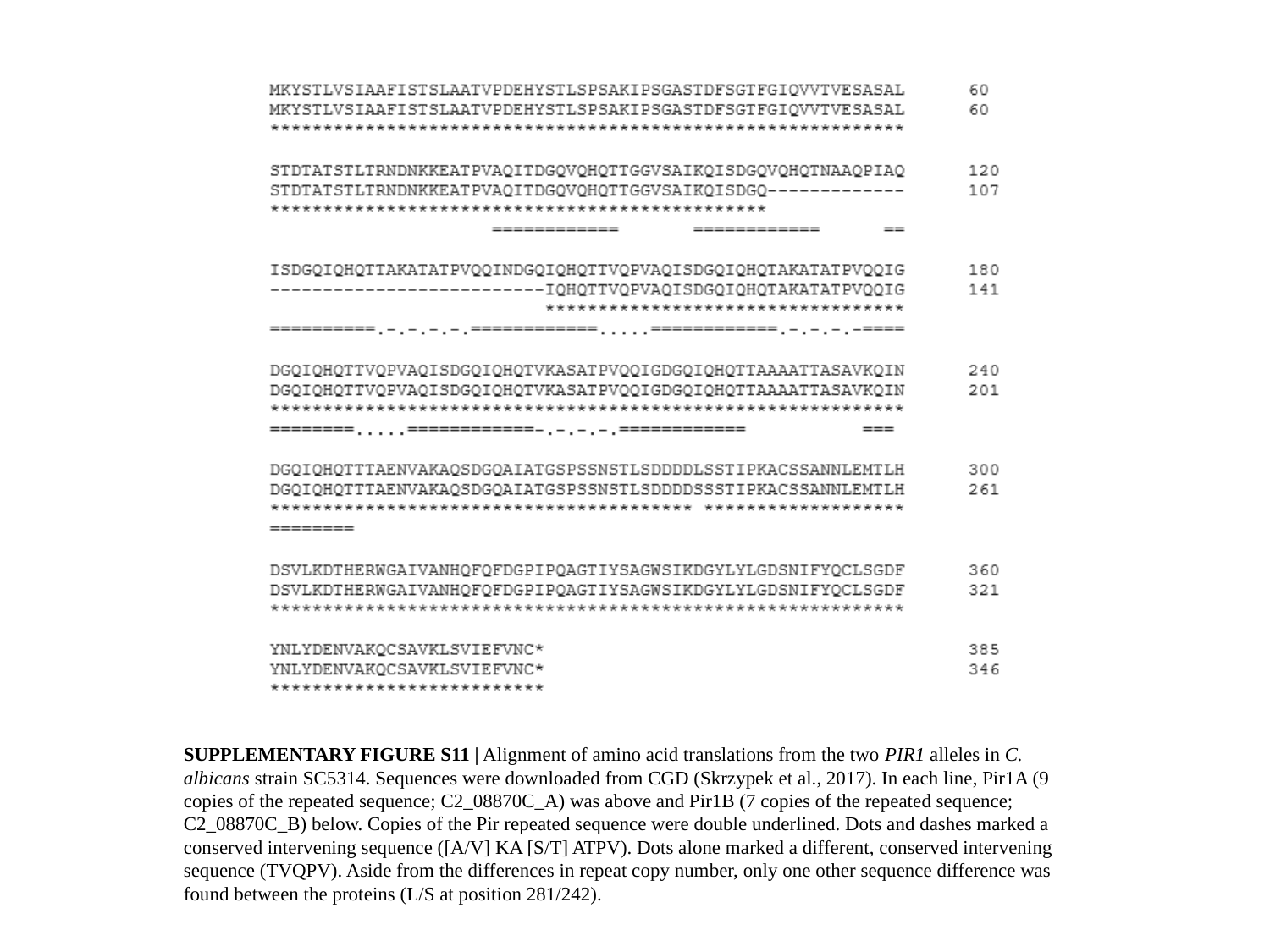

SUPPLEMENTARY FIGURE S11 | Alignment of amino acid translations from the two PIR1 alleles in C. albicans strain SC5314. Sequences were downloaded from CGD (Skrzypek et al., 2017). In each line, Pir1A (9 copies of the repeated sequence; C2_08870C_A) was above and Pir1B (7 copies of the repeated sequence; C2_08870C_B) below. Copies of the Pir repeated sequence were double underlined. Dots and dashes marked a conserved intervening sequence ([A/V] KA [S/T] ATPV). Dots alone marked a different, conserved intervening sequence (TVQPV). Aside from the differences in repeat copy number, only one other sequence difference was found between the proteins (L/S at position 281/242).

## Slide 12
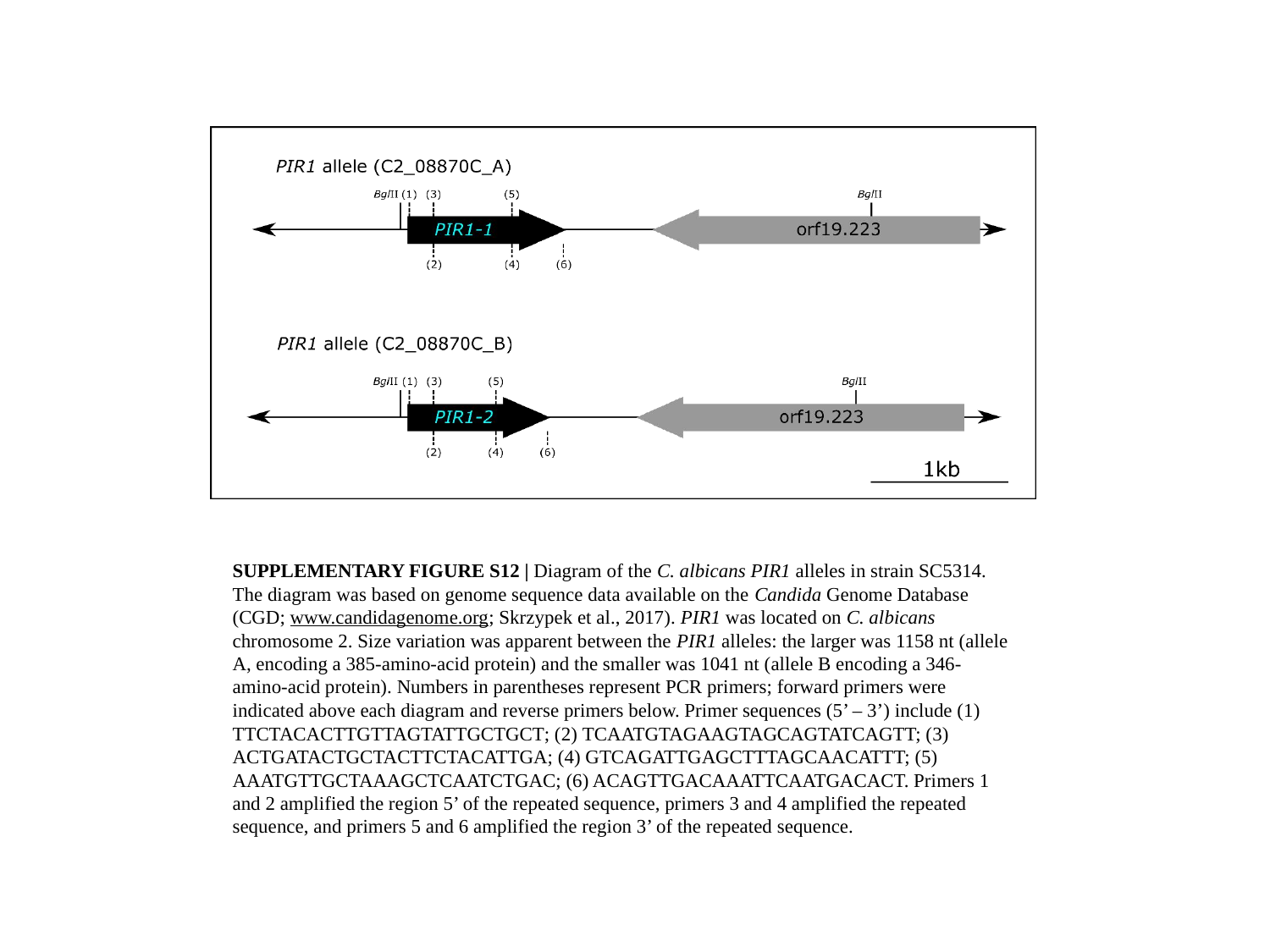

SUPPLEMENTARY FIGURE S12 | Diagram of the C. albicans PIR1 alleles in strain SC5314. The diagram was based on genome sequence data available on the Candida Genome Database (CGD; www.candidagenome.org; Skrzypek et al., 2017). PIR1 was located on C. albicans chromosome 2. Size variation was apparent between the PIR1 alleles: the larger was 1158 nt (allele A, encoding a 385-amino-acid protein) and the smaller was 1041 nt (allele B encoding a 346-amino-acid protein). Numbers in parentheses represent PCR primers; forward primers were indicated above each diagram and reverse primers below. Primer sequences (5’ – 3’) include (1) TTCTACACTTGTTAGTATTGCTGCT; (2) TCAATGTAGAAGTAGCAGTATCAGTT; (3) ACTGATACTGCTACTTCTACATTGA; (4) GTCAGATTGAGCTTTAGCAACATTT; (5) AAATGTTGCTAAAGCTCAATCTGAC; (6) ACAGTTGACAAATTCAATGACACT. Primers 1 and 2 amplified the region 5’ of the repeated sequence, primers 3 and 4 amplified the repeated sequence, and primers 5 and 6 amplified the region 3’ of the repeated sequence.

## Slide 13
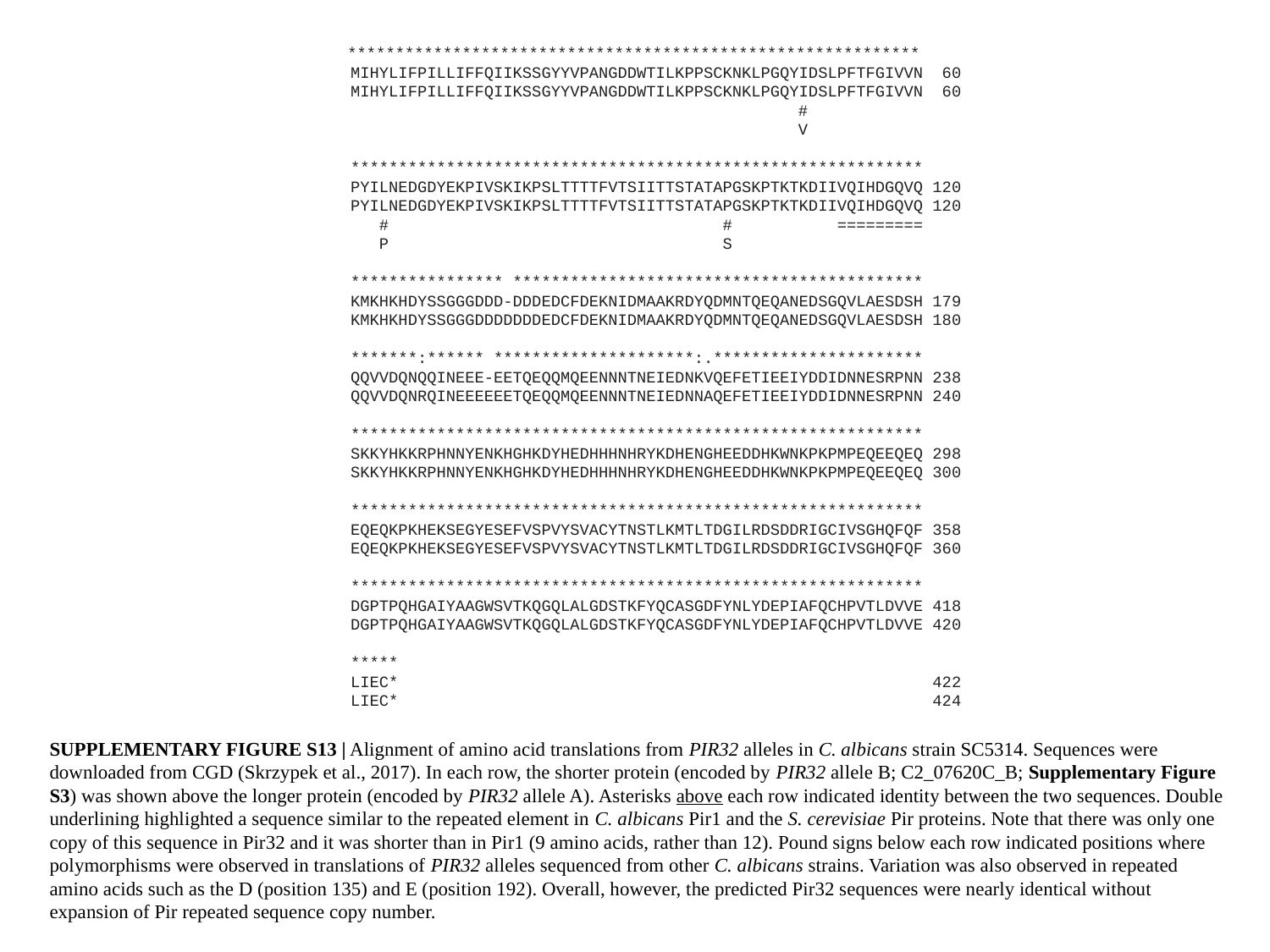

************************************************************
	MIHYLIFPILLIFFQIIKSSGYYVPANGDDWTILKPPSCKNKLPGQYIDSLPFTFGIVVN 60
	MIHYLIFPILLIFFQIIKSSGYYVPANGDDWTILKPPSCKNKLPGQYIDSLPFTFGIVVN 60
	 #
	 V
	************************************************************
	PYILNEDGDYEKPIVSKIKPSLTTTTFVTSIITTSTATAPGSKPTKTKDIIVQIHDGQVQ 120
	PYILNEDGDYEKPIVSKIKPSLTTTTFVTSIITTSTATAPGSKPTKTKDIIVQIHDGQVQ 120
	 # # =========
	 P S
	**************** *******************************************
	KMKHKHDYSSGGGDDD-DDDEDCFDEKNIDMAAKRDYQDMNTQEQANEDSGQVLAESDSH 179
	KMKHKHDYSSGGGDDDDDDDEDCFDEKNIDMAAKRDYQDMNTQEQANEDSGQVLAESDSH 180
	*******:****** *********************:.**********************
	QQVVDQNQQINEEE-EETQEQQMQEENNNTNEIEDNKVQEFETIEEIYDDIDNNESRPNN 238
	QQVVDQNRQINEEEEEETQEQQMQEENNNTNEIEDNNAQEFETIEEIYDDIDNNESRPNN 240
	************************************************************
	SKKYHKKRPHNNYENKHGHKDYHEDHHHNHRYKDHENGHEEDDHKWNKPKPMPEQEEQEQ 298
	SKKYHKKRPHNNYENKHGHKDYHEDHHHNHRYKDHENGHEEDDHKWNKPKPMPEQEEQEQ 300
	************************************************************
	EQEQKPKHEKSEGYESEFVSPVYSVACYTNSTLKMTLTDGILRDSDDRIGCIVSGHQFQF 358
	EQEQKPKHEKSEGYESEFVSPVYSVACYTNSTLKMTLTDGILRDSDDRIGCIVSGHQFQF 360
	************************************************************
	DGPTPQHGAIYAAGWSVTKQGQLALGDSTKFYQCASGDFYNLYDEPIAFQCHPVTLDVVE 418
	DGPTPQHGAIYAAGWSVTKQGQLALGDSTKFYQCASGDFYNLYDEPIAFQCHPVTLDVVE 420
	*****
	LIEC* 422
	LIEC* 424
SUPPLEMENTARY FIGURE S13 | Alignment of amino acid translations from PIR32 alleles in C. albicans strain SC5314. Sequences were downloaded from CGD (Skrzypek et al., 2017). In each row, the shorter protein (encoded by PIR32 allele B; C2_07620C_B; Supplementary Figure S3) was shown above the longer protein (encoded by PIR32 allele A). Asterisks above each row indicated identity between the two sequences. Double underlining highlighted a sequence similar to the repeated element in C. albicans Pir1 and the S. cerevisiae Pir proteins. Note that there was only one copy of this sequence in Pir32 and it was shorter than in Pir1 (9 amino acids, rather than 12). Pound signs below each row indicated positions where polymorphisms were observed in translations of PIR32 alleles sequenced from other C. albicans strains. Variation was also observed in repeated amino acids such as the D (position 135) and E (position 192). Overall, however, the predicted Pir32 sequences were nearly identical without expansion of Pir repeated sequence copy number.

## Slide 14
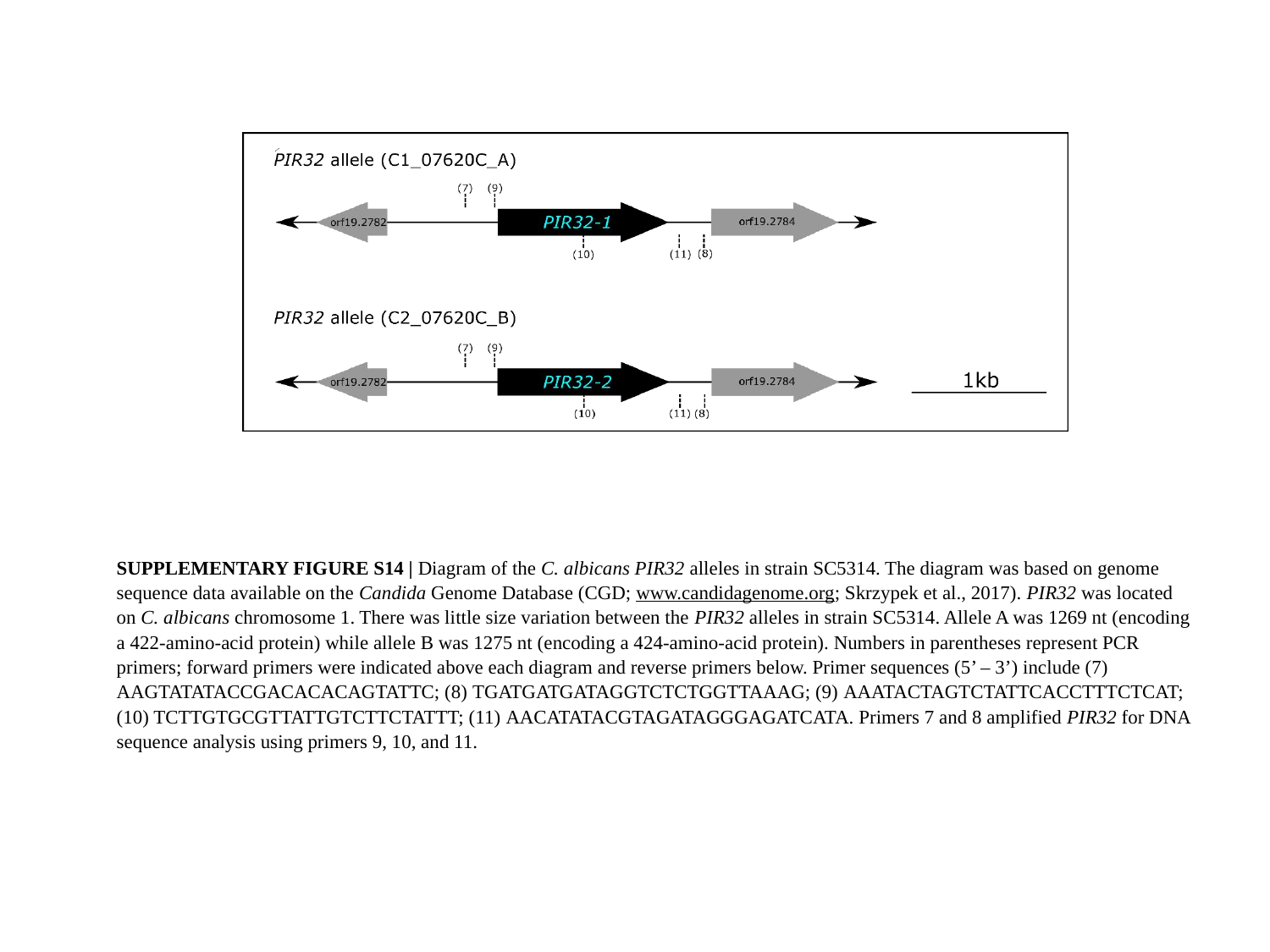

SUPPLEMENTARY FIGURE S14 | Diagram of the C. albicans PIR32 alleles in strain SC5314. The diagram was based on genome sequence data available on the Candida Genome Database (CGD; www.candidagenome.org; Skrzypek et al., 2017). PIR32 was located on C. albicans chromosome 1. There was little size variation between the PIR32 alleles in strain SC5314. Allele A was 1269 nt (encoding a 422-amino-acid protein) while allele B was 1275 nt (encoding a 424-amino-acid protein). Numbers in parentheses represent PCR primers; forward primers were indicated above each diagram and reverse primers below. Primer sequences (5’ – 3’) include (7) aagtatataccgacacacagtattc; (8) tgatgatgataggtctctggttaaag; (9) AAATACTAGTCTATTCACCTTTCTCAT; (10) TCTTGTGCGTTATTGTCTTCTATTT; (11) Aacatatacgtagatagggagatcata. Primers 7 and 8 amplified PIR32 for DNA sequence analysis using primers 9, 10, and 11.

## Slide 15
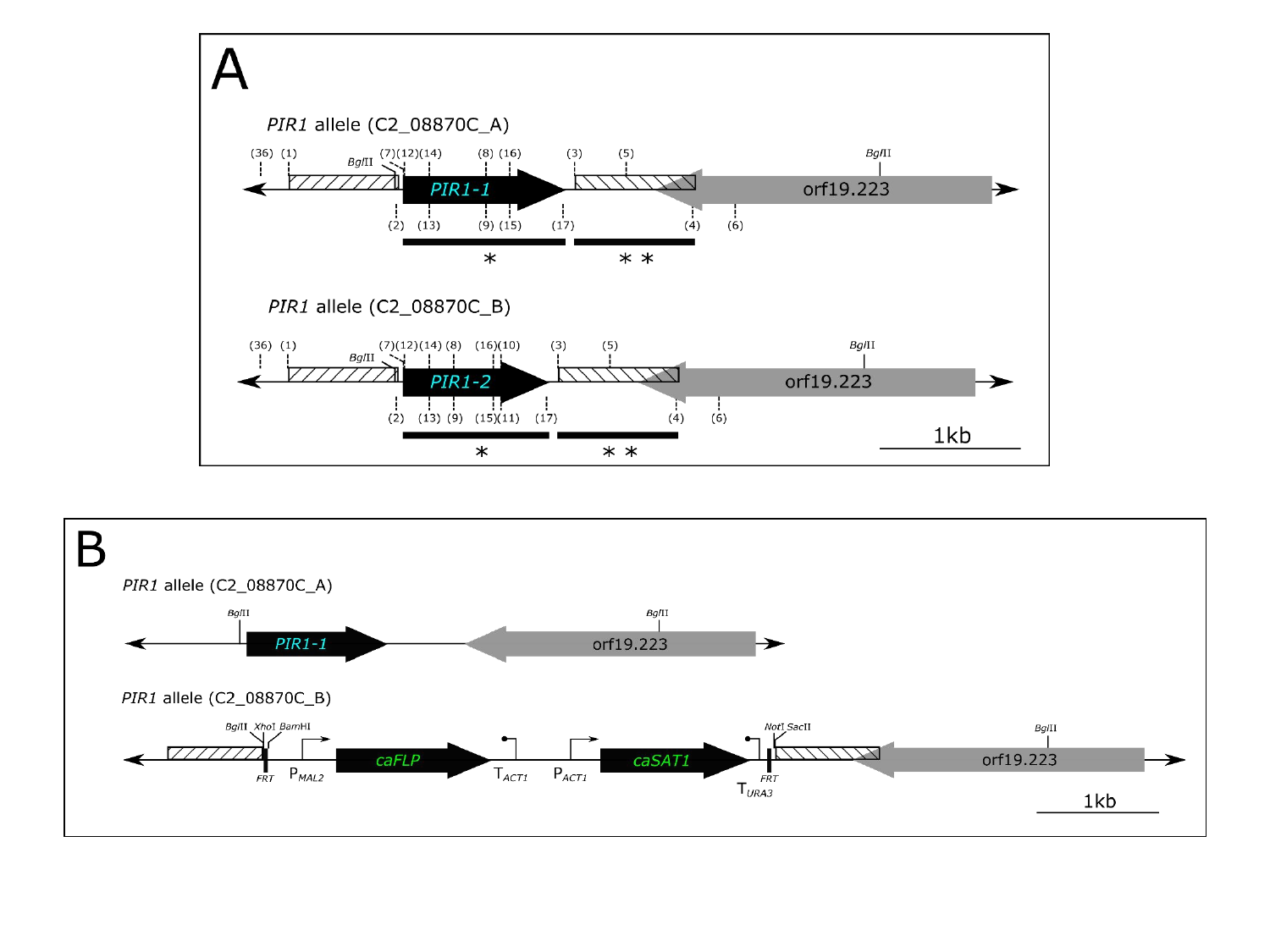

## Slide 16
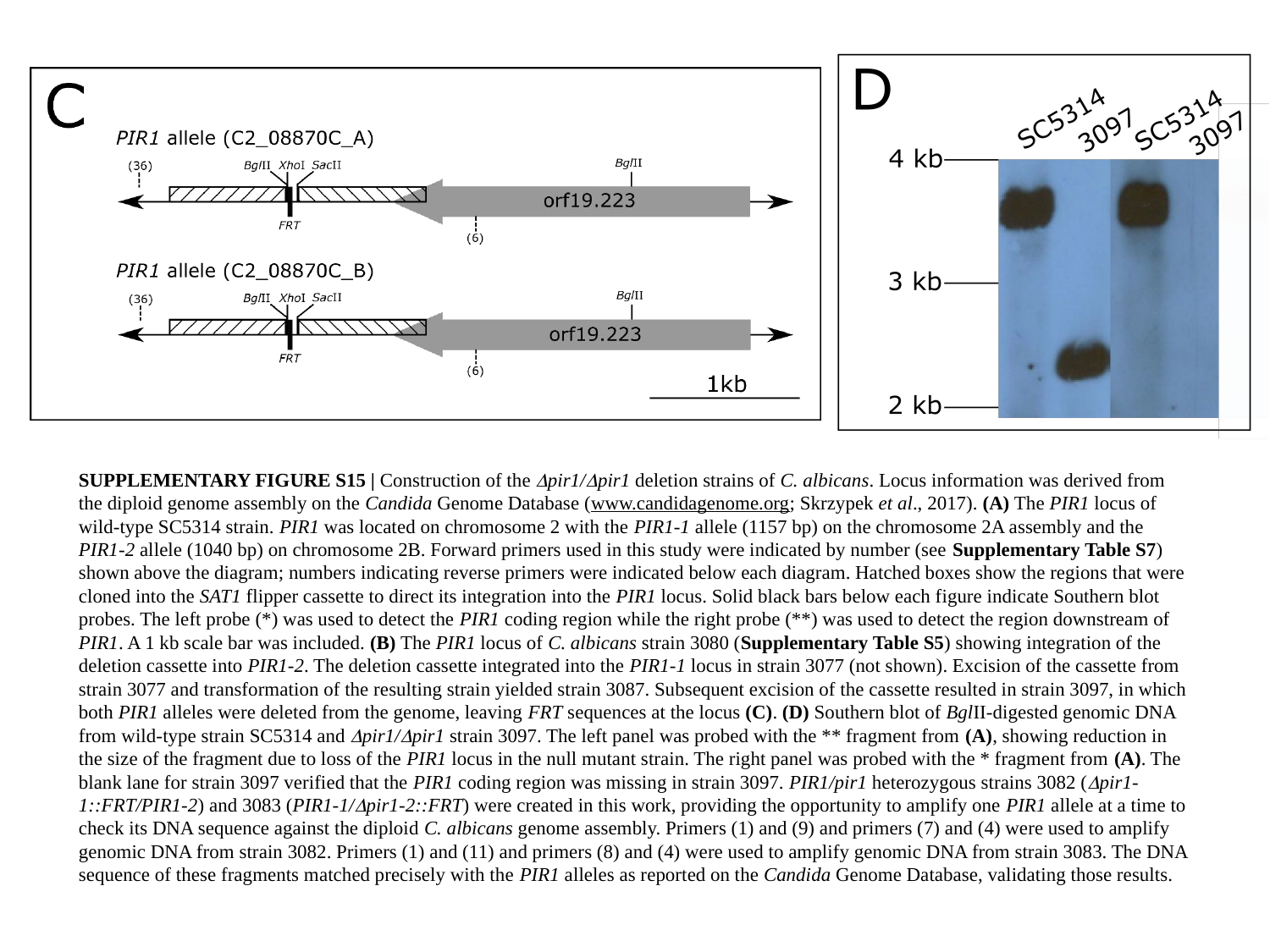

SUPPLEMENTARY FIGURE S15 | Construction of the Dpir1/Dpir1 deletion strains of C. albicans. Locus information was derived from the diploid genome assembly on the Candida Genome Database (www.candidagenome.org; Skrzypek et al., 2017). (A) The PIR1 locus of wild-type SC5314 strain. PIR1 was located on chromosome 2 with the PIR1-1 allele (1157 bp) on the chromosome 2A assembly and the PIR1-2 allele (1040 bp) on chromosome 2B. Forward primers used in this study were indicated by number (see Supplementary Table S7) shown above the diagram; numbers indicating reverse primers were indicated below each diagram. Hatched boxes show the regions that were cloned into the SAT1 flipper cassette to direct its integration into the PIR1 locus. Solid black bars below each figure indicate Southern blot probes. The left probe (*) was used to detect the PIR1 coding region while the right probe (**) was used to detect the region downstream of PIR1. A 1 kb scale bar was included. (B) The PIR1 locus of C. albicans strain 3080 (Supplementary Table S5) showing integration of the deletion cassette into PIR1-2. The deletion cassette integrated into the PIR1-1 locus in strain 3077 (not shown). Excision of the cassette from strain 3077 and transformation of the resulting strain yielded strain 3087. Subsequent excision of the cassette resulted in strain 3097, in which both PIR1 alleles were deleted from the genome, leaving FRT sequences at the locus (C). (D) Southern blot of BglII-digested genomic DNA from wild-type strain SC5314 and Dpir1/Dpir1 strain 3097. The left panel was probed with the ** fragment from (A), showing reduction in the size of the fragment due to loss of the PIR1 locus in the null mutant strain. The right panel was probed with the * fragment from (A). The blank lane for strain 3097 verified that the PIR1 coding region was missing in strain 3097. PIR1/pir1 heterozygous strains 3082 (Dpir1-1::FRT/PIR1-2) and 3083 (PIR1-1/Dpir1-2::FRT) were created in this work, providing the opportunity to amplify one PIR1 allele at a time to check its DNA sequence against the diploid C. albicans genome assembly. Primers (1) and (9) and primers (7) and (4) were used to amplify genomic DNA from strain 3082. Primers (1) and (11) and primers (8) and (4) were used to amplify genomic DNA from strain 3083. The DNA sequence of these fragments matched precisely with the PIR1 alleles as reported on the Candida Genome Database, validating those results.

## Slide 17
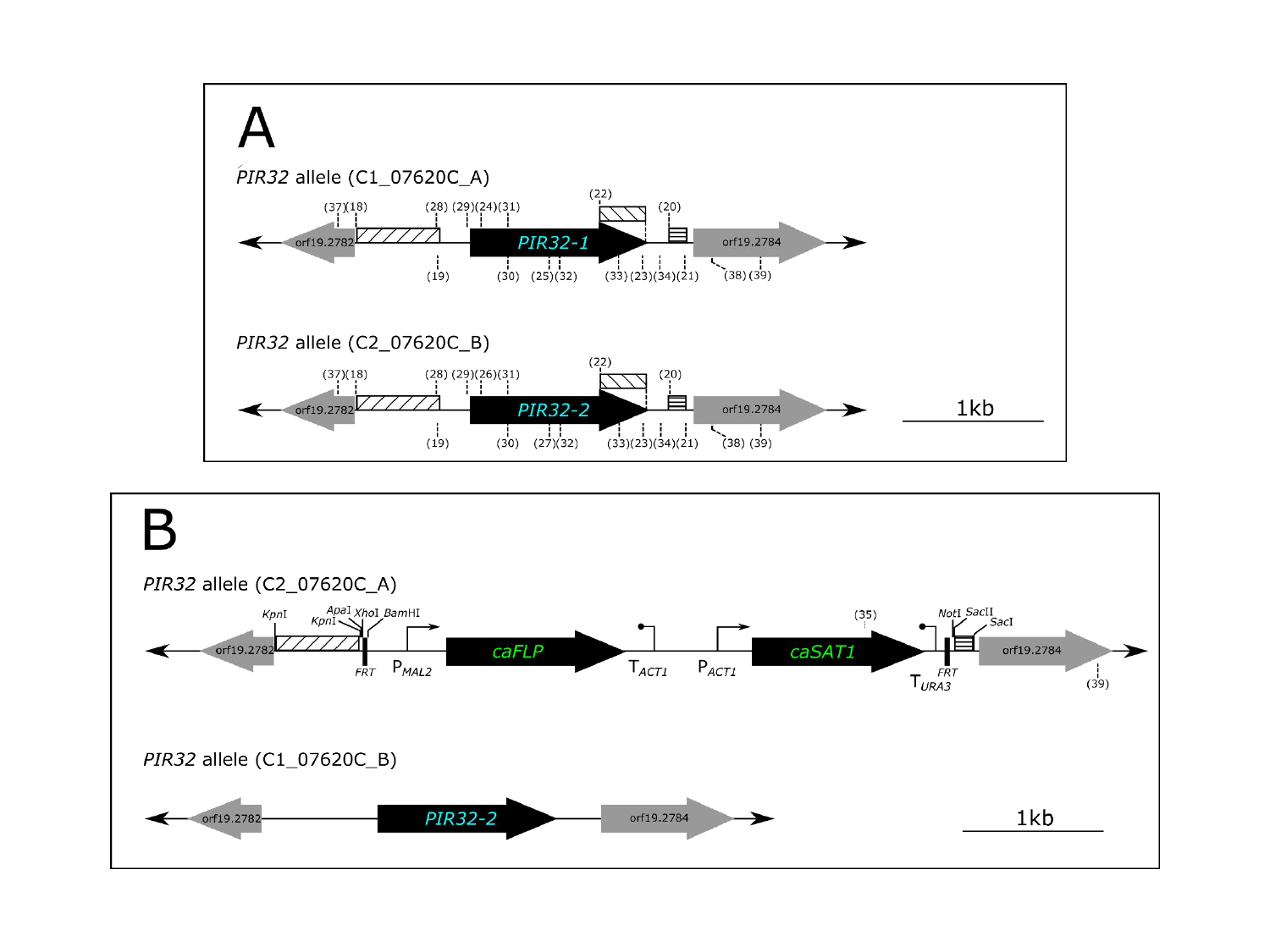

## Slide 18
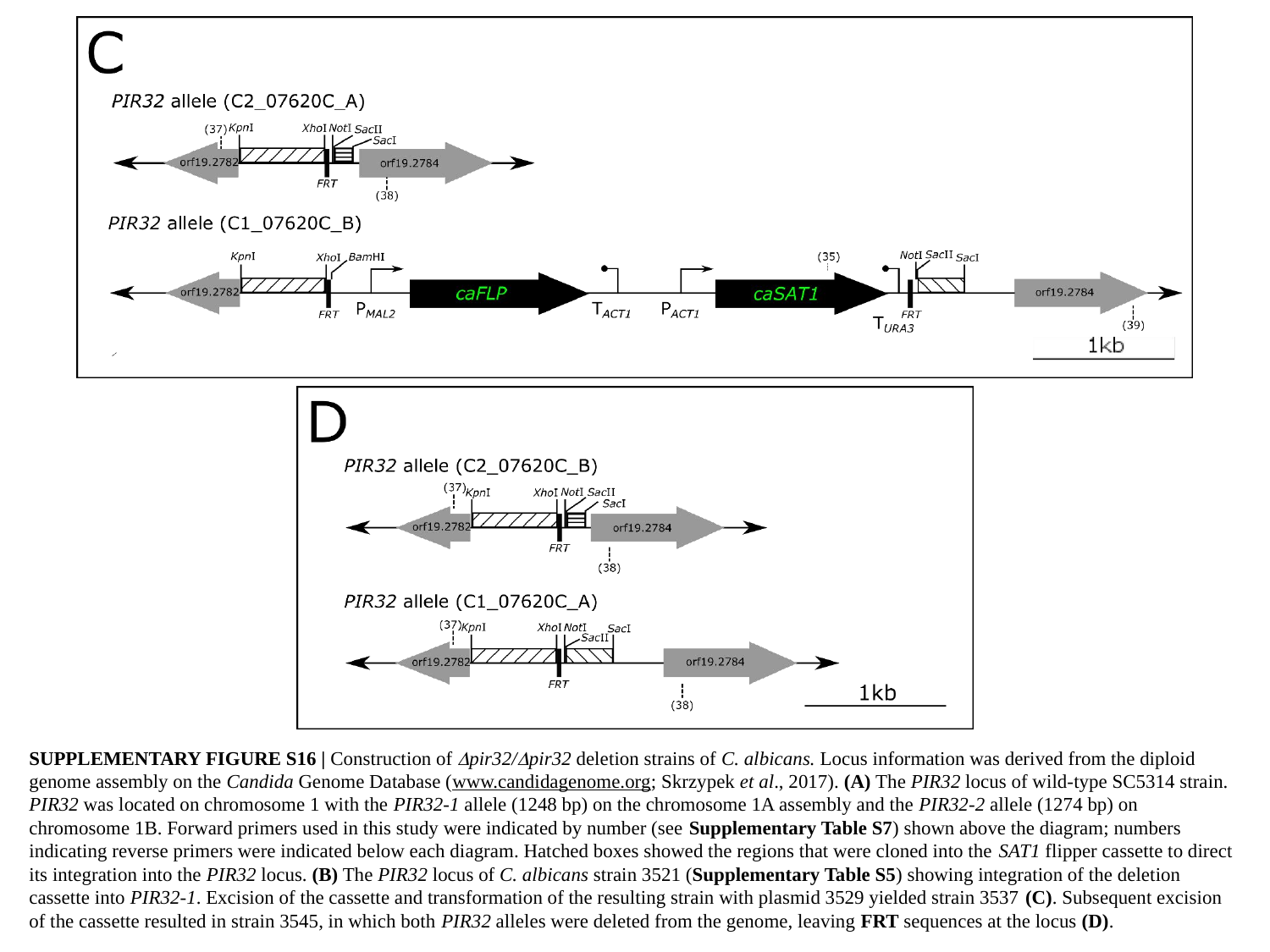

SUPPLEMENTARY FIGURE S16 | Construction of Dpir32/Dpir32 deletion strains of C. albicans. Locus information was derived from the diploid genome assembly on the Candida Genome Database (www.candidagenome.org; Skrzypek et al., 2017). (A) The PIR32 locus of wild-type SC5314 strain. PIR32 was located on chromosome 1 with the PIR32-1 allele (1248 bp) on the chromosome 1A assembly and the PIR32-2 allele (1274 bp) on chromosome 1B. Forward primers used in this study were indicated by number (see Supplementary Table S7) shown above the diagram; numbers indicating reverse primers were indicated below each diagram. Hatched boxes showed the regions that were cloned into the SAT1 flipper cassette to direct its integration into the PIR32 locus. (B) The PIR32 locus of C. albicans strain 3521 (Supplementary Table S5) showing integration of the deletion cassette into PIR32-1. Excision of the cassette and transformation of the resulting strain with plasmid 3529 yielded strain 3537 (C). Subsequent excision of the cassette resulted in strain 3545, in which both PIR32 alleles were deleted from the genome, leaving FRT sequences at the locus (D).

## Slide 19
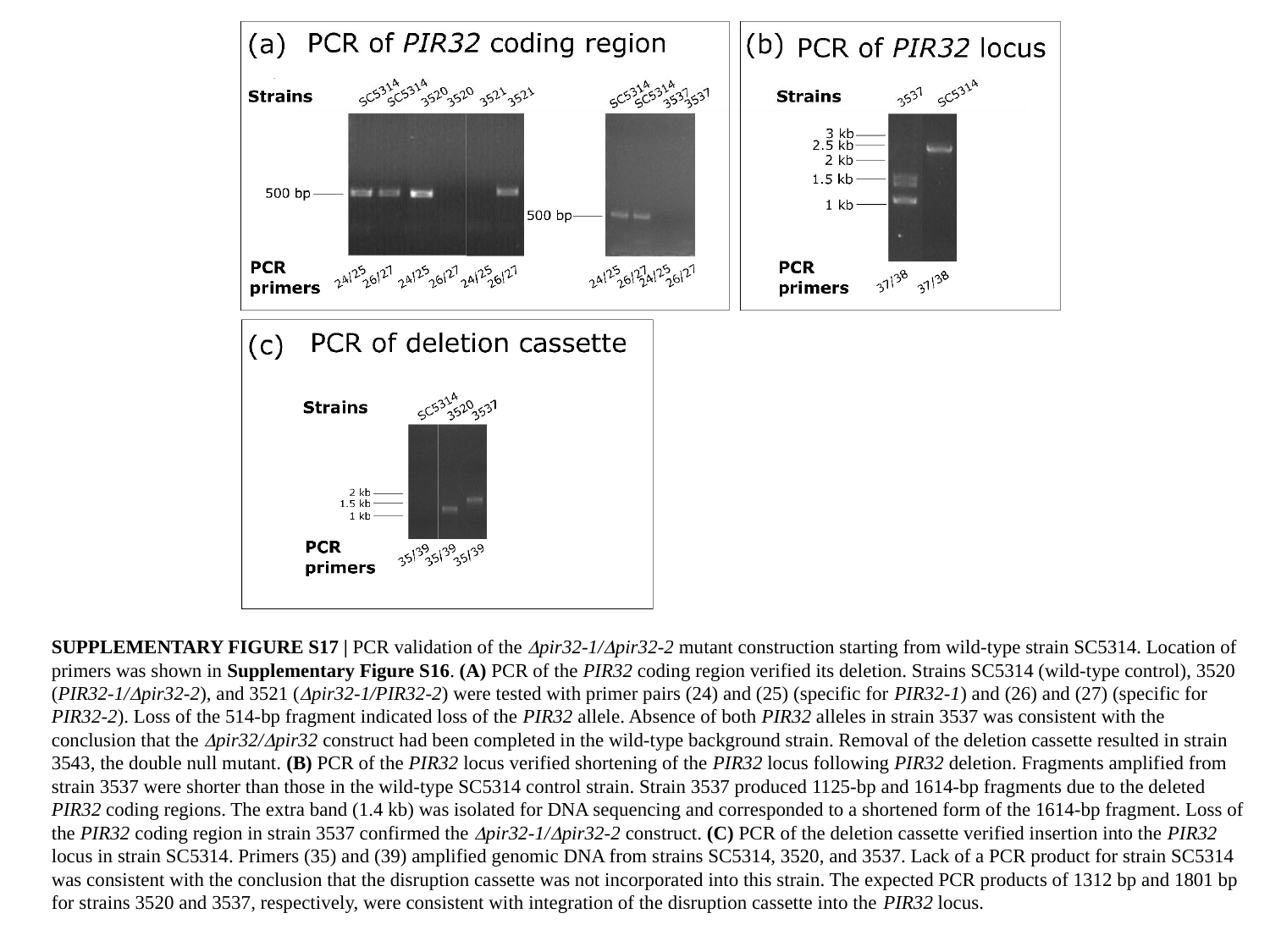

SUPPLEMENTARY FIGURE S17 | PCR validation of the Dpir32-1/Dpir32-2 mutant construction starting from wild-type strain SC5314. Location of primers was shown in Supplementary Figure S16. (A) PCR of the PIR32 coding region verified its deletion. Strains SC5314 (wild-type control), 3520 (PIR32-1/Dpir32-2), and 3521 (Dpir32-1/PIR32-2) were tested with primer pairs (24) and (25) (specific for PIR32-1) and (26) and (27) (specific for PIR32-2). Loss of the 514-bp fragment indicated loss of the PIR32 allele. Absence of both PIR32 alleles in strain 3537 was consistent with the conclusion that the Dpir32/Dpir32 construct had been completed in the wild-type background strain. Removal of the deletion cassette resulted in strain 3543, the double null mutant. (B) PCR of the PIR32 locus verified shortening of the PIR32 locus following PIR32 deletion. Fragments amplified from strain 3537 were shorter than those in the wild-type SC5314 control strain. Strain 3537 produced 1125-bp and 1614-bp fragments due to the deleted PIR32 coding regions. The extra band (1.4 kb) was isolated for DNA sequencing and corresponded to a shortened form of the 1614-bp fragment. Loss of the PIR32 coding region in strain 3537 confirmed the Dpir32-1/Dpir32-2 construct. (C) PCR of the deletion cassette verified insertion into the PIR32 locus in strain SC5314. Primers (35) and (39) amplified genomic DNA from strains SC5314, 3520, and 3537. Lack of a PCR product for strain SC5314 was consistent with the conclusion that the disruption cassette was not incorporated into this strain. The expected PCR products of 1312 bp and 1801 bp for strains 3520 and 3537, respectively, were consistent with integration of the disruption cassette into the PIR32 locus.

## Slide 20
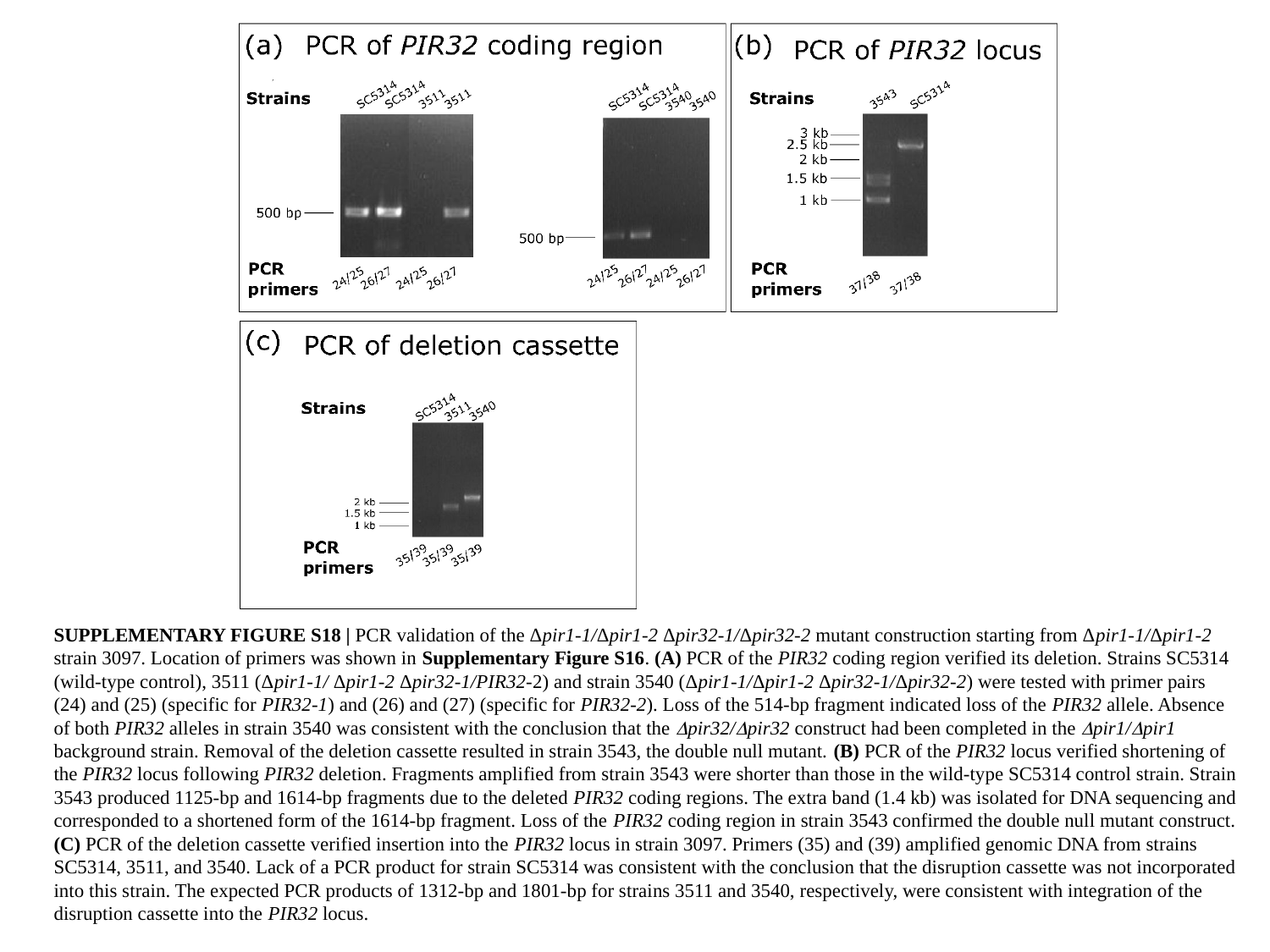

SUPPLEMENTARY FIGURE S18 | PCR validation of the Δpir1-1/Δpir1-2 Δpir32-1/Δpir32-2 mutant construction starting from Δpir1-1/Δpir1-2 strain 3097. Location of primers was shown in Supplementary Figure S16. (A) PCR of the PIR32 coding region verified its deletion. Strains SC5314 (wild-type control), 3511 (Δpir1-1/ Δpir1-2 Δpir32-1/PIR32-2) and strain 3540 (Δpir1-1/Δpir1-2 Δpir32-1/Δpir32-2) were tested with primer pairs (24) and (25) (specific for PIR32-1) and (26) and (27) (specific for PIR32-2). Loss of the 514-bp fragment indicated loss of the PIR32 allele. Absence of both PIR32 alleles in strain 3540 was consistent with the conclusion that the Dpir32/Dpir32 construct had been completed in the Dpir1/Dpir1 background strain. Removal of the deletion cassette resulted in strain 3543, the double null mutant. (B) PCR of the PIR32 locus verified shortening of the PIR32 locus following PIR32 deletion. Fragments amplified from strain 3543 were shorter than those in the wild-type SC5314 control strain. Strain 3543 produced 1125-bp and 1614-bp fragments due to the deleted PIR32 coding regions. The extra band (1.4 kb) was isolated for DNA sequencing and corresponded to a shortened form of the 1614-bp fragment. Loss of the PIR32 coding region in strain 3543 confirmed the double null mutant construct. (C) PCR of the deletion cassette verified insertion into the PIR32 locus in strain 3097. Primers (35) and (39) amplified genomic DNA from strains SC5314, 3511, and 3540. Lack of a PCR product for strain SC5314 was consistent with the conclusion that the disruption cassette was not incorporated into this strain. The expected PCR products of 1312-bp and 1801-bp for strains 3511 and 3540, respectively, were consistent with integration of the disruption cassette into the PIR32 locus.
