# Supplementary material for: Peering Into Candida albicans Pir Protein Function and Comparative Genomics of the Pir Family
Source: Front Cell Infect Microbiol. 2022 Mar 18;12:836632. doi: 10.3389/fcimb.2022.836632 (PMC8975586; doi:10.3389/fcimb.2022.836632)
Supplement: Supplementary file 2 [file Table_1.docx]

**SUPPLEMENTARY TABLE S1 |** Antifungal susceptibility readings for SC5314 and the Δ*pir/*Δ*pir C. albicans* strains.

Range Tested

MIC Value (μg/ml)

**Δ*pir1/*Δ*pir1***

| **Antifungal** | **(μg/ml)** | **SC5314** | **Δ*pir1/*Δ*pir1*** | **Δ*pir32/*Δ*pir32*** | **Δ*pir32/*Δ*pir32*** |
| --- | --- | --- | --- | --- | --- |
| Amphotericin B | 0.12-8 | 0.05 | 0.05 | 0.05 | 0.05 |
| Anidulafungin | 0.015-8 | 0.03 | 0.06 | 0.12 | 0.06 |
| Caspofungin | 0.008-8 | 0.06 | 0.06 | 0.06 | 0.06 |
| Fluconazole | 0.12-256 | 0.25 | 0.25 | 0.25 | 0.25 |
| 5-Flucytosine | 0.06-64 | ≤ 0.06 | ≤ 0.06 | ≤ 0.06 | ≤ 0.06 |
| Itraconazole | 0.015-16 | 0.06 | 0.03 | 0.03 | 0.03 |
| Micafungin | 0.008-8 | 0.015 | 0.015 | 0.015 | 0.015 |
| Posaconazole | 0.008-8 | 0.015 | 0.015 | 0.015 | 0.015 |
| Voriconazole | 0.008-8 | ≤ 0.008 | ≤ 0.008 | ≤ 0.008 | ≤ 0.008 |
