# Supplementary material for: Peering Into Candida albicans Pir Protein Function and Comparative Genomics of the Pir Family
Source: Front Cell Infect Microbiol. 2022 Mar 18;12:836632. doi: 10.3389/fcimb.2022.836632 (PMC8975586; doi:10.3389/fcimb.2022.836632)
Supplement: Supplementary file 3 [file Table_2.docx]

**SUPPLEMENTARY TABLE S2 |** *C. albicans* proteins revealed in BLAST searches using *S. cerevisiae* and *C. albicans* Pir proteins as the query and their sequence conservation.

**(A)** *C. albicans* proteins revealed in BLAST searches using *S. cerevisiae* and *C. albicans* Pir proteins as the query. Gene/protein descriptions were from the *Candida* Genome Database (CGD; [www.candidagenome.org](http://www.candidagenome.org)). *C. dubliniensis* loci used as orthologs for the RNA-Seq calculations (Palige et al., 2013) are indicated in parentheses. Sequence conservation among the predicted proteins is shown in **(B)** below.

**Ca*Δnrg1* CdWT CdWT**

**Locus Names Gene/Protein Description vs. CaWT**^†^ **vs. CaWT**^†^ **vs. Ca*Δnrg1***^†^

CR_04990C; orf19.654* *CIS305* Highly induced during chlamydospore development 9.5 17.9 8.4

(*Cd30570* and *Cd30400* were used as orthologs)

CR_05190W; orf19.555* *CIS304* Similar to *S. cerevisiae* Cis3 which functions in cell- 11.2 ---^§^ ---^§^

wall-related processes; predicted Kex2 substrate;

possibly an essential gene, disruptants not obtained

by *UAU1* method

CR_05430W; orf19.3512 *CSP1*, *CIS310* Putative cell wall-associated protein; highly upregulated 9.6 17.2 7.6

during chlamydospore development; localized to the

chlamydospore cell wall; (*Cd30750* ortholog)

C1_03890W; orf19.4463 *CIS308* (*Cd03620* ortholog is a pseudogene) 8.4 13.5 5.1

C1_11710C; orf19.1148 *CIS312* (*Cd11020* ortholog) -1.7 9.1 10.8

C2_00050C; orf19.1920 *CIS301* (*Cd15040* ortholog) -3.2 4.3 7.5

C2_04390W; orf19.4515 *CIS303* Similarity to cell wall proteins; possibly an essential gene, 0.7 10.5 9.8

disruptants not obtained by *UAU1* method; (*Cd19040* ortholog)

C2_06550W; orf19.31 *CIS302* (*Cd20900* ortholog) 0.7 8.4 7.7

C4_00720W; orf19.4170 *CSP2*, *CIS309* Putative cell wall associated protein; highly induced 8.5 12.3 3.8

during chlamydospore development; localized to the

chlamydospore cell wall; Hap43 repressed; Spider

biofilm induced; (*Cd40770* ortholog)

C2_08870C; orf19.220 *PIR1* 0.6 -4.2 -4.8

C1_07620C; orf19.2783 *PIR32* -1.4 -1.1 0.2

*The amino acid sequences for Cis304 and Cis305 are 99.6% identical.

^†^ Three different strains were grown under conditions that favor chlamydospore formation. WT = wild type. *C. albicans* forms chlamydospores more readily with a mutation in the *NRG1* gene (Staib and Morschhäuser, 2005). Strong up-regulation for each data comparison was interpreted to identify potential chlamydospore-specific genes.

^§^*C. dubliniensis* does not have a positional ortholog for *C. albicans* Cis304.

**(B)** Percent identity matrix for amino acid sequences as displayed in **Supplementary Table S3**, calculated using Clustal Omega.

|  | Cis301 | Cis302 | Cis303 | **Cis304** | **Cis305** | **Cis309** | **Cis310** | Cis312 | Cis308 | 15040 | 20900 | 19040 | 30570 | 40770 | 30750 | 11020 | 30400 |
| --- | --- | --- | --- | --- | --- | --- | --- | --- | --- | --- | --- | --- | --- | --- | --- | --- | --- |
| Cis301 | 100 |  |  |  |  |  |  |  |  |  |  |  |  |  |  |  |  |
| Cis302 | 39 | 100 |  |  |  |  |  |  |  |  |  |  |  |  |  |  |  |
| Cis303 | 36 | 85 | 100 |  |  |  |  |  |  |  |  |  |  |  |  |  |  |
| **Cis304** | 35 | 37 | 37 | 100 |  |  |  |  |  |  |  |  |  |  |  |  |  |
| **Cis305** | 35 | 37 | 37 | **100** | 100 |  |  |  |  |  |  |  |  |  |  |  |  |
| **Cis309** | 35 | 41 | 40 | 66 | 66 | 100 |  |  |  |  |  |  |  |  |  |  |  |
| **Cis310** | 35 | 38 | 36 | 61 | 61 | 80 | 100 |  |  |  |  |  |  |  |  |  |  |
| Cis312 | 31 | 39 | 39 | 46 | 46 | 43 | 40 | 100 |  |  |  |  |  |  |  |  |  |
| **Cis308** | 35 | 39 | 37 | 83 | 83 | 61 | 55 | 43 | 100 |  |  |  |  |  |  |  |  |
| 15040 | 73 | 42 | 38 | 38 | 38 | 39 | 38 | 32 | 38 | 100 |  |  |  |  |  |  |  |
| 20900 | 40 | 91 | 84 | 37 | 37 | 40 | 37 | 39 | 38 | 43 | 100 |  |  |  |  |  |  |
| 19040 | 39 | 85 | 88 | 39 | 39 | 39 | 37 | 38 | 37 | 41 | 89 | 100 |  |  |  |  |  |
| 30570 | 36 | 41 | 41 | 82 | 82 | 61 | 56 | 45 | 76 | 39 | 41 | 41 | 100 |  |  |  |  |
| 40770 | 34 | 42 | 40 | 66 | 66 | 93 | 80 | 43 | 59 | 39 | 41 | 41 | 63 | 100 |  |  |  |
| 30750 | 35 | 38 | 36 | 59 | 59 | 78 | 86 | 41 | 53 | 39 | 38 | 38 | 55 | 78 | 100 |  |  |
| 11020 | 31 | 39 | 40 | 44 | 44 | 43 | 41 | 84 | 42 | 33 | 39 | 39 | 44 | 43 | 41 | 100 |  |
| 30400 | 36 | 42 | 41 | 82 | 82 | 62 | 57 | 45 | 76 | 38 | 41 | 41 | 99 | 64 | 55 | 43 | 100 |
