# Supplementary material for: Peering Into Candida albicans Pir Protein Function and Comparative Genomics of the Pir Family
Source: Front Cell Infect Microbiol. 2022 Mar 18;12:836632. doi: 10.3389/fcimb.2022.836632 (PMC8975586; doi:10.3389/fcimb.2022.836632)
Supplement: Supplementary file 4 [file Table_3.docx]

**Supplementary Table S3** | *PIR* genes and predicted proteins from 16 fungal species.*

| **Gene** | **Sequence Identifier(s)** | **CGOB Ortholog**  **Group** **(OG)**^†^ | **Size (nt)** | **Size (aa)** | **Sig Pep (aa)** | **Kex2 Site (pred, not)** | **O-Glycos (#, %)** | **N-Glycos (pred, not)** | **Cys**  **(total, # of 4 cons)** | **Protein Sequence** |  |
| --- | --- | --- | --- | --- | --- | --- | --- | --- | --- | --- | --- |
| ***Candida albicans* SC5314** | | | | | | | | | | | |
| *CaPIR1* | orf19.220 | A | 1158 | 385 | 18 | 0, 0 | 62, 16% | 0, 1 | 4, 4 | MKYSTLVSIAAFISTSLAATVPDEHYSTLSPSAKIPSGASTDFSGTFGIQVVTVESASALSTDTATSTLTRNDNKKEATPVAQITDGQVQHQTTGGVSAIKQISDGQVQHQTNAAQPIAQISDGQIQHQTTAKATATPVQQINDGQIQHQTTVQPVAQISDGQIQHQTAKATATPVQQIGDGQIQHQTTVQPVAQISDGQIQHQTVKASATPVQQIGDGQIQHQTTAAAATTASAVKQINDGQIQHQTTTAENVAKAQSDGQAIATGSPSSNSTLSDDDDLSSTIPKACSSANNLEMTLHDSVLKDTHERWGAIVANHQFQFDGPIPQAGTIYSAGWSIKDGYLYLGDSNIFYQCLSGDFYNLYDENVAKQCSAVKLSVIEFVNC |  |
| *CaPIR32* | orf19.2783 | B | 1269 | 422 | 21 | 0, 2 | 14, 3% | 1, 3 | 7, 4 | MIHYLIFPILLIFFQIIKSSGYYVPANGDDWTILKPPSCKNKLPGQYIDSLPFTFGIVVNPYILNEDGDYEKPIVSKIKPSLTTTTFVTSIITTSTATAPGSKPTKTKDIIVQIHDGQVQKMKHKHDYSSGGGDDDDDDEDCFDEKNIDMAAKRDYQDMNTQEQANEDSGQVLAESDSHQQVVDQNQQINEEEEETQEQQMQEENNNTNEIEDNKVQEFETIEEIYDDIDNNESRPNNSKKYHKKRPHNNYENKHGHKDYHEDHHHNHRYKDHENGHEEDDHKWNKPKPMPEQEEQEQEQEQKPKHEKSEGYESEFVSPVYSVACYTNSTLKMTLTDGILRDSDDRIGCIVSGHQFQFDGPTPQHGAIYAAGWSVTKQGQLALGDSTKFYQCASGDFYNLYDEPIAFQCHPVTLDVVELIEC |  |
| *CaCIS301* | orf19.1920 | C | 534 | 177 | 18 | 0, 1 | 0, 0% | 2, 0 | 7, 3 | MICLVLFYTIVLVSGLASAPIRFSLGAQVGYTTYKVFEIGDGQLFHGNATVECLMGNSSYMAPEPCGKCKVTPVYCKVGKSPQYFMLSDGVLYDQRGAIGEIVANHQFQFDLPSQSSALFNKGWSVVSSNGKRLLSLHGSTRFWHCQAGTNGLYKLYDASIGGQCSPVYIVVLQQVW |  |
| *CaCIS302* | orf19.31 | D | 756 | 251 | 18 | 0, 1 | 2, 0.7% | 0, 0 | 16, 3 | MKFSTVALSLPLIAMVQAGISSPTYYKPEHTEEIGVETVDYEFALGVREKCDEDTIYLAYEVDDGQLERNDKKVDCNCKSEPVVYPTPRPSKTYWDGGDDNDECDEDCDDEDKKKGHKQYKRGEVEEPCETSDCDFCTIEKSFCQDQFTLCEGVLKDQRSAIGSIVANHQFQFDNPIQKDALHTCGWSIVEKDCVKLLALDGCTDFWECPVDDCDTYKLYDSSIDDKCKEIEIIVILFEEEEEEKKKHKSW |  |
| *CaCIS303* | orf19.4515 | E | 744 | 247 | 18 | 2, 0 | 5, 2% | 0, 0 | 15, 3 | MKFSTVALSLPLIAMVQAGISSPTYYKPEHNEEHGVETVEFEFALGVREKCDDSIIYLVYEVDDGQLERNDKKLDCNCKSERVSRPAPSPSAIAVVGGNEECDEDCDDEHRKKGSKQYKRGEVENPRETRDCDFCTIEKSFCQDHFTLCEGVLKDQHSAIGSIVSNHQFQFDKPAQKDALHTCGWSIVEKDCVKLLAVDGCTDFWECPVDDCDTYKLYNSAIDAKCKEIEIIVILLEEEEERKHKSW |  |
| *CaCIS304* | orf19.555 | F | 828 | 275 | 18 | (1), 2 | 1, 0.4% | 0, 0 | 15, 3 | MRFSIATLSLAALTVVSASYVTRGEGVSRGEKYECDFDTFEWKFGLAVKELKHRGKNWGKDVDLDIVYESDDGQLYHGCKDTYDASKCKNCYETFEFSDDDDEGSDCDDDDCKKKKKAHRYTKRCGGGDDDDCEDDERCNYPYCELYDDNCDLVITLRDGVLHDERHATGEIVANHQFQFDKPPQKDALHKKGFSIVYTEGNYYLALDHKIKFWHCKVDDNGLYKIYDKSIGEQCSEIELIILKSDKKAEFEFSDNEGSDCDDDCKRKKKHGKKY |  |
| *CaCIS305* | orf19.654 | G | 831 | 276 | 18 | (1), 2 | 1, 0.4% | 0, 0 | 15, 3 | MRFSIATLSLAALTVVSASYVTRGEGVSRGEKYECDFDTFEWKFGLAVKELKHRGKNWGKDVDLDIVYESDDGQLYHGCKDTYDASKCKNCYETFEFSDDDDDEGSDCDDDDCKKKKKAHRYAKRCGGGDDDDCEDDERCNYPYCELYDDNCDLVITLRDGVLHDERHATGEIVANHQFQFDKPPQKDALHKKGFSIVYTEGNYYLALDHKIKFWHCKVDDNGLYKIYDKSIGEQCSEIELIILKSDKKAEFEFSDNEGSDCDDDCKRKKKHGKKY |  |
| *CaCIS309* | orf19.4170, *CSP2* | H | 825 | 274 | 18 | 0, 1 | 0, 0% | (1), 0 | 17, 3 | MKFSTAALTLSLFAIVNSSVTTYGAGFGGDDDDNSKHHKYDCEIDTYEWKFALAVKEWKHCQDKKFCQDTDLDLVYEGDDGQLYHGCDGTYPYSKCKHCTNVFTGGDDDDDNCDDDCKKKKKKVYFAKRGDDDDDDKCDDKCEYPYCEIHNTDCDLLITLCGGVLTDDKHATGEIVANHQFQFDKPPQKDALHKKGFSIVHTEGTYYLALDHKIDFWHCKVDDKGLYKIYDKSIGEQCSKIKLIVLKSDEKATFTDDSDDCDDDCKKNKKKQHD |  |
| *CaCIS310* | orf19.3512, *CSP1* | I | 1065 | 354 | 18 | 0, 1 | 1, 0.3% | (1), 0 | 31, 3 | MKFSTAALTFSLLAIVNSSVITYDTEFGDNYEHKHDKYDCEIDTFNWKFALVVKEWKYHKDEKFCKDTDLDLVYEGEDGQLYHGCDGTYPYSKCKHCTNVFAGGDDNKWKDDKWDDKYKDKKCHDKWDDKCKDKCDDKHKDDHCKDKKCDDKWDNKCHDKCKDDDKCHDKCKDKKCDDKQKHDHCKDDNKCHDKCKDKKCDDKCKDGDDKCHDDCKDDDKCDKHHCKYPYCEIHNTDCDLLITLCNGVLKDKRDATGEIAANHQFQFDKPPQKDALHKEGFSIVHTEGTYYLALNHKIEFWHCKVDDKGLYKIYDKSIGPQCSKIELIVLKSDEKATFSNDDCDDDCKKKQKHD |  |
| *CaCIS312* | orf19.1148 | J | 792 | 263 | 18 | 0, 2 | 1, 0.4% | 1, 0 | 1, 0 | MKFSLATATLGLIAIAQATLTPYSGYSGYSGGDDDKYRHGKSGIETLRGKFALAVKELSGYEHGKHKDRDLHIVYEIDDGQLQYGDRKDYIYYPYRNENSEEECSDEDDDHHKKKKRPHRHGGKSDDDDDDKKWKRGGDYSDDNDNDSDDDLRFLTISYNDKYDFFTLKNTVLHDEKGATGEIVANHQFQFDKPPQKDALYDKGFTIVEKDDYYVLALKGKTKFWNSAVDDKGAFKIYDEKINENSKPIELIILKVEKHGKKY |  |
| *CaCIS308* | orf19.4463 | None | 900 | 299 | 18 | (1), 2 | 2, 0.7% | 1, 0 | 17, 3 | ~~MLSLTKTPTETATTKTHTETKTINTAQISLQIFSFFFFSFHHYLIKYQINQLTNSII~~  MRFSIATLSLAAFTVVNAGYISRGEGLTRGEKYERHECDFDSFEWKFGLAVKELKHHGDKNWGRDVELDLVYESDDGQLYHGCKDVYKASKCRNCYETFEFSDNESSDDEGSGSDCEDDECKKRKKKVHRNYKRGGYGSERRQSDCESDCERSERCNYPFCELYEDNCDLLLTLCDGVLHDDRHATGEIVANHQFQFDKPPQKDALHKKGFSIVYTHGNYYLALDNKIKFWHCKVDDNGLYKIYDKSIGEQCCEIELIILKSDKKAEFEFTDRESDSEGSDCDDDCKKNKKHGKKHSGY |  |
| ***Candida dubliniensis* CD36** | | | | | | | | | | | |
| *Cd23050* | CD36_23050, XM_002418734 | A | 879 | 292 | 18 | 0, 0 | 0, 0% | 1, 0 | 4, 4 | MKYSTLVSIAAFISSSLAATVPDEHYSTLSPSGKIPTGASTDFSGTFGIQVVTVESASALSTDTATSTLTRNDNKDVKATPIAQITDGQIQHQTNAQPIAQISDGQIQHQTVKATPVQQINDGQIQHQTTTAPTTTAASAVKQINDGQIQHQTTSTVDNVAKAQSDGQAIATGSNSNSTANIDDNENSLSSILKACSSKNNLQMTLHNSVLKDTNERWGSIVANHQFQFDGPIPQAGVIYSSGWSIKDGYLYLGDSNIFYQCLSGDFYNLYDENVAKQCSAVKLSVIEFVNC |  |
| *Cd07100* | CD36_07100, XM_002417322 | B | 1254 | 417 | 20 | 0, 1 | 0, 0% | 1, 3 | 7, 4 | MINYLIISILLISHIIKSSGYYVPANGDDWTILIPPSCKNKSPDQYFDSLPFAFGIVVNPYILNEDGDYEKPIVSKIKPSLTTTTFVTSIITTSTATAPNSKPTKTKDIIVQIHDGQVQKMKPKHDYSSEDEEDCFDKKNINIAAKRDYQDMNPQQQETNDDSGQVVVAESDSQLPGLENDQNQLINEEAEAEVESTPQEQQKPEENNSQEFETIEEIYYDDIDNDNESRPNNGKKYHKKKPHNYENKHGHKDYHHEDHHHNHRYKDHEHDHEHDNEHDNNKGDHKWNKPKQTPEKEESEQEQEEEEETEFISPVYSVACYTNSTLKMTLNDGILRDSDDRIGCIVSGHQFQFDGPTPQHGAIYAAGWSVTKQGQLALGDSTKFYQCASGDFYNLYDEPIAFQCHPVTLDVVELIEC |  |
| *Cd15040* | CD36_15040, XM_002417938 | C | 549 | 182 | 15 | 1, 0 | 5, 3% | 0, 0 | 7, 3 | ~~MYPCNFYFFFFFCPA~~  MIFLVLVCLFVLVRGGLVKNEVDSVGYRFSLGVETGNSIYKVFEIGDGQLFHGNTTVQCLTGNSTYQTPDPCGNCESAAYCKVRKSPQYIVLTSGVLRNQRGAIGEIVANHQFQFDLPSQPDALFNKGWSIVSWNGKSLLALNGDTQFWHCQAGTNGLYKLYNASIGGQCSPVYIVVLQQVW |  |
| *Cd20900* | CD36_20900, XM_002418523 | D | 750 | 249 | 18 | 0, 1 | 0, 0% | 0, 0 | 16, 3 | MKFSTIALSLPLIAMVQAGISSPTYYKPEHTEVTGVETVDYEFALGVREKCDEDIIYLVYEVDDGQLERNDKKVDCNCKSEPVIYPTPRPSKTYWSGGDDGECDEDCDDEHKKKGYKRGEVEEPCETPTCDYCTIEKSFCQDHFTLCDGVLKDQRSAIGSIVSNHQFQFDNPVQKDALYTCGWSIVEKDCVKLLALDGCTDFWECPVDDCGTYKLYNSAIDSKCKEIEIIVILFEEEEEEEKKKKHTSW |  |
| *Cd19040* | CD36_19040, XM_002418339 | E | 741 | 246 | 18 | 0, 1 | 0, 0% | 0, 0 | 16, 3 | MKFSTVALSLPLIAMVQAGISSPTYYNSEHNEEHGLETVDYEFALGVREKCDEDTIYLVYEVDDGQLERNEKKVDCNCKSERVFRPTPSPSAIAVVGGNEECDENCDDEHKKKIYKRGEVEEPCETTNCDYCTIEKSFCQDHFTLCDGVLKDQRSAIGSIVSNHQFQFDKPAQKDALYTCGWSIIEKDCVKLLALDGCTDFWECPVDDCGTYKLYNSAIDSKCKEIEIIVILFEEEEEENKKHTSW |  |
| *Cd30570* | CD36_30570, XM_002422024 | G | 822 | 273 | 18 | 0, 2 | 0, 0% | 1, 0 | 15, 3 | MKFSIATLSLATLAVVSAGYVSRGEGVSRGEKFECDFDTFEWKFALAVKELKHKGKNWGRDVDLDIVYELDDGQLFHGCRETYDASKCKNCYEAFEFNEEDNDKDCDDDCKRKKKGHRNYKRGGYSDGDCDDDCERVERCNYPFCELYDDNCDLLITLRDGVLRDEKHAIGEIVANHQFQFDKPPQKDALHKKGFSIVYTEGNYYLALDHKIKFWHCKVDDKGLYKLYNESIGEQCSEIELIILKSNERAEFNEREEECDDDCKRKKEHGKKY |  |
| *Cd40770* | CD36_40770, XM_002419704, *CSP2* | H | 822 | 273 | 18 | (1), 1 | 0, 0% | (1), 0 | 17, 3 | MKFSTAALTLSLFAIVNSSVTTYGAGFSGDDDDNSKHHKYDCEIDTFDWKFALAVKEWKHCQDKKFCQDTDLDLVYEGDDGQLYHGCDGTYPYSKCKHCTNVFTGDDDDDANCDDDCKKKKKKVYYAKRGDDDDDDNCDDDCKYPYCEIHNTDCDLLITLCGGVLKDEKHATGEIVANHQFQFDKPPQKDALHKEGFSIVHTEGTYYLALDHKIDFWHCKVDDKGLYKIYDKSIGEQCSKIKLIVLKSDERATFSDDNDDCDDDCKNKKKQHD |  |
| *Cd30750* | CD36_30750, XM_002422036, *CSP1* | I | 1080 | 359 | 18 | (1), 0 | 0, 0% | 1, 0 | 27, 3 | MKFSTAALTFSFFAIVNSSVVTYSTEFGGNEEHKHDKYDCEIDTFDWKFALVVKEWKHHKDEKFCKDTDLDLVYEGDDGQLYHGCDGTYPYSKCKHCTNVFGGGDDDNKWDNKWKDDKGDDKWKDKKCDDKWDDKHKDDHCKDDNGKDGKCKDDDKCKDKKCDDKWDGNCKDDHGKDDKCKDDGKWDDKCKDKCDDKHKDDNCKDEKCDDKCKDDEKCHDHWKGDDKCDKHHCKYPYCEIHNTDCDLLITLCNGVLKDKRDATGEIAVNHQFQFDKPPQNDALHKKGFSIVHTEGTYYLALNHQIEFWHCKVDDKGLYKIYDKSIGDQCSKIELIVLKSDEKATNDDCDDDCKKKEKYD |  |
| *Cd11020* | CD36_11020, XM_002417698 | J | 774 | 257 | 18 | (1), 2 | 0, 0% | 0, 0 | 1, 0 | MKFSFATATLGLIAIAQATLTSYSGYSGYSGDDDDKFKHERNGIETLRGKFALAVKELSGYEHGKNKDRGLHIVYEIDDGQLQYGDRKDYIYYPFRNENNEEECEDEDDDDHKKKKRPHKYGGKSDDDDNKNWKRGDSDSDDNNDIRFLTIDYNDKYDFFTLTNTVLHDKRGATGEIAANHQFQFDKPPQKDALFDKGFTVVVKDGYYVLALKGNTKFWNSAVDDKGAFKIYNEKINEKSEPIELIILKVEKDDKKY |  |
| *Cd30400* | CD36_30400, XM_002422009 | None | 978 | 325 | 18 | 0, 2 | 0, 0% | 0, 0 | 15, 3 | ~~MFLLLEPNNNIYNTYCNENYKYSPKVLPNIHFFFLLFITTYSSIKPINQSII~~  MKFSIAALSLATLAVVSAGYVSRGEGVSRGEKFECDFDTFEWKFALAVKELKHKGKNWGRDVDLDIVYELDDGQLFHGCRETYDASKCKNCYEAFEFNEEDNDKDCDDDCKRKKKGHRNYKRGGYSDGDCDDDCERVERCNYPFCELYDDNCDLLITLRDGVLRDEKHAIGEIVANHQFQFDKPPQKDALHKEGFSIVYTEGNYYLALDHKIKFWHCKVDDKGLYKLYDESIGEQCSEIELIILKSNERAEFNEREEECDDDCKRKKEHGKKY |  |
| ***Candida tropicalis* MYA-3404** | | | | | | | | | | | |
| *Ctr01952* | CTRG_01952, XM_002547599 | A | 1149 | 382 | 19 | 1, 0 | 66, 17% | 0, 0 | 5, 4 | MKYSTLFTIAAFLCSSSLAATLPEENYSTLSPSAHALSGATTDFTGTFGIQIVTVESASALSTDKSTSTLTKDKRAVATPVAQISDGQIQHQTTAAPVQQISDGQIQHQTKATTTASPVKQISDGQIQHQTKATTTASPVKQISDGQIQHQTKVTTTASPVKQISDGQVQHQTKATTTASPVKQISDGQIQHQTTAASAVKQISDGQIQHQTTASAIKQISDGQIQHQTTATAAAQISDGQVQHQTTATGAAQITDGQVNANSDSSASTTAKDDSEDDDDSLPQACSAENNLEMTLHDGVLKDSLNRWGSIVANRQFQFDGPIPQAGVIYSAGWSIRNGYLYLGDSDVFYQCLSGDFYNLYDENVAAQCSAVKLSVIEFVDC |  |
| *Ctr04172* | CTRG_04172, XM_002549829 | B | 1317 | 438 | 19 | 1, 1 | 12, 3% | 0, 0 | 6, 4 | MLQLIFILSIFNNIFPIDAYYVPANGEDWTILKPDCQKLQGSFESLPFTFGIVVNPYVINDEGDYEEPVVSKIERTITTSFVTSVVTAAPKPTKTRDIIVQIHDGQVQKVASGYDWKNEEKHKDWDDDHHYDWKDDDKHYTDYDKSHDDDNDKHHQDKDYDDYDKHHDDKHHKDKQYDDDKDDYYKHRKSDVDDVLMDPTTKRRKRNNQETGEQVEKLADDEAEGQGVETVEEVYEDIGKDHGYEKDQGYNQDLDFGKGRDYDREHDHDKDHDHDKHHDYYDDGDDKHHKNKDDHENKHGHKDYNEEHKHRYKDDWKHEEPSRDYDYDDDQFVSPVYYVACYTNATLRMTLNNGILRDSDNRIGCIVSGHQFQFDGPTPQHGAIFAAGWSVTKEGQLALGDSTKFYQCASGNFYNLYDEPIAFQCHPVTLDVVELIEC |  |
| *Ctr04756* | CTRG_04756, XM_002550412 | D | 684 | 227 | 18 | 0, 1 | 3, 1% | 0, 0 | 14, 3 | MKFSTAALTFTLFAIVNAGVAKPKYYKPKTKLVGLETVDYEFALGVKEKCDGDTIYLAFEVGDGQLEHNGQVVDCKCEKDKRGYSPPAPVEYCTIAKEDCVDTFTLCESILKDECERIGEIVANHQFQFDNPIQPDALFTCGWSIIEDDGYLLLGLNGCTDFWECPVDDCGTYKLYDASIDSKCKEVEIIVLLIEEEEECPPEPCEPTPEPFTPPYKGHKGHKGGKW |  |
| *Ctr01592* | CTRG_01592, XM_002547240 | E | 732 | 243 | 18 | 0, 1 | 0, 0% | 0, 0 | 16, 4 | MKFSAVTLSLSLFAMVSAGVAKPKYYKPKPKPKLVGLETVDYEFALGVKEKCDGDVIYLAFELEDGQLEHNGEVVDCKCEKDKRGYIPPTPVDYCTIDKTDCVDTFTLCETILKDECDSIGEIVANHQFQFDNPVQPDALFTCGWTIVKEDGYLLLALNGCTDFWECPVDDCGTYKLYDASIDSKCKEVEIVVILIDEECPPEPVYEPECPPEPCEEECPPEPVYVPEPKKGKKGKGKKGGKW |  |
| *Ctr01767* | CTRG_01767, XM_002547414 | H | 747 | 248 | 22 | 1, 0 | 3, 1% | 0, 0 | 16, 4 | MNMRFVATATILTFNLFRSIQAGIVPIEDCNKCEVDTFDGEFVLAIKELGFDYKNCKFLDLIYEIDDGQLEHGGRQDQLYPISLCKYCSLVDNCGKDCGEDGVVKLKETPKISKRGGATVPEPGCDEPYCKICKDDCIWSFSLCDGILTDKNYATGEIVANHQLQFDKSSQIDAFHTCGFSITYQYGNYLLALNHKTLFWHCKVDDYGLYKIYDKKIGAQCSPVQLIVLQAKQCKKKYDHKSFPASSS |  |
| *Ctr03251* | CTRG_03251, XM_002548908 | L | 936 | 311 | 16 | 0, 3 | 29, 9% | 0, 0 | 3, 0 | MIFTILILIASRIIQASYVPRDGYGGGDDYDNKHEGKCDKCDKKGDDGKDDGYNYVDSYDGKFYLGVKEYSNDKYYDIIYQLNDGQIEYGNYDKYPIKRETNSDCDTTTITTTYYNTTTYTTVIVSQTDESTITDSSDFVITEEPTDEPFFEEPSDEPFDGTPSDDFPSEVIAEEPSTSIFEELPESSSSSLLSSSSEGVYKRNESTDHHKPSTYYWFTLHKNVLKDSKKRIGEIVANHQFQFDYPVQPDALYTKGFSIVTYKGVKYLALNGNTKFWNSAVNDKGVYKIYDKPITDQSKPINLVILEPSKK |  |
| **Lodderomyces elongisporus NRRL YB-4239** | | | | | | | | | | | |
| *Le02549* | LELG_02549, XM_001525941 | A | 975 | 324 | 19 | 0, 1 | 41, 13% | 0, 0 | 3, 3 | MRITNTFATTAALISSTLAATVPSAPWSTLTPTGAIPTDASTDWSHSFGIQIETIEVASQLATETGTIAGKKRDVVNGLSDGQPNVIPTASYSILSESVAPVAQITDGQIQHQTTAAAASVVNQITDGQIQHQTTAAAASVVNQMLFQQPHIVYFPNSLHSLLKSQMVRSNTKPSSSSQVLLTKSQMVRSNTKPSSSSQVSLTKLVTDKFNTKLSSSSQVSLTKLVTDKFNTKPSLKSLSVLADSKGRVGAIVANRQFQFDGPPPQAGTIYAAGWSISSDGYLTLGDADVFYQCLSGDFYNLYDENVASQCNAVKLKIINFVDC |  |
| *Le00824* | LELG_00824, XM_001528254 | B | 1017 | 338 | 19 | 0, 0 | 4, 1% | 0, 0 | 11, 4 | MFHLSVSLPFVFLLYKTQAYYIPASGGDWTKLKPCSGNLPGSYVNLPFEFGIIVNQYHVENSGNDFLLDGLEIGSYNENLETTTIMTNTVISTPKAKLTRNIAFQINDGQVLNKAPGQECSKHSLNHRFSCDAGDTGVINDNRHNEHITSNNKDILLKLPSTNAVNEAQEPLCNKEVQSDNKIKKTNNKERKQGNGLLMQYGLYKKVYKKEREDGEETPEACKKGHSKFITPTNLVACYTDSSLRMKLEDSILRDSKGRIGCIVSNHQFQFDGPVPQHGAIYAAGWSVTMDGKLALGDCTKFYQCATGEFYKLYNQQIGPQCQPVTLETVELINCGGE |  |
| ***Candida parapsilosis* CDC 317** | | | | | | | | | | | |
| *Cp806490* | CPAR2_806490, XM_036808547 | A | 1203 | 400 | 18 | 2, 0 | 73, 18% | 0, 0 | 4, 4 | MKYTTIATSAAFLTTALAATVPSAPWTTLTPSASIPTDATTDYTSSFGIQIETVEAASALSTDSATDLSSKLETASATLGKRAVVSALSDGQPNVRSGSYSILPSSSSSASKSSVAPVAQITDGQIQHQTTAAAASVVNQITDGQIQHQTTQASVVNQIGDGQIQHQTTQASVVNQIGDGQIQHQTTQASVVNQIGDGQIQHQTSAASVINQIGDGQIQHQTTAAAASAVNQITDGQIQHQTTAEPTASAAAQISDGQIQHKNSTVAAESRAAATTLSDGQPQESGVSSSNDEDSSSSIPKACSSSNNLVMKLEDGVLTDSKGRIGAIVANRQFQFDGPPPQAGSIFAAGWSISKDGYLTLGDSKVFYQCLSGDFYNLYDENVAAQCNAVKLKIIDFVDC |  |
| *Cp208770* | CPAR2_208770, XM_036809799 | B | 1293 | 430 | 25 | (2), 0 | 6, 0.1% | 0, 0 | 7, 4 | MLSPRLLASFATINLIQAYYIPASGEDWTKYKPSCDYLPGSFTTLPFKFGIVVNPYIVTNEGELLEPEVESIERSITTSFVTSVVTPAPKATKTKDIIVQITDGQVQKVNEELCDPEEKKKLGLDEEHYHQHHDHDGYLSDKYGAKFDDKYGDKYGGKHKDGHYNENHRDGEFYDKDEKFGDHKHFDDDDKYNKEKQFNDKDFDKETVLSKRDELEIEDAHDIGGDIKDKPFDEHSKHYKDKHGLKGDDYDYENKHHLEKGEKDHNEYYNEHERSKQYESHEGSYTEHKDFKERYPHNDHKGHNHKDHNDHNGGAHDFFHDDDFASPVYSVACYTNATLRMSLHDSILRDSDDRIGCIVSGHQFQFDGPTPQHGAVFAAGWSVTKDGQLALGNSTKFYQCASGHFYNLYDQSIGFQCHPVTLDVVELIDC |  |
| *Cp201910* | CPAR2_201910, XM_036809042 | D | 804 | 267 | 19 | 0, 1 | 19, 7% | 0, 0 | 19, 4 | MQYSTILSIALALVSTAQAGYVKPRPITEPDCEPTTTTTPCEPTPTPCCQDSDITSFSGEFALGVKELCDEDDVIYLVFELPDGQLEHNGVTVDCGCHPTTTTTTPCEPTTTPCAEPEPIVDDCNDDKKKRWYKPKPKPVPTPIPTPNGLSALCHPYCSIEELVADCTTDYFTLCDSVLKDGKYRTGEIVANHQFQFDSPVQPDALYNKGWSIEYKDDYYLLALNDCTTFWECPVDDCGLWKLYDASIDSKCKEIEIVIIFKDESCD |  |
| *Cp205800* | CPAR2_205800, XM_036809474 | K | 855 | 284 | 18 | (2), 0 | 8, 3% | 0, 0 | 0, 0 | ~~MEPSQMPHSRGINNSSVSPYCTTLMLSKHKQQQFAINN~~  MKFSSVLATFGLLAITQSSLIKRGGDDDDNEGHNPQQVPITPVVPHRSKPHYTEKFTLGVKIDGQVWLTFQEEDGQLEFDHIKYYQHGSKPHWGGHHQTFTIQPVTEDDSEPTGHADWKRGYDPAEEEEEDDNYTAAKYYPKSYSIKYQPWLPIFTLKNTVLKDTHERTGSIVANHQLQFDNPVQPDALYTNGFSIKYVNGYPLLALNDKTTFWDSQASSPIWKLYDKPITYKSRSVELVVIKVVY |  |
| *Cp205520* | CPAR2_205520, XM_036809443 | L | 729 | 242 | 19 | 0, 1 | 8, 3% | 0, 0 | 3, 0 | MKFTSTLIAAFGFTAVANSNLIQRGYDHDDQPTPTYHPPYQPGVTSFPNKFALGAKIGDDIYVAFELDDGQLEYGPKKYTVPCVTPTPIVEPCDTPTPTPTPDEPCEEPTPEPITYPHHPHPHKPWKRDGFDFDNDDSWLTFDSSYGYDWYTLKNSVLRDSKYRIGEIAANHQLQFDLPVQPDALFSSGFSIVHDDGYWLLALNGKTTFWDSPVNDNGIYKIYDAPINPKSKKIELVVIVLV |  |
| *Cp201960* | CPAR2_201960, XM_036809048 | None | 804 | 267 | 19 | 0, 1 | 20, 7% | 0, 0 | 19, 4 | MQYSTILSIALALVSTAQAGYVKPRPITEPDCEPTTTTTPCEPTPTPCCQDSDITSFSGEFALGVKELCDEDDVIYLVFELPDGQLEHNGVTVDCGCHPTTTTTTPCEPTTTPCAEPEPIVDDCNDDKKKRWYKPKPKPVPTPIPTPNGLSALCHPYCSIEELVSDCTTDYFTLCDSVLKDGKYRTGEIVANHQFQFDSPVQPDALYNKGWSIEYKDDYYLLALNDCTTFWECPVDDCGLWKLYDASIDSKCKEIEIVIIFKDESCD |  |
| ***Candida orthopsilosis* Co 90-125** | | | | | | | | | | | |
| *Co0C02660* | CORT_0C02660, XM_003868497 | A | 1122 | 373 | 18 | 1, 0 | 64, 17% | 0, 0 | 4, 4 | MKYTTIATSAAFLTTVLAATVPSAPWTTLTPTASIPSDATTDYASSFGIQIETVEDASALSTDTAADLSSKLETASATLKRRAVVSALSDGQPNVRSGSYSILPTSSSSVAPVAQITDGQIQHQTTAAAASVVNQITDGQIQHQTTASVVNQITDGQIQHQTSAASVVNQIGDGQIQHQTSAASVVNQIGDGQIQHQTSAASVVNQITDGQIQHQTSAEPTATAAAQISDGQVQHKNSTVAAESRAAATTLSDGQPQESGASSDDDDSNSSIPQACSSSNNLVMKLEDSVLTDSHGRIGAIVANRQFQFDGPPPQAGSIFAAGWSISSDGYLTLGDSKVFYQCLSGDFYNLYDENVAAQCNAVKLKIIDFVDC |  |
| *Co0A08050* | CORT_0A08050, XM_003866581 | B | 1200 | 399 | 25 | 1, 0 | 5, 1% | 0, 0 | 7, 4 | MLSPRLLASFATINLIQAYYIPANGEDWTKYKPTCDYLPGSFTSLPFKFGIVVNPYTVTNEGELLEPEVKSIERSITTSFVTSVITPAPKETKTKDIIVQITDGQVQKVNEELCDPEEKKKLGLDNDHHDGYFSDKDGDKHGNGYYNADKHSNDDYREKDEKFGGRKHFDDDKLNKDKHFDDGDFETVLSKRDELDDEAAHDIGGDIKDKPFDEHFKHYKGKHGLKGDDYDLDNNHWEKDSGHYNEHERSKEHESHEGSYAEHRDFKERGRHNHKDHKGHKEHGDDFFNDNDFTSPVYSVACYTNATLRMSLHDSILRDSDDRIGCIVSGHQFQFDGPTPQHGAVFAAGWSVTKDGQLALGNSTKFYQCASGHFYNLYDQSIGFQCHPVTLDVVELIDC |  |
| *Co0D05920* | CORT_0D05920, XM_003869516 | K | 822 | 273 | 18 | 1, 1 | 7, 3% | 0, 0 | 0, 0 | ~~MLNGAQRCINSGSFSLNSIIYFLSSYKQQLNLINI~~  MKFSSILATFGLLAMSQSSLVKRGGDDDDYHPQPAPITPIVPYHSNPHYPEKFTLGVKVDGQIWLTFQEADGQLESDHIKYNPPSKPHWGDHRQTFTIQPVTEDGSEPTDHPGWKRGYDAGDDGDDTGASHYPRPLTIRYQSWLPVFTLKNTVLKDSNGRIGSIVANHQFQFDNPVQPDSLYTSGFSIEYEKGYPLLALNGKTTFWDSQASSSVWKLYDKPITYKSRSVELVVIRVIY |  |
| *Co0D05640* | CORT_0D05640, XM_003869489 | L | 726 | 241 | 19 | 0, 1 | 13, 5% | 0, 0 | 3, 0 | MKFTTALIAAFGFSAVANSSLIQRGYDDDDSYPTPTYNPPQQPGVTSFPNRFALGAKIDGDVYVVFELGDGQVEYGPKKYTVPCVTPTPIVEPCDTPSSTPTIEPCEEPTPEPITYTHPHPHKPWKRDGFDFDGDDSWLTFDSSYGYDWYTLRNSVLRDSKYRTGEIVANHQFQFDSPVQPDALYSSGFSIVHDDGSWLLALNGKTTFWDSPVNDNGIYKIYNAPINPKSRRIELVVIVLV |  |
| ***Candida metapsilosis* ATCC 96143** | | | | | | | | | | | |
| *CmPIR01* | MT017922 | A | 1122 | 373 | 18 | 1, 0 | 54, 14% | 0, 0 | 4, 4 | MKYSTIATSAAFLTTALAATVPSAPWTTLTPTASIPKDATTDYSSSFGIQIETVEEASALSTDTASDLSSKLETASATIDKRAVVSAQSDGQPNVRSGSYSVLPTSKSSVAPVAQITDGQIQHQTTASAVAPVAQITDGQIQHQTTAAASVVNQIGDGQIQHQTSAASVVNQIGDGQIQHQTTAASVINQIGDGQIQHQTTAASAINQIGDGQIQHQTSAKPTASAAAQISDGQIQHKNSTVAAESRAAATTLADGQPQESGASSDSSDSSTPQACSSSNNLVMKLEDSVLTDSHGRIGAIVANRQFQFDGPPPQAGSIFAAGWSISKDGYLTLGDSKVFYQCLSGDFYNLYDENVATQCNAVKLKIIDFVDC |  |
| *CmPIR11-1* | OL539426;  *CmPIR11-2* = OL539427 | B | 1092 | 363 | 18 | 0, 0 | 10, 3% | 2, 0 | 7, 4 | MLSPRLLASFATINLIHAYYIPATGEDWSKYKPSCDYLPESFTTLPFKFGIVVNPYIVNTEGELLEPEVKSIERSITTSFVTSVITPAPKKTKTKDIIVQITDGQVQKVNEELCDHEDKKKLGWEKEEDYDHHKYDDDKHFDGKFDKQFEEKDFETVLSKRDELDEADVGGDIKDKPFDEHSKHYKGKHGLKDDDLDFEKEYYNEHERSKEEHHEESYAEHRDFKERGGHDQHNHKGSHDEKDHKGGVHHHHDDDEFTSPVYSVACYTNATLRMTLQDSILRDSDDRIGCIVSGHQFQFDGPTPQHGAVFAAGWSVTKDGQLALGNSTKFYQCASGHFYNLYDQSIGFQCHPVTLDVVELIDC |  |
| *CmPIR23* | MT017925 | D | 780 | 259 | 19 | 0, 1 | 18, 7% | 0, 0 | 19, 4 | MQYSAILSVAFALISTSQAGYVKPRPITEPDCEPTTTPCAPPTTSTPCAPTTTSCCQDSDITLFSGEFALGVKELCDEDDVIYLVFELPDGQLEHNGVTVDCGCHPTTTPCAEPEPITDCDDKEKRWYKPKPKPTPTPSPYGLSNLCHPYCSIAATSDCTTDFFTLCDSILKDGKYRTGEIVANHQFQFDSPVQPDALYNKGWSIEYKDDYYLLALNECTTFYECPVDDCGLWKLYDASIDSKCKEIEIVIIFKDEECD |  |
| *CmPIR21* | MT017923 | K | 717 | 238 | 18 | 1, 0 | 8, 3% | 0, 0 | 0, 0 | MKFSSIFATFGLLAISQSSLVKRGGGEDNYHPLPAPTTPVVPHHPKPHYPEQFTLGIKVDGQIWLTFQEGDGQIEYDSIKYYPPSKPHWGDHHGTFTIDPTIEDGSEPSGHSDWKRGYDDADDIGDTGASYHPNPFTIKYQPWLSVYTLKNTVLKDSHGRIGSIVANHQFQFDNPVQPDALYTSGFSIEYENGYPLLALNGETTFWDSQASSSIWKLYDKPITNKSRKVELVVIKVIY |  |
| *CmPIR22* | MT017924 | L | 717 | 238 | 19 | 0, 1 | 11, 5% | 0, 0 | 3, 0 | MKFATALFIAFGFSAVANSSLIQRGYDDGDNHPTPPPQQQGVTSFPNKFALGAKVGEDIYVVFELSDGQVEYGTKKYTVPCVTPTPIIEPCDTPTPTPTTEPCEEPTPEPITFTHPHPHKPWKRDGFDFDGDDSWLTFESSYGYDWYTLKNSVLRDSKDRIGEIAANHQFQFDLPVQPDALYTSGFSIVHDDGYWLLALNGKTSFWDSPVNDNGVYKIYNAPINPKSRQIELVVIVLV |  |
| *CmPIR24* | MT017926 | None? | 780 | 259 | 19 | 0, 1 | 20, 8% | 0, 0 | 18, 4 | MQYSAILSVAFALISTSQAGYVRPRPITEPDCEPTTTPCAPPTTTTPCPPTTTSCRQDSDITLFSGEFALGVKELCDEDDVVYLVFELPDGQLEHNGVTVDCGCHPTTTPCAEPEPITDCDDKEKRWYKPKPKPTPTPSPYGLSNLCHPYCSIAATSDCTTDFFTLCDSILKDGKYRTGEIVANHQFQFDSPVQPDALYNKGWSIEYKDDYYLLALNECTTFYECPVDDCGLWKLYDASIDSKCKEIEIVIIFKDEECD |  |
| *CmPIR25* | MT017927 | None? | 786 | 261 | 19 | 0, 1 | 19, 7% | 0, 0 | 18, 4 | MQYSAILSVAFALISTSQAGYVKPRPITEPDCEPTTTPCAPPTTTTPCPPTTTSCRQDSDITLFSGEFALGVKELCDEDDVVYLVFELPDGQLEHNGVTVDCGCHPTTTPCAEPEPITDCDDKEKRWYKPKPKPTPTPTPSPYGLSNLCHPYCSIAATSDCTTDFFTLCDSVLKDGKYRTGEIVANHQFQFDSPVQPDALYNKGWSIEYKDDYYLLALNECTTFWECPVDDCGLWKLYDASIDSKCKEIEIVIIFKDEECD |  |
| ***Candida auris* B8441** | | | | | | | | | | | |
| *Cau004786* | B9J08_004786, PIS49759 | A | 867 | 288 | 16 | 0, 1 | 11, 4% | 0, 0 | 4, 4 | MQFKLSVLALISSALAAYVPSEPWTDLTPEGSIASATTDYTAKFGIQIVTLTSSAAAEETKAAKRDVINQIGDGQIQHQSASSTAQVVNQIGDGQIQHQTAAPPQPTSAQVVNQIGDGQIQHQTASVVNQIGDGQIQHQTATASVINQIGDGQIQHQTTAAAAASQIGDGQVQATDAPAAEHKGGAALQACMADNNLAMTLEKSILRDGSGRVGAIVANRQFQFDGPPPQAGSIYAAGWSITQEGLLALGNGTEFFQCKSGDFYNLYDQNIAEQCEPVHLSIVDLIKC |  |
| *Cau005431* | B9J08_005431, PIS48729 | (B) | 939 | 312 | 17 | (2), 1 | 2, 0.6% | 0, 0 | 7, 4 | MLIKSLLTSFFTSSVLAVWDPASGGDWAKLKPWAQKPDGAVSSLPFPVGIVTVPFQRNEKGQWEEPSLEEPPLWVPDVKEEFEAKWADIHQMKEGQALRAEYWMNEDSQEDDDDDNDDDDEPWELKGPDCEEKNWFDESWRLEHDYDVSDDADCEHSDSDYDSDYDKNANGNVVGFQVHVVPDVALKKRGHGVKKRALQAFKYPVKYSSCSDDGTLVMHLNGGILTDHLDRIGSIVSNHQFQFDGPVPQYGAIYAAGWSVSKDSLLCLGDRTTFYQCSAGDFHKTYDSPIHDECFPVHLEVVRIEGPCDDNF |  |
| *Cau003910* | B9J08_003910, PIS52296 | M | 1170 | 389 | 17 | 1, 0 | 42, 11% | 0, 0 | 4, 4 | MKFSALAAIVAAVAAAPASISNTWSTLTPTGTLNESASAITSISGSYALSIRTVSASVSQGISQDKRDVVSQIGDGQIQANSNTEVSKPPATTTASVINQIGDGQVQQQTASVVNQIGDGQIQQQTTASVVNQIGDGQIQQQTTASVVNQIGDGQIQQQTTASVVNQIGDGQIQQQTQTASVVNQIGDGQIQQQTAAPKSASVVDQIGDGQIQQHTTASVANQIGDGQIQQQSVAAESTHAPSQSVVNQVTDGQVQATGAASSDPDSPKDYFEETCVSDDSLLVSLTDGELRDSKGRVGAIVANRQFQFDGPPPQAGTIYAAGWSFVPADVAGLGEKQSDTKEGGFKLALGDQTTFYKCLSGDFYNLYDESIGAQCSAIEIFLLKAVDC |  |
| ***Clavispora (Candida) lusitaniae* ATCC 42720** | | | | | | | | | | | |
| *Cl05291* | CLUG_05291, XP_002614514 | A | 969 | 322 | 16 | 1, 0 | 41, 13% | 0, 0 | 4, 4 | ~~MSGPIWINVFKQLQSFPIDVFFITTNNNYIHQST~~ MKYSLTALALAAGSLAATITSEPWTTLTPTATFSGAKTDHTAKFGIQIVTFSSTSSSASATPSAEKRDVVNQIGDGQVQKQTSETLSSVPATTTAPVVNQIGDGQIQHQTVSVVNQIGDGQIQHQTASVVNQIGDGQIQHQTASAVNQIGDGQIQHQTTTATTATGAAQISDGQVQASATASPDGVIPEACLTSDSLVMELKNSVLTDGHGRIGAIVANRQFQFDGPPPQAGSIYAAGWSITDDGLLALGDSDVFYQCKSGDFYNLYDENVAKQCEAVHLSVIDLVSC |  |
| *Cl00824* | CLUG_00824, XP_002619665 | M | 1158 | 385 | 18 | 1, 0 | 4, 1% | 0, 0 | 4, 4 | MKFQSVALVSILASLASSAPVSPSNTWSTLTPTATLPKSASAITSTSGTFALSIRTVSASVSSGISVNKRDVISQISDGQVQADTKSATATASVINQISDGQVQENTKTTKKPEPTASVINQISDGQIQEQTKKTTASVINQIGDGQIQQQTKAKTTASVINQIGDGQIQQQTKAKPTPSASVINQISDGQIQQQTKTKTQKNTASAVAQISDGQIQEQTKTKTQKNTASAVAQISDGQPQQHTSAPVASQVSDGQVQATSSPDEATVEETCYDSDALTIQLKDGELRDSKGRVGAIVANRQFQFDGPPPQAGTIYAAGWSFVPASYAGVNEKSSDTTDEGLKLALGKQTVFYKCLSGDFYNLYDESIGDQCSAIEIFVLEAVQC |  |

| ***Yamadazyma (Candida) tenuis* strain ATCC 10573** | | | | | | | | | | | |
| --- | --- | --- | --- | --- | --- | --- | --- | --- | --- | --- | --- |
| *Yt113304* | CANTEDRAFT_113304, XP_006685822 | A | 837 | 278 | 14 | 0, 1 | 28, 10% | 0, 0 | 4, 4 | MLPLLYFVAVTIAAYVPSDNYSTLTPSASPTGTTDYTQSFGIQIETVSAAPSAAAKREVTQIGDGQIQAPTTTTSAAVTKVINQIGDGQIQNQPTASVINQIGDGQIQNQATTATTATKVVNQIGDGQIQNQPTTSASASVINQINDGQIQNQGTAAAQITDGQVQNNSTSDDTSGAKLETCATSDSLSMSLSASILTDAKGRIGSIVANRQFQFDGPPPQAGAIYAAGWSIIDGYLALGNNQTFYQCLSGDFYNLYDQDVADQCAEVKLAIVDLISC |  |
| *Yt115181*^§^ | CANTEDRAFT_115181, XP_006688804 | B | 582 | 193 | 21 | 0, 0 | 2, 1% | 0, 0 | 4, 4 | MHLMNVLLATMVAAIYIPANGDDWTLLKPEDAGAGIPTLDFDFGIVVSLVDEGEFANSNFNAIQNTVGQVRSQEEEVSLVSPAPAEATFDIKMVSCKTNSTLSMSLTGGILRDSSNRIGTMVSNRQFQFDGPTPQFGAVYANGWAADPDGYLVLGSQRVFYQCASGEFYNLYDQQIDTQCSPVNLRVVGLVEC |  |
| *Yt129279* | CANTEDRAFT_129279, XP_006684397 | M | 990 | 329 | 17 | 0, 2 | 30, 9% | 0, 0 | 5, 4 | MKFVCLLSIVAIANAAAVSSDVWKSLTPTTTLGSSAHATSSLPGSYALSIRTISSADASGSVTGLAKRDDIVTQIDDGQIQQVTNEAVASVVNQISDGQIQLQTTATVLNQISDGQIQAQTTATVLNQISDGQIQAQTATVVNQISDGQIQQQTADVVNQIDDGQVQQQTSTIAAVNQISDGQIQQQTSTAAALNQISDGQVQAASTATVDAASQISDGQIQDATSTSEAPDTNVTETCVASGSLLLQLKDGILTDAKGRVGSIVANRQFQFDGPPPQAGAIYAAGWSVVPIDYKEDSKTKENADKGKRNELYKLALGAQTTFYKCLSGSFYNLYDQSIGGQCSEVEFVVLEAVEC |  |
| ***Spathaspora passalidarum* NRRL Y-27907** | | | | | | | | | | | |
| *Sp146230* | SPAPADRAFT_146230, XP_007372629, Repaired | B | 1323 | 440 | 21 | 0, 2 | 13, 3% | 0, 0 | 5, 4 | MYVLSLLTITILLAHIFPISGYYVPANGDDWTKLKPNCEPLEGSFDTIPFVFGIVVNPYFINEEGELEEPPISSIERSVTTSFITSIVTAAPKPTKTKDLIHQIHDGQVQKVKSKEDFDVEAAAEVAPPPPPQLLKRSHHEADDIVVGQDLNYDDRIDSLEEIYEDIGGENKNRENRYKIQRGSRIDKKRPFEDDDKDSHRYHDDERDNHRYHDGKDRENHRYHDGDDKNNNRYRDGDDKNNNRYRDGDDKNNNRYRDGDDKNNNRYRDGDDNHKHYDDKNKDNNRYRDGDDNHKHYDDKDKDNHRHQDDDNYHKYPHEEDHHRYYPDHEEEEFISPVYAVACYGNSTLRMTLKDGILRDSDNRIGSIVSGHQFQFDGPTPQHGAVYAAGWSITKDGQLALGDSTKFFQCASGNFYNLYDEPIGYQCHPVTLDVVELIEC |  |
| *Sp151845* | SPAPADRAFT_151845, XP_007375071 | F | 831 | 276 | 19 | 0, 2 | 21, 8% | 0, 0 | 8, 4 | MKFSTSAGIGLALFSIAHAGYIKDAWTPKPPSDCYGASDGAVTDVSYKFELTVKVLSNNQKRGHEGYVGTTVTTTALTTLITTECSTSTTAPPPPPPPPPPPPPPTTTTYPETCYTPVVTQEPDGQAEFPDFTILPDTLPTNTAVPDDDDVIFLKRWDQQGCGKDKDGFYHTCITKNALLLNLHDSILTDANGRIGSIVANHQFQFDGPPPQPDSLYTSGWSIINQDGNYLLALGKQTVFWECAAGGFFKLYDASIGAQCKQVELVVLKADYCQKW |  |
| *Sp60896* | SPAPADRAFT_60896, XP_007375072 | M | 816 | 271 | 15 | 0, 0 | 13, 5% | 0, 0 | 4, 4 | MKLFNLIILALSVNAAPVVDSSFTTKTPTDVTRTAGGLVLSDGSFRTALFDFNGDLTLSVQTLDPNEKDIGPAPSGSLNVGQVSDGQVNQISDGQVNAANQKPDGQVNAVSQISDGQVNAANQKPDGQVNAVSQISDGQVNAASQISDGQVNSNQAQDGQVTASGGFSSVCVSGNSLIVKLSNGILTDSHGRIGSIVANHQFQFDGPPPQPDSLFAAGWNIYPSAQGPLLALGGQYIFWRCAIQGTTFNLYDASIDNKCEKVVIHLFAVSC |  |
| ***Scheffersomyces (Pichia) stipitis* CBS 6054** | | | | | | | | | | | |
| *Ss35989* | PICST_35989, XP_001384530 | A | 849 | 283 | 18 | 1, 0 | 29, 10% | 0, 0 | 4, 4 | MKYSRVQLLTALIGSSYAATIPSEPWTALTPSDAPPSGATTDHTHTFGIQIVTVTTSSEVSATSAVAKRDVVNQIGDGQIQKQTSETLATPTPTPSVQVINQIGDGQIQHQTTTAAEVINQIGDGQIQHQTTTAAEVINQIGDGQIQHQTTTAAENTVSIASQISDGQVQQPTDASSGDDDTPDACLTDNSLAMVLEGSVLRDSHGRVGAIVANRQFQFDGPPPQAGSIYAAGWSITKEGNLALGSVDTFYQCLSGDFYNLYDENVAAQCSPVKLNIVDLVAC |  |
| *Ss48210* | PICST_48210, XP_001385354, Repaired | B | 816 | 271 | 17 | (1), 0 | 11, 4% | 0, 0 | 7, 4 | MLSSVLATLLLSSTVLSSPVWIPESNGDWTTLRPDSDPLPNSFGSVPFSFGIVVNPYQETEDGELEVPPVSTIARSLTTVFTTSVVTAAPKPTKVADIVQILDGQVQRVNVDAEPTDDSQVPEEEECEDEEEVLIEETEENSDSLDKRHDEECCDDDEEEEDKEFVSPVYAVACATNTTLQMTLQDTILRDSNNRIGSIVSGHQFQFDGPVPQHGAIYAAGWYITEHAQLALGNSTEFYQCASGDFYNLYHEPIGLQCNPVVLDVVELIEC |  |
| *Ss81594* | PICST_81594, XP_001382276 | M | 837 | 278 | 18 | 0, 1 | 9, 3% | 0, 0 | 4, 4 | MRAAELLSLFAATSLVSAAVIPRDDESWKTLTPSGSYLPDATTDYDGSFAISIIVLETPTPAKRDVANQIGDGQVQVQTTAAAVVNQIGDGQIQQQTQTVAVVNQIGDGQIQQQTQTVAAVNQIGDGQIQQQTNTIGDGQVQAQTATVAGQIADGQVQATPAAQAADGQVQSNVVVCVAADSLTATLQGSILRDNKGRIGAIVANRQFQFDGPPPQAGSIYAAGWSIVPDGNGGQQLALGDQTTFYRCLSGDFYNLYDESIGGQCSPIEIAILKATTC |  |
| ***Meyerozyma (Candida) guilliermondii* ATCC 6260** | | | | | | | | | | | |
| *Mg01804* | PGUG_01804, EDK37706 | A | 891 | 296 | 17 | 0, 1 | 32, 11% | 0, 0 | 4, 4 | MKYSTFASIALAGTAYAAYVPSDPWTDLTPSAPAPTGASTDHTHKFGIQIVTVSGASSAASSAPATTTAAKRDAVNQIGDGQIQKQTSVTLSTPTPTTTAKVINQIGDGQIQHQTATTAKVINQIGDGQIQHQTASVVNQIGDGQIQHQTASVVNQIGDGQIQHQTVSGASQIGDGQVQAPTGTATPSGDDDEGVPTACLTDNSLSMNLNGSILTDSKGRIGAIVANRQFQFDGPPPQAGSIYAAGWSITSDGNLALGDQDVFYQCLSGDFYNLYDQNVAAQCSAVHLSVIDLVSC |  |
| *Mg03087* | PGUG_03087, EDK38989 | B | 747 | 248 | 19 | 0, 1 | 1, 0.4% | 0, 0 | 6, 4 | MLFLSGITLALAYATSSLALYIPGADDWTKLIPDSVPLAGSYDTIPFSFGIVVTPFEQIDGEYTEPSFHGPKSVTTTYTSTVITDGPKPTKAVSPVKQIWDGQIQNGPEDDCDDDDETYGYGKRNEVYDDCDSDDEDDSGLFSSPVFSVACLGENTLQMSLRGGILRDAQNRIGSIVSSHQFQFDGPLPQHGTLYANGWSVSSKGRLALGNSTTFYQCASGDFYNLYDKKIAYQCNPVTLEVVELISC |  |
| *Mg04923* | PGUG_04923, EDK40825 | M | 1257 | 418 | 18 | (2), 1 | 63, 15% | 0, 0 | 4, 4 | MKIQFYLSILSVAYSVSAAYVPSAPWSTLTPTATLDSSASATASFDGSFALSVSTISTNSAAVSAASKVGAEKRDDLVGQITDGQIQHNSKGGQKSAPTKSVVNQITDGQIQQQTKTTAAVVNQISDGQIQQQSKTTAAVINQISDGQIQQQTKTTADLVNQISDGQIQQQTKTTAAVINQISDGQVQQQTKTTASVVNQISDGQVQHQTTAPAHKSVAEQLSDGQVQHQTKAGQATQVSDGQVQASGSSSSGNDSSSDSGFEETCVSSDSLTVTLKNGVLKDSKGRIGSIVANRQFQFDGPPPQAGAIYAAGWSIVPLDYKEDSKTKQLADDQKKGENEEKDKKESKDKKKDEKKSKDKKKDKKKDKRDDVKWGKLALGKQTTFYKCLSGDFYNLYDESIGGQCSEVELVVLKAVEC |  |
| ***Debaryomyces hansenii* strain CBS 767** | | | | | | | | | | | |
| *Dh2B13442g* | DEHA2B13442g, XM_457530 | A | 1077 | 358 | 18 | 0, 1 | 35, 10% | 0, 0 | 4, 4 | MRYSVIFTSTALIASTLAAYVPSEPWTTLTPTGSAPAGATTDHTHKFGIQVKTVESSVAISSTVAETDSAVTAAASASGAAKRDDVVNQIGDGQIQQQSAAAETASVVNQIGDGQIQQQTAAAETASVVNQIGDGQIQQQTAQPTPTASVVNQIGDGQIQQQTAQTASVVNQIGDGQIQQQTAQTASVVNQIGDGQIQQQTAAAETASVVNQIGDGQIQQQTSQSPTTATVASQINDGQVQQQTDSAKSSSDSDSSVPQSCVGTNSLGMQLNGSVLTDEKGRIGAIVSNRQFQFDGPPPQAGSIYAAGWSVTENGLLALGDQTIFYQCHSGDFYNLYDESIAEQCSAVHLSIVDLVEC |  |
| *Dh2A00946g* | DEHA2A00946g, XM_456379 | M | 1596 | 531 | 19 | (1), 1 | 122, 23% | 0, 0 | 4, 4 | MQLTFSLSVLTTLAVLTHAAYVPSQPWSTLTPSGTIDSASASSNYDGSFALSIKTLGKGSTGTSAAPAAPTGGSADETEASAVAGAVASDAAVSVPTAPADIGASAAPVESGATASVPTPASDVGASAPTGEAAALRKRSDIVNQLGDGQIQQGGGSGSDSGSGSDSGSGSGSSGDSGSGSGSGSGSSSEGSGTPGGASGSGSTPGSGSEGSGAPGGASGSGSGSEDSGAPGGASGSDSTPGSGSGSGAPGGASGSGSGSEGSGAPGGASGSGSGSEDSGAPGGASGSDSTPGSGSGSASEGSGSPGGASDSGSTPGSGSSSEGSGAPGGAGGNDSPSKTADVMNQIGDGQVQQQQQTGSPNGQAAAQSQEAGVTAVADKTAANVASQVNDGQVQQTGAADSKAVGTENGETCLKSDSLSVTLKDGVLRDSKDRIGAIVAGHQFQFDGPPPQAGTIYAAGWSVVPSDYSGSSSESSDSSNSKRSDSPGKLALGSQTTFYKCNSGDFYNLYDQSIGSQCEEVEVTILKAVEC |  |
| ***Candida glabrata* CBS 138** | | | | | | | | | | | |
| *CAGL0I06204g* | XM_447520, *PIR1*, *GVI51_I05995* | Unk | 1050 | 349 | 18 | 1, 0 | 35, 10% | 0, 0 | 4, 4 | MQYKKTLAASALATSALAAYVPGKPWSTLTPSATFKGGITDYASTFGIAVQPIATPASVAKRAVSQIGDGQVQATTKTTSTLAPKAPISQIGDGQIQATTKTQAAPVSQIGDGQIQATTKTKAAPVSQIGDGQIQATTKTQAAPVSQIGDGQIQATTKTQAAPVSQIGDGQIQATTKTQAAPVSQIGDGQIQATTKTKATGVSQIGDGQIQATKTGSTTPSQPPMNNSTIPVPNNNATLPVNAKDPVRAESCKVDGTLEMNLKGGILTDGKGRIGSIVANRQFQFDGPPPQAGAIFAAGWSLTPEGNLAIGDNDVFYQCLSGNFYNLYDQHIGSQCTPVHLSAIDLIKC |  |
| *CAGL0I06182g* | XM_447519, *PIR2*, *GVI51_I05973* | Unk | 1023 | 340 | 18 | 1, 0 | 32, 9% | 0, 0 | 4, 4 | MQYKKTLAASALATSALAAYVPGQPWSTLTPSATFKGGITDYASTFGIAVQPIATPASVAKRAVSQIGDGQVQATTKTTSTLAPKAPISQIGDGQIQATTKTQAAPVSQIGDGQIQATTKTQAAPVSQIGDGQIQATTKTQAAPVSQIGDGQIQATTKTQAAPVSQIGDGQIQATTKTQAAPVSQIGDGQIQATTKTAVSQIGDGQIQATKTATAGASQIQDGQVQASNQGKAPNDPVGAVSCKVDGTLEMNLKGGILTDGKGRIGSIVANRQFQFDGPPPQAGAIFAAGWSLTPQGNLALGDNDVFYQCLSGNFYNLYDQHIGSQCTPVHLSAIDLIKC |  |
| *CAGL0M08492g* | XM_449714, *PIR3*, *GVI51_M08437* | Unk | 1008 | 335 | 19 | 1, 0 | 23, 7% | 0, 0 | 4, 4 | MQYKKSLAATALLASSGLAAYVPGNPWSTLTPSGTYKGGLTDYSSTFGIAVQPIATSSSVAKRAVSQIGDGQVQAATKTQAAPVSQIGDGQIQATTKTQAAPVSQIGDGQIQAATKTQAAPVSQIGDGQIQAATKTQAAPVSQIGDGQIQAATKTQAAPVSQIGDGQIQATTKTQAAPVSQIGDGQIQATTKTTQAASQIGDGQVQAATKTASAASQIADGQVQQNKDPKDPVGAVSCKVDGTLQMNLKGGILTDEKGRIGSIVANRQFQFDGPPPQAGAIYAAGWSLTPQGNLALGDSDVFYQCLSGNFYNLYDQSIGAQCHPVHLSAIDLVKC |  |
| *CAGL0I06160g* | XM_447518, *PIR4*, *GVI51_I05951* | Unk | 699 | 233 | 18 | 1, 0 | 30, 13% | 0, 0 | 6, 4 | MQFKNVALTAAVASVAAADGYTPGNPWSTLTPSGTVACAKPEYTASFGIAVKPISSSVAKRAVVSQIGDGQIQATSAAPKPTNAAASQVADGQVQQKTTSTTKATTTLAPSSSKTSSTSTSTSCAATPLTIKESSCKNDGTLQLTLKGGVLTDGKGRVGSIVSNRQFQFDGPPPQAGAIYAGGWSITEQGNLALGNSDVFYQCLSGNFYNLYDQKIAEQCSPINLEVVSLVDC |  |
| *CAGL0M08514g* ^¦^ | XM_449715, *PIR5*, *GVI51_M08459* | Unk | 633 | 210 | 17 | 0, 0 | 34, 16% | 0, 0 | 4, 4 | MQFNKISLLAMASAASAATTSPTTTETKPTTTKFFTPGDNFSTFAPNATFSGAAVNFTSTFGIAVQSIDSKSVVSKSLVSTSTTSTLAPSSSTSSAASSTTSSPNITIVNKSCKADGTLVMELENGILRDGKGRIGSIVSNRQFQFDGPPPQAGAIYAAGWSVTPEGNLALGDSDVFYQCSSGNFYNLYDEYIAEQCHPIHLEVLSLVDC |  |
| ***Saccharomyces cerevisiae* S288C** | | | | | | | | | | | |
| *ScPIR1* | YKL164C, S000001647, CCW6 (*PIR5* paralog) | A | 1026 | 341 | 18 | 1, 0 | 43, 13% | 2, 0 | 4, 4 | MQYKKSLVASALVATSLAAYAPKDPWSTLTPSATYKGGITDYSSTFGIAVEPIATTASSKAKRAAAISQIGDGQIQATTKTTAAAVSQIGDGQIQATTKTKAAAVSQIGDGQIQATTKTTSAKTTAAAVSQIGDGQIQATTKTKAAAVSQIGDGQIQATTKTTAAAVSQIGDGQIQATTKTTAAAVSQIGDGQIQATTNTTVAPVSQITDGQIQATTLTSATIIPSPAPAPITNGTDPVTAETCKSSGTLEMNLKGGILTDGKGRIGSIVANRQFQFDGPPPQAGAIYAAGWSITPEGNLAIGDQDTFYQCLSGNFYNLYDEHIGTQCNAVHLQAIDLLNC |  |
| *ScPIR2* | YJL159W, S000003695, *HSP150*, *CCW7*, *ORE1* (*PIR3* paralog) | None | 1242 | 413 | 18 | 1, 0 | 95, 23% | 0, 0 | 4, 4 | MQYKKTLVASALAATTLAAYAPSEPWSTLTPTATYSGGVTDYASTFGIAVQPISTTSSASSAATTASSKAKRAASQIGDGQVQAATTTASVSTKSTAAAVSQIGDGQIQATTKTTAAAVSQIGDGQIQATTKTTSAKTTAAAVSQISDGQIQATTTTLAPKSTAAAVSQIGDGQVQATTTTLAPKSTAAAVSQIGDGQVQATTKTTAAAVSQIGDGQVQATTKTTAAAVSQIGDGQVQATTKTTAAAVSQIGDGQVQATTKTTAAAVSQITDGQVQATTKTTQAASQVSDGQVQATTATSASAAATSTDPVDAVSCKTSGTLEMNLKGGILTDGKGRIGSIVANRQFQFDGPPPQAGAIYAAGWSITPDGNLAIGDNDVFYQCLSGTFYNLYDEHIGSQCTPVHLEAIDLIDC |  |
| *ScPIR3* | YKL163W, S000001646, *CCW8* (*PIR2* paralog) | None | 978 | 325 | 18 | 1, 0 | 48, 15% | 0, 0 | 4, 4 | MQYKKPLVVSALAATSLAAYAPKDPWSTLTPSATYKGGITDYSSSFGIAIEAVATSASSVASSKAKRAASQIGDGQVQAATTTAAVSKKSTAAAVSQITDGQVQAAKSTAAAVSQITDGQVQAAKSTAAAVSQITDGQVQAAKSTAAAVSQITDGQVQAAKSTAAAASQISDGQVQATTSTKAAASQITDGQIQASKTTSGASQVSDGQVQATAEVKDANDPVDVVSCNNNSTLSMSLSKGILTDRKGRIGSIVANRQFQFDGPPPQAGAIYAAGWSITPEGNLALGDQDTFYQCLSGDFYNLYDKHIGSQCHEVYLQAIDLIDC |  |
| *ScPIR4* | YJL158C, S000003694, *CIS3*, *CCW11*, *CCW5*, *SCW8* | None | 678 | 225 | 21 | 1, 0 | 28, 12% | 1, 0 | 6, 4 | MQFKNVALAASVAALSATASAEGYTPGEPWSTLTPTGSISCGAAEYTTTFGIAVQAITSSKAKRDVISQIGDGQVQATSAAATDSQVQASSTATPTSSEKISSSASKTSSTNATSSSCATPSLKDSSCKNSGTLELTLKDGVLTDAKGRIGSIVANRQFQFDGPPPQAGAIYAAGWSITEDGYLALGDSDVFYQCLSGNFYNLYDQNVAEQCSAIHLEAVSLVDC |  |
| *ScPIR5* | YJL160C, S000003696 (*PIR1* paralog) | None | 864 | 287 | 21 | 1, 0 | 35, 12% | 0, 0 | 4, 4 | MHYKKAFLASLLSSIALTAYAPPEPWATLTPSSKMDGGTTEYRTSFGLAVIPFTVTESKVKRNVISQINDGQVQVTTQKLPHPVSQIGDGQIQVTTQKVPPVVSHIVSQIGDGQLQITTAKNVVTKSTIAVPSKTVTATATSTATAVSQIHDGQVQVTISSASSSSVLSKSKLEPTKKPNNEKVIKVQACKSSGTLAITLQGGVLIDSSGRIGSIVANRQFQFDGPPPQAGAIYAGGWSITKHGTLAIGDNDVFYQCLSGTFYNLYDQSIGGQCNPVHLQTVGLVDC |  |

* Columns display the gene name used in this paper; sequence identifiers from public databases; the ortholog group designated on the *Candida* Gene Order Browser (CGOB; Maguire et al., 2013) and given alphabetic labels in order of appearance in this table (OG); size of the gene in nucleotides; size of the predicted protein in amino acids; size of the signal peptide in amino acids as predicted by the SignalP-5.0 Server (highlighted in gray); the number of potential Kex2 cleavage sites predicted by ProP 1.0 (dark green) and the number not recognized by the program (purple)—numbers in parentheses indicate that the predicted Kex2 cleavage site is C-terminal of the DGQ motif; the number of O-glycosylation sites predicted by NetOGlyc – 4.0 and the percentage of the total amino acids in the protein; the number of N-glycosylation sites predicted by NetNGlyc-1.0 (red) and the number not recognized by the program (dark blue)—numbers in parentheses indicate that the predicted N-glycosylation site would be removed by signal peptide processing; the number of Cys residues in the protein and the number that align with the 4 conserved Cys residues found in the C-terminal portion of *S. cerevisiae* Pir proteins (highlighted in light green); and the protein sequence. DGQ motifs are highlighted in light blue. QFQFD motifs are highlighted in yellow.

† Ortholog group (OG) B is in parentheses for *Cau005431* because the gene is inverted in the genome, but near ortholog pillar B and likely part of that group. *CmPIR24* and *CmPIR25* did not appear to be included in the *C. metapsilosis* genome sequence used in CGOB so their ortholog designations were unclear. The *C. glabrata* genome was not included in CGOB so ortholog designations were unknown (Unk). *ScPIR1* was part of ortholog group A, but the other *ScPIR* genes did not have a designation. The arrangement of *PIR* genes in *S. cerevisiae* was shown in **Supplementary Figure S10**.

‡ Protein sequences are listed as they were found in public databases. Sequences were corrected in the context of this work. *CaCIS308*, *Cd15040*, *Cd30400*, *Cp205800*, *Co0D05920*, and *Cl05291* were annotated with an N-terminal Met that did not correspond to a secretory signal peptide. A putative signal peptide was found downstream for each protein. The full sequence was shown with strike-though used to designate the incorrect start codon and sequence. *Sp146230* and *Ss48210* were incomplete in the online databases. Examination of the genome sequence revealed an intact, upstream coding region that started with Met and included the Pir motifs. In each entry, the original sequence associated with the accession number is underlined. LELG_02549 initially appeared to lack the QFQFD motif. Examination of the genome sequence revealed a frameshift in the vicinity of the gray shaded area. Downstream sequences predicted a protein that included the QFQFD motif and were included in the table (underlined).

§ GenBank lists two alternative splice variants from the same locus in *Y.tenuis*. *XP_006688803.1* was longer; XP_006688804.1 was underlined within the XP_006688803.1 entry.

¦ *CAGL0M08514g* does not encode a DGQ motif.
