# Supplementary material for: Peering Into Candida albicans Pir Protein Function and Comparative Genomics of the Pir Family
Source: Front Cell Infect Microbiol. 2022 Mar 18;12:836632. doi: 10.3389/fcimb.2022.836632 (PMC8975586; doi:10.3389/fcimb.2022.836632)
Supplement: Supplementary file 5 [file Table_4.docx]

**SUPPLEMENTARY TABLE S4 |** *PIR1* allelic variation patterns in a diverse collection of *C. albicans* isolates.

Human Isolates Wildlife Isolates

Code* Clade *PIR1* Pattern Code Clade *PIR1* Pattern

1-21 1 A SqB056 1 A

1-39 1 A RbA025 1 A

1-5 1 A SqA010 1 A

1-24 1 A SqB030 1 A

1-25 1 A RcB036 1 A

1-93 1 E StA0524 1 A

1-151 1 A RbC078 1 A

1-161 1 A RcA013 3 C

1-216 1 A RcC0556 3 B

1-177 1 A SqC075 3 C

1-95 1 E RbB043 4 E

1-107 1 A DB053 8 E

1-86 1 A OpA052 8 E

1-238 1 A SqH001 8 E

1-204 1 A WcA017 8 E

1-44 1 A WcA019 8 E

1-23 1 A SqH099 8 E

1-52 1 A DA047 8 E

1-54 1 A OpA059 8 E

1-110 1 A OpA060 8 E

1-139 1 A SqD080 8 E

1-163 1 A RhA029 9 E

1-197 1 A SqF087 9 E

1-92 1 A SqE086 11 F

1-178 2 B SqE097 11 F

1-246 2 B CrA038 Sing F

1-32 3 C SqG098 Sing E

1-226 3 B

1-120 3 B

1-132 3 C

1-233 3 C

1-19 3 C

1-55 3 B

1-171 3 C

1-237 7 E

1-28 8 D

1-20 8 D

1-190 8 D

2-127 11 C

2-213 11 B

1-18 Sing B

________________________________________________________________________________

*Code is a strain identifier as described by Wrobel *et al*. (2008). Clade designations follow the phylogenetic groups proposed by Odds *et al*. (2007). Singleton strains (Sing.) did not clearly fit into any of the clades. *PIR1* patterns were designated A through F as defined in **Figure 6**.
