# Supplementary material for: Peering Into Candida albicans Pir Protein Function and Comparative Genomics of the Pir Family
Source: Front Cell Infect Microbiol. 2022 Mar 18;12:836632. doi: 10.3389/fcimb.2022.836632 (PMC8975586; doi:10.3389/fcimb.2022.836632)
Supplement: Supplementary file 6 [file Table_5.docx]

**SUPPLEMENTARY TABLE S5 |** *C. albicans* strains used in this study.

| **Strain** | **Parent** | **Genotype*** | **Source/reference** |
| --- | --- | --- | --- |
| SC5314 |  | Wild-type | (Gillum et al., 1984) |
| 3077 | SC5314 | *Δpir1-1*::*SAT1-FLIP*/*PIR1-2* |  |
| 3080 | SC5314 | *PIR1-1*/Δ*pir1-2*::*SAT1-FLIP* | This study |
| 3082 | 3077 | *Δpir1-1*::*FRT*/*PIR1-2* | This study |
| 3083 | 3080 | *PIR1-1*/Δ*pir1-2*::*FRT* |  |
| 3087 | 3083 | *Δpir1-1*::*FRT*/*Δpir1-2::SAT1-FLIP* | This study |
| 3097 | 3087 | Δ*pir1-1*::*FRT*/Δ*pir1-2*::*FRT* | This study |
| 3511 | 3097 | Δ*pir1-1*::*FRT*/Δ*pir1-2*::*FRT*  *Δpir32-1::SAT1-FLIP*/*PIR32-2* | This study |
| 3516 | 3511 | Δ*pir1-1*::*FRT*/Δ*pir1-2*::*FRT*  *Δpir32-1::FRT*/*PIR32-2* | This study |
| 3520 | SC5314 | *PIR32-1*/Δ*pir32-2*::*SAT1-FLIP* | This study |
| 3521 | SC5314 | Δ*pir32-1*::*SAT1-FLIP/PIR32-2* | This study |
| 3524 | 3521 | *Δpir32-1::FRT*/*PIR32-2* | This study |
| 3525 | 3520 | *PIR32-1*/*Δpir32-2*::*FRT* |  |
| 3537 | 3524 | Δ*pir32-1*::*FRT*/Δ*pir32-2*::*SAT1-FLIP* | This study |
| 3540 | 3516 | Δ*pir1-1*::*FRT*/Δ*pir1-2*::*FRT*  Δ*pir32-1*::*SAT1-FLIP* /Δ*pir32-2*::*FRT* | This study |
| 3543 | 3540 | Δ*pir1-1*::*FRT*/Δ*pir1-2*::*FRT*  Δ*pir32-1*::*FRT*/Δ*pir32-2*::*FRT* | This study |
| 3545 | 3537 | Δ*pir32-1*::*FRT/*Δ*pir32-2*::*FRT* | This study |

*The *C. albicans* genome assembly is presented as diploid alleles (Skrzypek et al., 2017; [www.candidagenome.org](http://www.candidagenome.org)). **Supplementary Figures S15 and S16** show the wild-type *PIR1* and *PIR32* alleles, respectively, as well as selected depictions of mutant strains derived during the process of deleting various *PIR* alleles.
