# Supplementary material for: Peering Into Candida albicans Pir Protein Function and Comparative Genomics of the Pir Family
Source: Front Cell Infect Microbiol. 2022 Mar 18;12:836632. doi: 10.3389/fcimb.2022.836632 (PMC8975586; doi:10.3389/fcimb.2022.836632)
Supplement: Supplementary file 7 [file Table_6.docx]

**SUPPLEMENTARY TABLE S6 |** Plasmids used in this study.

| **Plasmid** | **Characteristics** | **Reference** |
| --- | --- | --- |
| 3027 | pSFS2, encodes *SAT1* flipper deletion cassette | (Reuß *et al.*, 2004) |
| 3054 | Plasmid 3027 with *PIR1* downstream sequence inserted between *Sac*II and *Sac*I | This study |
| 3059 | Plasmid 3054 with *PIR1* upstream sequence inserted between *Kpn*I and *Xho*I | This study |
| 3502 | Plasmid 3027 with *PIR32* upstream sequence inserted between *Kpn*I and *Xho*I | This study |
| 3505 | Plasmid 3502 with *PIR32* downstream sequence inserted between *Sac*I and *Sac*II | This study |
| 3529 | Plasmid 3502 with *PIR32* new downstream sequence inserted between *Sac*I and *Sac*II | This study |
