# Supplementary material for: Peering Into Candida albicans Pir Protein Function and Comparative Genomics of the Pir Family
Source: Front Cell Infect Microbiol. 2022 Mar 18;12:836632. doi: 10.3389/fcimb.2022.836632 (PMC8975586; doi:10.3389/fcimb.2022.836632)
Supplement: Supplementary file 8 [file Table_7.docx]

**SUPPLEMENTARY TABLE S7 |** Primers used in this study.

| Primer (Number*) and Name | Sequence (5’ 🡪 3’) |
| --- | --- |
| (1) PIR1-upF | CCCAAGCTTGGTACCCTATAATCTGCATCGATTAAG |
| (2) PIR1-upR | CCCCTCGAGAGTTGATGTTATTATAGTTGT |
| (3) PIR1-dnF | CCCCCGCGGGTTAGTTATTCTGGAGAAGAT |
| (4) PIR1-dnR | CCCGAGCTCAACTAAATCAACTTCAACTTC |
| (5) PIR1 DnOrfCheck F | TGGTCGTGACCAAAGAACAA |
| (6) PIR1 DnOrfCheck R | GCTGTTGATTGGTGGACATTAG |
| (7) PIR1-CDF | CCCGCCGGCATGAAGTATTCTACACT |
| (8) PIR1F2 | TCAGTGATGGTCAAATCCAAC |
| (9) PIR1R1 | GTTGGATTTGACCATCACTGA |
| (10) PIR1F3 | ATCCAATTCAACTTTATCTGA |
| (11) PIR1R2 | TCAGATAAAGTTGAATTGGAT |
| (12) PIR1A | TTCTACACTTGTTAGTATTGCTGCT |
| (13) PIR1B | TCAATGTAGAAGTAGCAGTATCAGTT |
| (14) PIR1C | ACTGATACTGCTACTTCTACATTGA |
| (15) PIR1D | GTCAGATTGAGCTTTAGCAACATTT |
| (16) PIR1G | AAATGTTGCTAAAGCTCAATCTGAC |
| (17) PIR1H | ACAGTTGACAAATTCAATGACACT |
| (18) PIR32 Kpn upF | CCCGGTACCAGCCTTGGGTTGTGATAAAT |
| (19) PIR32 Xho upR | CCCCTCGAGAAAGAATACTGTGTGTCGGT |
| (20) PIR32 SacII dnF | CCCCCGCGGCTCTAATATGAAAGAAGATG |
| (21) PIR32 SacI dnR | CCCGAGCTCAGGTCTCTGGTTAAAGCTATC |
| (22) PIR32 SacII FII | CCCCCGCGGAAACATGAGAAATCGGAAGGTTATG |
| (23) PIR32 SacI RII | CCCGAGCTCGTAACAGGATGACATTGGAAAGC |
| (24) PIR32 allA F | CCAGCAAATGGTGATGATTGGACAATATTA |
| (25) PIR32 allA R | CTTCTTCTTCTTCATTGATCTGTTGATTT |
| (26) PIR32 allB F | CCAGCAAATGGTGATGATTGGACAATTTTG |
| (27) PIR32 all B R | CTTCTTCTTCTTCATTGATCTGTCGATTC |
| (28) PIR32 CDRF | aagtatataccgacacacagtattc |
| (29) PIR32 AF | AAATACTAGTCTATTCACCTTTCTCAT |
| (30) PIR32 BR | CAGTAGAAGTAGTAATTATTGAAGTAA |
| (31) PIR32 CF | TTTGTTACTTCAATAATTACTACTTCTA |
| (32) PIR32 DR | TCTTGTGCGTTATTGTCTTCTATTT |
| (33) PIR32 HR | Aacatatacgtagatagggagatcata |
| (34) PIR32 CDRR | tgatgatgataggtctctggttaaag |
| (35) NatCheck F | CGACCAAGGCTTTGAACTATCT |
| (36) PIR1LocusUpF | cggaagggagatttgatcgttag |
| (37) ORF19.2782 UPF | cagtggatataaatcgtgattgttgg |
| (38) ORF19.2784 DNR | ggaaggatatgctgtcggaatg |
| (39) PIR32InsrtCheckR | GGAGACTTGTCTGGTTGATTGA |
| CmPir01-F | TTTCCCTCGACCCTTTCTTT |
| CmPir01-R | GAAACAACCTCCCACCAGAA |
| CmPir01-seqF1 | CTGCATCTGCTGTTGCTCC |
| Cm_1_954913 NT-F1 | TGGCTGTCTGAAAGGTAGC |
| Cm_1_954913 CT-R1 | CACTATGGGCATGCGACT |
| CmPir21-F | GTCAATAGTCAAGTCTACAAGCTTG |
| CmPir21-R | GACAATCAATTTGGAACACACAAG |
| CmPir22-F | ATGGTGACAACCACCCAA |
| CmPir22-R | TGAATTCAACCACACTGCTT |
| CmPir22-F1 | CCTCCCATGAACACCCTATTT |
| CmPir22-F2 | CCCAACTCCACCACCAC |
| CmPir22-R1 | GGTAGTGATAGGGTAAGGACAATC |
| CmPir22-R2 | CCGAGTTGACGGCTATCTTT |
| CmPir23-F | GATATGACCACCCGCTTTGA |
| CmPir23-R | CTAGCAGAACCAGCATATAGACC |
| CmPir24-F | CTGTGTGTGATACGAACACCT |
| CmPir24-R | CCCTGAAGTATAAAGCCAACTAGA |
| CmPir25-F | CCCGCTTTGATTTACCACATTC |
| CmPir25-R | GATCACCAATATGACCGAAATGAAA |
| CmPir25-F1 | CGGAGGCGGAGAAGATTAAA |
| CmPir25-R1 | GGGCACTTAATAAGGGTACTCAA |
| CmPir25-R2 | GGGTAGACACACTATTCTGCAA |

*Primer numbers refer to notations in **Supplementary Figures S12 and S14 through S18**. Primer names that begin with Cm were used for PCR amplification and DNA sequence verification of *C. metapsilosis PIR* genes.
